# Proteome and Peptidome of *Vipera berus berus* Venom

**DOI:** 10.3390/molecules21101398

**Published:** 2016-10-19

**Authors:** Aleksandra Bocian, Małgorzata Urbanik, Konrad Hus, Andrzej Łyskowski, Vladimír Petrilla, Zuzana Andrejčáková, Monika Petrillová, Jaroslav Legath

**Affiliations:** 1 Department of Biotechnology and Bioinformatics, Faculty of Chemistry, Rzeszow University of Technology, Powstańców Warszawy 6, 35-959 Rzeszów, Poland; murbanik92@wp.pl (M.U.); knr.hus@gmail.com (K.H.); andrzej.lyskowski@prz.edu.pl (A.Ł.); Jaroslav.Legath@uvlf.sk (J.L.); 2 Department of Pharmacology and Toxicology, University of Veterinary Medicine and Pharmacy, Komenského 73, 041 81 Košice, Slovakia; 3 Department of Physiology, University of Veterinary Medicine and Pharmacy, Komenského 73, 041 81 Košice, Slovakia; petrillav@gmail.com (V.P.); zuzka.kravcova@gmail.com (Z.A.); 4 Zoo Košice, Široká 31, 040 06 Košice-Kavečany, Slovakia; 5 Department of General Education Subjects, University of Veterinary Medicine and Pharmacy, Komenského 73, 041 81 Košice, Slovakia; monika.petrillova@uvlf.sk

**Keywords:** *Vipera berus berus*, venom, proteome, peptidome, cytotoxicity

## Abstract

Snake venom is a rich source of peptides and proteins with a wide range of actions. Many of the venom components are currently being tested for their usefulness in the treatment of many diseases ranging from neurological and cardiovascular to cancer. It is also important to constantly search for new proteins and peptides with properties not yet described. The venom of *Vipera berus berus* has hemolytic, proteolytic and cytotoxic properties, but its exact composition and the factors responsible for these properties are not known. Therefore, an attempt was made to identify proteins and peptides derived from this species venom by using high resolution two-dimensional electrophoresis and MALDI ToF/ToF mass spectrometry. A total of 11 protein classes have been identified mainly proteases but also l-amino acid oxidases, C-type lectin like proteins, cysteine-rich venom proteins and phospholipases A_2_ and 4 peptides of molecular weight less than 1500 Da. Most of the identified proteins are responsible for the highly hemotoxic properties of the venom. Presence of venom phospholipases A_2_ and l-amino acid oxidases cause moderate neuro-, myo- and cytotoxicity. All successfully identified peptides belong to the bradykinin-potentiating peptides family. The mass spectrometry data are available via ProteomeXchange with identifier PXD004958.

## 1. Introduction

Venom is a complex mixture of various chemicals that are used to kill or immobilize the victim and eventually help digestion. These substances affect nervous, muscular and cardiovascular systems. Most of the toxic substances, as much as 95%, contained in the venom of snakes are polypeptides: enzymes and non-enzymatic proteins. Depending on the genus, snakes produce venom of different composition and mechanisms of action, but within the family it has similar composition [1].

*Vipera berus berus* or common European adder is found in Europe and Asia in the areas of wetlands, peat bogs and forests, where they can find sunny slopes and glades. Depending on the area in which an individual resides, coloration varies from gray, blue-gray, brown, green-brown, red-brown to black. On the back, a distinctive dark zigzag is present, and on its head a dark stain in the shape of the letter H, V or X. The head is clearly separated from the trunk, triangular, flattened, and covered with tiny plates [2].

Venom of the common European adder is a yellow liquid consisting of approximately 25 proteins and peptides with enzymatic activity. Total composition of it is not fully known. Venom ingredients immobilize the victim and initialize digestion of the tissue near the site of the bite. The venom has hemolytic, proteolytic and cytotoxic properties. It consists of: protease, phospholipase, hyaluronidase, metalloproteinases, phosphodiesterases and l-amino acid oxidase. The presence of these families of compounds cause edema, disruption of homeostasis and hypovolemia [3,4].

Only a few of venom components are described in the *V. berus berus* species. Presence of the most of the venom components is inferred from the properties of the venom itself. Currently venom of many snakes is intensively studied because of the huge variety of proteins that occur there. Knowledge of the venom proteome and biological properties of the individual components may constitute a valuable source of new drugs. Collected information might also help in new drug design for use in the treatment of cardiovascular diseases, nervous system disorders, or cancer [1].

The aim of the study was to determine the composition of venom protein and peptide produced by adult *V. berus berus* and it is the first such a full proteomic description for this species.

## 2. Results

### 2.1. Proteome

**Figure 1:**
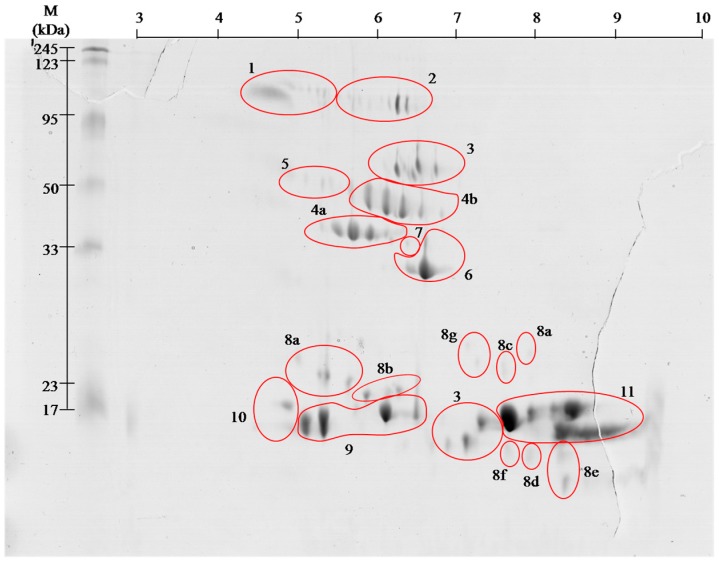
Representative 2-D protein map in 3–10 pH range, obtained from *V. berus berus* venom with identified protein groups shown: **1**, Angiotensin-like peptide; **2**, Metalloproteinase H3; **3**, l-amino acid oxidase; **4**, Serine proteases: (**a**) VLSp and (**b**) nikobin; **5**, Beta-fibrogenase brevinase; **6**, Cysteine rich venom protein; **7**, Snake venom metalloproteinases; **8**, Snaclec: (**a**) rhinocetin, (**b**) snaclec 14, (**c**) snaclec B6, (**d**) echicetin, (**e**) snaclec 1, (**f**) rhodocetin/A13, and (**g**) jerdonibitin; **9**, Acidic phospholipases; **10**, Basic phospholipases; and **11**, Neutral phospholipase. The proteins were separated by isoelectrofocusing at pH range 3–10, then distributed on polyacrylamide gels by SDS-PAGE and stained with colloidal Coomassie Brilliant Blue G-250. Molecular weight (MW) and pH 3–10 scale are shown.

**Figure 2:**
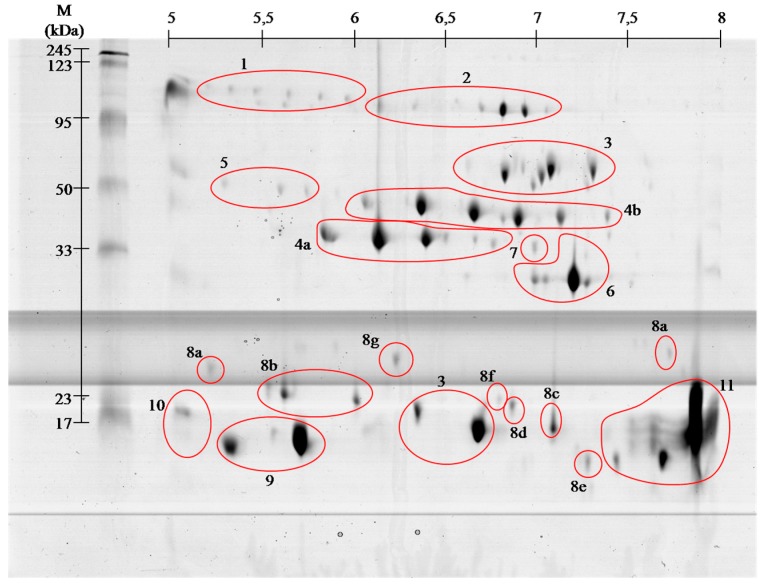
Representative 2-D protein map in 5–8 pH range, obtained from *V. berus berus* venom with identified protein groups shown: **1**, Angiotensin-like peptide; **2**, Metalloproteinase H3; **3**, l-amino acid oxidase; **4**, Serine proteases: (**a**) VLSp and (**b**) nikobin; **5**, Beta-fibrogenase brevinase; **6**, Cysteine rich venom protein; **7**, Snake venom metalloproteinases; **8**, Snaclec: (**a**) rhinocetin, (**b**) snaclec 14, (**c**) snaclec B6, (**d**) echicetin, (**e**) snaclec 1, (**f**) rhodocetin/A13, and (**g**) jerdonibitin; **9**, Acidic phospholipases; **10**, Basic phospholipases; and **11**, Neutral phospholipase. The proteins were separated by isoelectrofocusing at pH range 3–10, then distributed on polyacrylamide gels by SDS-PAGE and stained with colloidal Coomassie Brilliant Blue G-250. Molecular weight (MW) and pH 3–10 scale are shown.

The combined venom from adult *Vipera berus berus* individuals (male and female) was separated by two-dimensional electrophoresis in two pH ranges, 3–10 and 5–8. From the obtained polyacrylamide gels all visible spots were cut out, and then subjected to tryptic digestion procedure. All samples were analyzed by mass spectrometry MALDI ToF/ToF. Polyacrylamide gels show that the most proteins of this venom are concentrated in the pH 5–8, and only a few, having a molecular weight below 20 kDa, fall outside the above range of pH (Figure 1 and Figure 2).

On the basis of performed identification, proteins have been grouped according to their class. Proteins were grouped by combining the results of both pH ranges of 3–10 (Figure 1), and 5–8 (Figure 2) separation. The numbers on gels correspond to the different classes of proteins. On the gels with broader range of pH, proteins having an isoelectric point above pH 8 can be seen, whereas no proteins were observed in pH below 4.

Complete list of identified proteins is summarized in Table 1. Representative MS and MS/MS spectra for all identified proteins and peptides have been included as Supplementary Materials.

**Figure 3:**
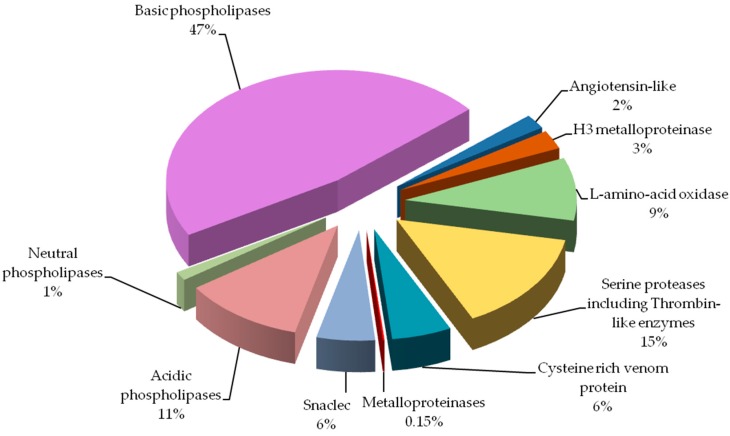
Protein groups of *Vipera berus berus* venom. Each group is represented as a percent fraction of the particular protein spots present on the gels.

Percentage of protein groups in *Vipera berus berus* venom is presented in Figure 3. By far the largest share of the analyzed venom are phospholipases (almost 60%). Other groups containing a significant amount of protein are: serine proteases and l-amino-acid oxidase. Angiotensin-like potential protein and metalloproteinases have been detected in the lowest amounts.

### 2.2. Peptidome

**Figure 4:**
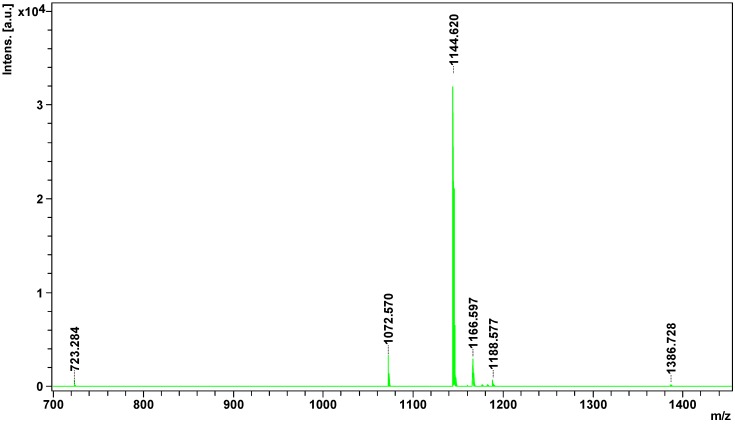
Mass spectrum of peptidome fraction of *Vipera berus berus* venom obtained on MALDI ToF/ToF mass spectrometer.

Peptides of less than 3 kDa were obtained by filtration and analyzed directly by the MALDI ToF/ToF mass spectrometry. In the obtained spectrum eight signals from the candidate peptides were found, all with apparent mass less than 1500 Da (Figure 4).

All potential peptides were sequenced in LIFT mode. For parent ion 1386.728 *m*/*z*, 178 signals were obtained in the fragmentation spectrum; for 1188.5767 *m*/*z*, 140 signals; 1182.557 *m*/*z*, 114 signals; for 1176.600 *m*/*z*, 109 signals; 1166.597 *m*/*z*, 141 signals; 1144.620 *m*/*z*, 80 signals; and for 1072.570 *m*/*z*, 72 signals. Sequencing of parent ion 723.284 *m*/*z* failed. Sequences of four peptides obtained from SwissProt and NCBInr data bases are summarized in Table 2.

## 3. Discussion

Venoms produced by snakes consist of many components, of which proteins and peptides are the largest group. Many of these components have a synergistic effect, which ensures the quick effect of venom on prey. Victims hunted by a given snake species often belong to different taxonomic groups, and have developed a variety of safeguards against bites and its consequences. Therefore, the venom has agents acting “universally” on a wide range of organisms, as well as those whose activity is directed against a specific prey molecular targets [5].

For each agent in the human body associated with hemostasis, we may find a homolog, activator or an inhibitor in the venom [6] operating on the principle of protein-protein interactions or enzymatic proteolysis [7]. Hemotoxic venoms, like the one produced by a common European adder, affect blood vessel walls, platelets, coagulation, anticoagulation and fibrinolysis. It often happens that, in the venom of a single species we find components that are antagonists to hemostasis and even to individual factors associated with it [7].

Venom of *V. berus berus* consists of approximately 25 proteins and peptides with enzymatic activity [3,4], and the total venom composition only of some Russian specimens has been described so far [8]. Obtained gels of *V. berus berus* venom proteins contain even greater number of spots, but the identification using MALDI ToF/ToF showed that they belong only to 11 families. With high probability it can be assumed that the proteins in this venom are highly post-translationally modified, as it is shown clearly by visible spots trains in gels (Figure 1 and Figure 2). This phenomenon is characteristic for *Viperidae* family and was described already several times [9,10].

Our study indicates that the composition of the analyzed venom differs from that recently described in Latinović et al. [8]. However, direct comparison of obtained results from those two studies is not possible. Latinović et al. used normalized volumes of corresponding 1-D gel protein bands and areas of elution peaks from RP-HPLC for protein abundance estimation. On the other hand our study employs protein quantity estimation method based on spots volume from obtained 2-D gels after sample separation. Furthermore, it is not possible to incorporate our peptidome results in to the protein chart because of the method we used, as we have identified peptidome directly, without prior separation. In this case, MALDI ToF/ToF technique does not provide quantitative data, so we cannot determine the content of individual peptides in the venom. Keeping that in mind, direct comparison of the percent shares of major protein groups would indicate significantly larger amount of phospholipases A_2_ (59% vs. 10%), and much lower amount of serine proteinases (15% vs. 31%) in our results. The most prominent difference observed would be metalloproteinases share: 0.15% vs. 19%. Biological explanation for observed discrepancies would be snake gender, Latinović et al. does not declare it, and the snake habitat, in our case venom was obtained from the snakes captured in natural environment in Slovak Republic, or age of snakes and type of food. Influence of these factors was described before and could attribute to the observed differences [11].

*Vipera berus berus* venom has mainly hemotoxic activity and identified proteins clearly meet the criteria for a wide range of hemotoxins [3,4]. Hemotoxins can be classified based on their effects on the following groups [6,7]: (i) activating blood coagulation factors; (ii) anticoagulant agents; (iii) inhibitors and activators of platelets; (iv) agents affecting fibrinolysis; and (v) hemorrhagins. Proteins of the first group affect clotting factors or directly coagulate the fibrinogen (thrombin-like enzymes) (spots # 4 and 5). Most of them, however, cause the formation of fibrinopeptide A or B or, rarely both as it is in nature. Therefore, created clots are unstable and prone to endogenous or venom-induced fibrinolysis, which in turn leads to fibrinolysis syndrome. In the anti-coagulant agents group, we include those components of the venom, which inhibit tenas. There are mostly serine proteases (# 4 and 5), protein C activators and phospholipases A_2_ (# 9–11). Platelet activating proteins cause thrombocytopenia and are predominantly C-type lectin like proteins (# 8), and thrombin-like enzymes (# 4 and 5). In turn, deactivation of platelets and following hemorrhage is caused by disintegrins and snake venom metalloproteases (SVMPs) (# 2 and 7). The group of proteins responsible for fibrinolysis includes protein directly capable of disrupting the fibrin (# 4 and 5) or plasmin activators. The last group of proteins is the hemorrhagins–cytolysins damaging blood vessels and causing hemorrhages. They mostly include metalloproteases (# 2 and 7) [4,5]. We found all the above-described groups of proteins in the venom of *Vipera berus berus*. The specificity of these proteins clearly explains hemotoxic properties of this venom.

Most diverse group of proteins in European adder venom is the snaclec proteins belonging to C-type lectin like proteins. Most snaclec type proteins are non-enzymatic homodimers of a weight 26 and 28 kDa composed of subunits with weight about 13 and 18 kDa, and are responsible for the erythrocytes agglutination. They may also take the form of heterodimers or oligomers, and contribute to the activation or inhibition of human platelets [12]. Performed separation under denaturing conditions confirms their monomeric weight in the range of 15 to 25 kDa (Figure 1 and Figure 2). In the *V. berus berus* venom we identified eight homologues of these proteins from different species of *Viperidae* (# 8a–8g), constituting 5.5% of venom proteins and this is the first finding of these proteins in this species.

The largest group of proteins identified in the adder venom is a family of phospholipases A_2_ (PLA_2_) (60%). They are small enzymes with a mass of approximately 14 kDa corresponding to about 115–133 amino acid residues [13]. Depending on the amino acid composition they are divided into acidic, basic and neutral–all three groups we have identified in European adder (# 9–11). The snake venom’s phospholipases of group I and II are widely distributed in many snake species and are important neuro- and miotoxic agents, causing the immobilization of the prey. Often in the venom of snakes different types of phospholipases are present, which cause different pharmacological effects starting with blood coagulation disorders, through the inhibition of platelet aggregation, to blocking of neuromuscular signaling and skeletal muscle paralysis [14]. On gels (Figure 1 and Figure 2) the areas with these proteins appear in the 15 kDa region throughout the full used pH range, wherein the acidic, basic and neutral phospholipase were identified, confirming the literature data [13,14].

Serine proteases, also known as thrombin-like enzymes, are another big identified group (15%, # 4 and 5). They constitute a collection of enzymes that catalyze reactions involving a wide range of the blood coagulation cascade, fibrinolysis and platelet aggregation. Specific serine proteases catalyze usually only one or a few of the many reactions involved in blood coagulation. They have the ability to cut fibrinogen in the way similar to thrombin. This results in clot formation, acting not only through participation in the coagulation pathway, but also by direct platelet aggregation [15]. The molecular weight of these enzymes ranges from about 30 to 60 kDa. On the obtained polyacrylamide gels serine proteases are located in the area of pH 5–8 and the weight range of 35–50 kDa (Figure 1 and Figure 2). From the pharmacological point of view this group of proteins is very interesting and promising since they could be used in hyperfibrinogenemia treatment, an important risk factor for ischemic stroke and peripheral artery diseases [16].

In the venom of the *Viperidae* family all classes of SVMPs (snake venom metalloproteinases) are present, playing an important role in immobilizing prey by blocking the transmission of nerve signals, and tissue proteolysis in the initial digestion. They play an important role in the impairment of blood clotting causing immediate local bleeding and delayed internal bleeding [17,18,19]. As it is apparent from this research (# 2 and # 7) SVMPs type III containing metalloproteinase, disintegrin-like and a cysteine-rich domains are the largest class of metalloproteinases in *V. berus berus* venom. However, this class has a very small share in the venom proteome, less than 0.5%. Interestingly, earlier studies indicate a much larger share of this group of proteins in the venom of *V. berus berus* [8].

In the upper part of the gel a group of proteins identified as Angiotensin-like peptide 2 (# 1) was found. The molecular weight of this peptide is about 1 kDa, and the spot which contain proteins with this short sequence on the basis of which the identification was made, have weight almost one hundred times greater. This probably means that in the venom of European adder there is so far undescribed protein that is vasoactive, i.e., has a constricting or dilating effect on the caliber of blood vessels [20]. A second possibility is that the series of spots visible on gels (# 1) contains the precursors of bioactive peptides. Due to the fact that not all peptides were identified, there is a chance that considered venom includes peptides having angiotensin-like properties. However, this result requires more research as only one short peptide belonging to this protein was identified.

Besides basic phospholipase only two other proteins were assigned to the database entries as coming from the examined species. These include l-amino acid oxidases (# 3) and venom cysteine-rich proteins (# 6). l-amino acid oxidases are present in venoms of many snakes in large quantities and their toxicity is primarily due to oxidative stress induced by H_2_O_2_, which is produced in enzymatic reaction of oxidative deamination of l-amino acids [21]. These proteins have a very wide range of action from anticoagulation and inhibition of platelet aggregation to anti-viral and anti-bacterial properties [22,23,24,25]. In obtained gels l-amino acid oxidases appear in two areas (Figure 1 and Figure 2, # 3), and represent 9% of venom proteins. Spots in the upper molecular weight range correspond to the literature data [25] with weight of approximately 50 kDa. In turn, the spot in the lower molecular weight region of the gel match only the data from the UniProt (P0C2D7 (OXLA_VIPBB)) suggesting that this 88-amino acid protein has a mass of approximately 10 kDa. This observed difference may be due to the level of protein glycosylation, as in other species, or occurrence of isoforms of this enzyme [24,26].

The second protein derived from *V. berus berus* is a member of cysteine-rich venom protein CRISP (# 6). Proteins from this group shows wide variety of biological activities. There are many reports indicating that several venom-derived CRISPs could exhibit neurotoxicity due to their inhibitory effect on different types of ion channels [27,28]. Our experiment showed that this is the 4th largest group of proteins in the analyzed venom (6%).

The only protein that was not found as a result of our experiment was hyaluronidase. Hyaluronidase causes degradation of hyaluronic acid which increases the permeability of the tissue at the bite site, and hence the degree of absorption of the venom. Their action results in local swelling, blistering and necrosis [3]. Although numerous literature sources indicate that the venom has such properties, the factor responsible for them has not yet been found. Interestingly, despite many citations [25,29,30,31] only one work actually states the presence of agents capable of carrying out the depolymerization of hyaluronic acid [32].

Peptidome analysis showed the presence of 8 peptides in European adder venom, of which only four could be identified. All of them were identified as bradykinin-potentiating peptides. Hence, all of them could be inhibitors of angiotensin-converting enzyme and would enhance the action of bradykinin, and consequently act as hypotensive agents [33]. Potentially, they could act just like captopril, an oral medication based on the peptide from *Bothrops jararaca* venom. Interestingly, only one peptide detected in this experiment (1166.5968 *m*/*z*) was previously identified in other *Vipera* species [34], others are of Crotalinae origins (Table 2). Peptides contained in the venom have great pharmacological potential. They are poorly immunogenic and have evolutionary conserved tertiary structure, obtained mostly by disulfide bonds and posttranslational modifications [35]. The most common of these modifications is pyroglutamate residue at the *N*-terminus [33,34] observed in two peptides of *V. berus berus* (Table 2).

It is necessary to note that the meaningful identification for 2 out of 4 isolated peptides have been obtained only when the posttranslational modification of deamination NQ was included in the Mascot search parameters. Unfortunately, it is not possible to determine with our current experimental setup if such a modification is of a natural origin or it is an artifact.

Presented results clearly show that the venom of European adder has mainly hemotoxic effect, as inferred from a large number of proteins from the family of metalloproteinases, serine proteases, L-amino acid oxidases or C-type lectin-like proteins. They exhibit toxic effects on the vascular system, causing abnormal blood clotting. Furthermore, l-amino acid oxidases act by causing neuromuscular blockade, and lead to the destruction of the cells by breaking cell membrane during its depolarization. In the venom of this snake we also observe a few proteins responsible for neurotoxicity, these are a cysteine-rich proteins responsible for the blockade of nerve conduction and phospholipases A_2_ possessing both neuro-, myo-, cyto- and hemotoxic properties. Literature data indicate that the effects of European adder venom is based mainly on the disorder of homeostasis and the impairment of blood clotting process, as shown by the presented results. This work describes for the first time the peptidome of *V. berus berus*. Identified peptides potentially have blood pressure lowering properties and may present a valuable target for further pharmacological investigations.

## 4. Materials and Methods

The individual European adders (*Vipera berus berus*), both females and males were captured in Slovak Republic at altitudes ranging from 650 to 750 meters above sea level. Collected venom from multiple snakes was pooled together and immediately frozen at −20 °C.

In order to separate the peptides, venom was filtered on a centrifugal filters with a 3 kDa cutoff in accordance with the manufacturer's instructions (VWR 82031-344).

For comparative study of protein composition the two-dimensional electrophoresis and protein identification based on MALDI ToF/ToF mass spectrometry were chosen. For the peptide analysis direct mass spectrometry approach was used. Protein concentration was determined using the 2-D Quant Kit (GE Healthcare, Little Chalfont, UK) with bovine serum albumin as a standard. Aliquots of 405 μg proteins were mixed with respective thiourea rehydration solutions containing different IPG buffers: pH range 3–10 and 5–8 (7 M urea, 2 M thiourea, 2% Nonidet P-40, 0.5% IPG buffer, 0.002% bromophenol blue, and 18 mM DTT) to a final volume of 300 μL and used for 2-DE [36]. Rehydratation and isoelectrofocusing were performed on 17 cm ReadyStrip IPG Strips (Bio-Rad, Hercules, CA, USA) pH 5–8 and 3–10 at 50 μA per strip at 20 °C, according the following program: 12 h of active rehydration at 50 V and focusing: linear gradient 250 V–20 min, linear gradient 10,000 V–3 h, and rapid gradient 10,000 V–40,000 VHours, using PROTEAN IEF Cell device (Bio-Rad). Before the second dimension the strips were equilibrated for 15 min in SDS equilibration buffer solutions (6 M urea, 75 mM TRIS-HCl pH 8.8, 29.3% glycerol, 2% SDS, 0.002% bromophenol blue) first containing 1% DTT and second 2.5% iodoacetamide instead of DTT. In the second dimension under reducing and denaturing condition (SDS-PAGE) the proteins were separated using 13% polyacrylamide gels (1.5 mm × 255 mm × 196 mm) with Roti^®^-Mark PRESTAINED molecular weight marker (Roth, Karlsruhe, Germany) as a standard. The electrophoresis was performed in Ettan Dalt Six (GE Healthcare) for 5.5 h, first at 4 Wηgel^−1^ for 30 min and then at 17 Wηgel^−1^. Following electrophoresis the gels were stained with colloidal Coomassie Brilliant Blue G-250 [37]. Gels were scanned with ImageScanner III (GE Healthcare) and processed by LabScan 6.0 (GE Healthcare). All gels were prepared in three technical replicates.

Percentage of proteins from different groups has been estimated in Image Master 2D Platinum software using %Vol (a ratio of the volume of a particular spot to the total volume of all spots present in the gel). The calculation has been done for each gel separately. The final result is an average of the spots %Vol determined from all gels including technical repeats as well as the different isoelectrofocusing separation range.

All the spots present on gels were excised from gels and digested using Sequencing Grade Modified Trypsin (Promega, Madison, WI, USA) according to protocol previously described [38]. Peptides derived from the filtration and those derived from the proteins digestion were mixed with the matrix α-Cyano-4-hydroxycinnamic acid in 1:1 ratio.

Peptide masses were measured using a MALDI-ToF/ToF MS (Autoflex Speed, Bruker Daltonics, Germany). The sample ionization was performed with laser beam at 337 nm. The analyzer worked in the reflective mode and positive ions were recorded in the mass range between 700 and 3500 Da. Mass calibration was performed after every four samples using standards in the range of analytes (Peptide Calibration Standards I, Bruker Daltonics, Billerica, MA, USA). The obtained peptide mass fingerprint data were exported to the Mascot software for MSDB (Model System Database, London, UK) or SwissProt database search (www.matrixscience.com). The following search parameters were applied: mass tolerance was set to 0.2 Da, one incomplete cleavage was allowed, alkylation of cysteine by carbamidomethylation as fixed, and oxidation of methionine as variable modification were set.

Particular peptides selected from mass spectrum were sequenced by laser-induced dissociation (LID) using LIFT ion source and tandem mass spectrum were analyzed as described above. The search parameters for MS/MS data were the same as those applied for MALDI-ToF analyses with one exception: mass tolerance was set to 0.4 Da for MS mode and 0.2 Da for MS/MS mode. For peptidome analysis no fixed modifications were marked but additional variable modifications have been selected instead: N-terminal glutamate to pyroglutamate conversion and deamidation on asparagine.

The mass spectrometry proteomics data have been deposited to the ProteomeXchange Consortium via the PRIDE [39] partner repository with the dataset identifier PXD004958.

## Figures and Tables

**Table 1:** Composition of *V. berus berus* venom proteins.

Spot No. ^#^	Identified Protein ^&^	Accession *	Organism ^¥^	Mass (kDa) ^£^	S ^±^	Peptide Sequence ^≠^
**1**	Angiotensin- like peptide 2	ANGT2_BOTJA	*Bothrops jararaca*	1.04	44	DRVYIHPF (1046.5816)
**2**	H3 metalloproteinase precursor 1	VM1H3_DEIAC	*Vipera ammotydes ammotydes*	70.7	48	NPCQIYYTPR (1311.7225)
**3**	l-amino acid oxidase	OXLA_BOTPC	*Bothrops pictus*	56.7	102	SAGQLYEESLR (1252.6349)
		OXLA_BOTCO	*Bothrops cotiara*	1.8	49	NPLEECFR (1252.6575)
		OXLA_MACLB	*Macrovipera lebetina*	12.5	58	PMF SC 40.2%
		OXLA_BOTJR	*Bothrops jararacussu*	56.7	59	FWEDDGIHGGK (1260.6088)
		OXLA_VIPBB	*Vipera berus berus*	10.3	68	ADDICNPLEECFR (1493.7661)
		OXLA_BOTJA	*Bothrops jararacussu*	56.5	50	HIDDIFAYEK (1137.5991)
		OXLA_BOTPC	*Bothrops pictus*	56.7	101	SAGQLYEESLR (1252.7064)
		OXLA_BOTIN	*Bothrops insularis*	5.3	37	ADDKNPLEECFR (1493.7653)
**4a**	Serine protease VLSp-3	VSP3_MACLB	*Macrovipera lebetina*	29	25	TSTHIAPLSLPSSPPSVGSVCR (2250.4505)
**4b**	Snake venom serine protease nikobin	VSP_VIPNI	*Vipera nikolskii*	28.8	64	PMF SC 25.7%
					53	CQGVHPELPAK (1235.6438)
					55	VVCAGIWQGGK (1174.6110)
					96	VILPDVPHCANIEIIK (1831.0636)
					36	EYTMWDK (972.4713)
**5**	Beta- fibrinogenase brevinase	VSPB_GLOBL	*Gloydius blomhoffi*	26.3	56	VIGGDECNINEHR (1512.7806)
		VSPBF_MACLB	*Macrovipera lebetina*	28.2	26	FFCLSSK (888.4284)
	Thrombin-like enzyme crotalase	VSPCR_CROAD	*Crotalus adamanteus*	30.1	36	WDKDIMLIR (1189.6561)
	Venom serine proteinase-like protein 2	VSP2_MACLB	*Macrovipera lebetina*	29.5	70	IMGWGTITTTK (1208.6420)
					134	TLCAGILQGGIDSCK (1592.7767)
**6**	Cysteine rich venom protein	CRVP_VIPBE	*Vipera berus*	27.4	73	MEWYPEAAANAER (1537.6894)
					53	PMF SC 24.7%
					74	PMF SC 36.4%
					51	KPEIQNEIIDLHNSLR (1919.0970)
**7**	Snake venom metalloproteinase VMP1	VM1V1_AGKPL	*Agkistrodon piscivorus leucostoma*	47.1	58	NPQCILNKPLR (1352.7017)
	Zinc metalloproteinase/disintegrin	VM2L2_MACLB	*Macrovipera lebetina*	47.1	58	NPQCILNQPL (1352.7017)
**8a**	Snaclec rhinocetin submit beta	SLRB_BITRH	*Bitis rhinoceros*	18.7	59	TTDNQWLR (1033.6040)
**8b**	Snaclec A14	SLAE_MACLB	*Macrovipera lebetina*	18	39	TSADYVWIGLWNQR (1708.9104)
**8c**	Snaclec B6	SLB6_MACLB	*Macrovipera lebetina*	15.3	43	ANLVWIGLR (1041.6803)
**8d**	Snaclec echicetin subunit alpha	SLA_ECHCA	*Echis carinatus*	16.1	37	TWDEAEKFCNK (1427.6446)
**8e**	Snaclec 1	SL1_ECHCS	*Echis carinatus sochureki*	17.1	35	GSHLVSLHNIAEADFVVK (1936.0465)
**8f**	Snaclec rhodocetin subunit alpha	SLEA_CALRH	*Calloselasma rhodostoma*	15.9	47	TWEEAER (920.46147)
**8f**	Snaclec A13	SLAD_MACLB	*Macrovipera lebetina*	15.6	26	DQDCLPGWSFYEGHCYK (2161.9310)
**8g**	Snaclec jerdonibitin submit alpha	SLA_PROJR	*Protobothrops jerdoni*	18.1	45	TWEDAER (906.4700)
**9**	Acidic phospholipase ammodytin I1	PA2A1_VIPAA	*Vipera ammodytes ammodytes*	16.2	56	PMF SC 30.4%
	Acidic phospholipase A2 PLA-1	PA2A1_ERIMA	*Eristicophis macmahoni*	14.3	58	PMF SC 36.4%
	Acidic phospholipase A2 PL1	PA2A1_VIPRE	*Viprea renardi*	16.2	72	CCFVHDCCYGR (1533.4506)
**10**	Neutral phospholipase A2 ammodytoxin I2	PA2N_DABRR	*Vipera ammodytes ammodytes*	16.1	56	PMF SC 30%
**11**	Basic phospholipase A2 ammodytoxin C	PA2BA_VIPAA	*Vipera ammodytes ammodytes*	15.4	28	AAAICFR (808.4193)
	Basic phospholipase A2 Pla2Vb	PA2B_VIPBB	*Vipera berus berus*	15.7	48	HICECDR (989.4389)
	Basic phospholipase A2 vurtoxin	PA2B_VIPRE	*Vipera renardi*	16.4	31	YYPDFLCK (1105.5024)
	Basic phospholipase A2	PA2B3_DABRR	*Daboia russelii*	14.4	142	CCFVHDCCYGNLPDCNPK (2315.8464)

^#^ Spot numbering was the same as in Figure 1 and Figure 2; ^&^ Protein name in database; * Database accession number of homologous proteins; ^¥^ Organism from which protein originates; ^£^ The mass of molecule; ^±^ Protein identification was performed using the Mascot search with probability based Mowse score. Ions score was −10 × log(P), where P was the probability that the observed match was a random event. ^≠^ Peptide sequence derived from LIFT analysis. Identification of proteins by MS/MS method was conducted by comparing obtained sequences with sequences from database. In brackets mass of precursor ion. In the case of PMF, identification SC amino acid sequence coverage for the identified proteins. In the PMF identification case, the highest score and SC are shown.

**Table 2:** Composition of the peptidome of *V. berus berus* venom.

Parent Ion *m*/*z*	Identified Peptide ^&^	Accession *	Organism ^¥^	Peptide Sequence ^≠^	Mass (Da) ^£^	S ^±^
1072.5704	Bradykinin potentiating peptide D	gi 229282	*Gloydius blomhoffii*	EGLPPRPIPP	1072	14
1144.6198	Bradykinin-potentiating peptide S1,2,4	BPP4_BOTIN	*Bothrops insularis*	QGGPPRPQIPP + Deamidated (NQ)	1143	31
1166.5968	Bradykinin-potentiating peptide 11a	BP11A_CROVV	*Crotalus viridis viridis*	QGPSPRHPIPP + Gln→pyro-Glu (N-term Q); Deamidated (NQ)	1182	5
1182.5725	Bradykinin-potentiating peptide 10d	BPPAD_BOTCO	*Bothrops cotiara*	QNWPHPPMPP + Gln→pyro-Glu (N-term Q)	1200	9

^&^ Peptide name in database; * Database accession number of homologous peptide; ^¥^ Organism from which peptide originates; ^£^ The mass of molecule; ^±^ Protein identification was performed using the Mascot search with probability based Mowse score. Ions score was −10 × log(P), where P was the probability that the observed match was a random event; ^≠^ Peptide sequence derived from LIFT analysis.

## Supplementary Materials

Supplementary materials can be accessed at: http://www.mdpi.com/1420-3049/21/10/1398/s1.
