# Supplementary material for: Proteome and Peptidome of Vipera berus berus Venom
Source: Molecules. 2016 Oct 19;21(10):1398. doi: 10.3390/molecules21101398 (PMC6274168; doi:10.3390/molecules21101398)
Supplement: Supplementary file 1 [file molecules-21-01398-s001.pdf]

Supplementary Materials: Proteome and Peptidome of *Vipera berus berus* Venom

Aleksandra Bocian, Małgorzata Urbanik, Konrad Hus, Andrzej Łyskowski, Vladimír Petrilla, Zuzana Andrejčáková, Monika Petrillová and Jaroslav Legath

Selected MS and MS/MS Annotated Spectra used for Protein and Peptide Identification

Area 1. Annotated MS/MS spectrum of ion 1046.574 *m*/*z*.

Spectrum Analysis Report

Sequence Name:		Parentmass:	1046.574	Mass Error:	0.032	MH+ (mono):	1046.542
MH+ (avg):	1047.189	Threshold (a.i.):	0.000	Tolerance (Da):	0.700	Number of Peaks:	62
Above Threshold:	62	Assigned Peaks:	35	Not assigned Peaks:	27		

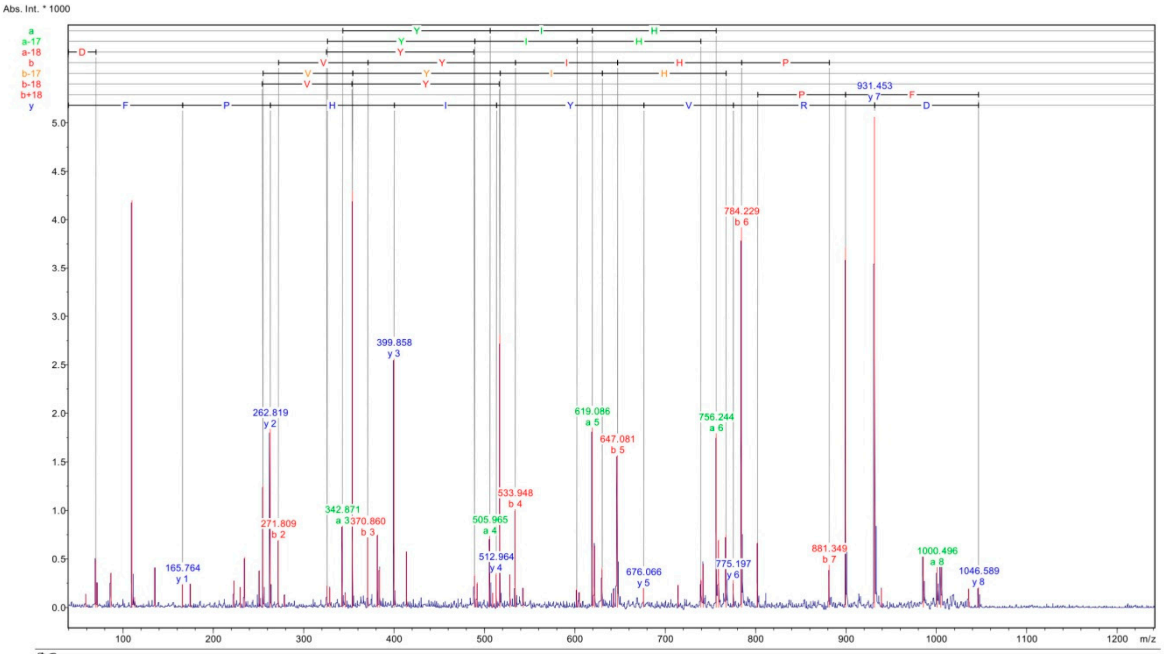

BioTools Version 3.2 Copyright © 1999-2008 Bruker Daltonik GmbH Page -1-

Spectrum Analysis Report

Display Parameter:

Parentmass:	1046.574	Mass Error:	0.032	MH+ (mono):	1046.542	MH+ (avg):	1047.189
Threshold (a.i.):	0.000	Tolerance (Da):	0.700	Number of Peaks:	62	Above Threshold:	62
Assigned Peaks:	35	Not assigned Peaks:	27				

Peaklist:

Peak	Mass	Intensity	Peak	Mass	Intensity	Peak	Mass	Intensity	Peak	Mass	Intensity	Peak	Mass	Intensity
1	58.841	132.159	2	69.865	498.245	3	71.869	256.271	4	86.850	360.129	5	109.818	4202.408
6	112.824	108.794	7	135.809	411.972	8	165.764	240.044	9	174.821	244.590	10	222.849	276.943
11	229.802	212.178	12	234.807	520.571	13	250.827	384.116	14	254.793	1256.001	15	262.819	1839.622
16	271.809	696.719	17	279.016	127.240	18	325.866	228.955	19	328.915	201.144	20	342.871	843.072
21	346.113	142.528	22	353.853	4297.906	23	370.860	720.696	24	381.850	752.246	25	383.953	400.974
26	399.858	2561.915	27	413.884	577.251	28	488.958	343.859	29	491.980	260.729	30	505.965	738.269
31	509.054	147.996	32	512.964	356.662	33	516.943	2808.390	34	528.238	341.272	35	533.948	1013.366
36	542.879	201.688	37	602.070	197.489	38	605.023	158.256	39	619.086	1853.371	40	622.068	637.158
41	630.068	412.111	42	643.303	172.112	43	647.081	1568.024	44	676.066	199.785	45	714.197	236.801
46	739.214	298.317	47	742.097	451.583	48	756.244	1794.773	49	758.970	700.023	50	767.182	754.570
51	775.197	282.431	52	784.229	3921.564	53	802.274	670.928	54	881.349	441.579	55	899.393	3717.282
56	931.453	5215.078	57	939.297	190.885	58	985.525	525.154	59	1000.496	416.983	60	1004.629	553.061
61	1035.696	191.849	62	1046.589	210.618									

Calculated Masses:
DRVYIHPF

N-Term.	Ion	a	a-17	a-18	b	b-17	b-18	b+18	c	i	x	y	z	C-Term.	Ion
1	D	88.039	71.013	**70.029**	116.034	99.008	98.024	134.045	133.061	88.039	192.066	**166.086**	149.060	8	F
2	R	244.140	227.114	226.130	**272.135**	**255.109**	**254.125**	290.146	289.162	129.113	289.118	**263.139**	246.112	7	P
3	V	**343.209**	**326.182**	**325.198**	**371.204**	**354.177**	**353.193**	389.214	388.230	**72.081**	426.177	**400.198**	383.171	6	H
4	Y	**506.272**	**489.246**	**488.262**	**534.267**	**517.241**	**516.257**	552.278	551.294	**136.076**	539.261	**513.282**	496.255	5	I
5	I	**619.356**	**602.330**	601.346	**647.351**	**630.325**	629.341	665.362	664.378	86.096	702.325	**676.345**	659.319	4	Y
6	H	**756.415**	**739.389**	738.405	**784.410**	**767.383**	766.399	**802.421**	801.437	**110.071**	801.393	**775.414**	**758.387**	3	V
7	P	853.468	836.441	835.457	**881.463**	864.436	863.452	**899.473**	898.489	**70.065**	957.494	**931.515**	914.488	2	R
8	F	**1000.536**	983.510	982.526	1028.531	1011.505	1010.521	**1046.542**	1045.558	120.081	1072.521	**1046.542**	1029.515	1	D

BioTools Version 3.2 Copyright © 1999-2008 Bruker Daltonik GmbH Page -2-

Area 1. Annotated MS/MS spectrum of ion 1046.574 *m*/*z*.

Spectrum Analysis Report

Sequence Name:		Parentmass:	1046.574	Mass Error:	0.032	MH+ (mono):	1046.542
MH+ (avg):	1047.189	Threshold (a.i.):	0.000	Tolerance (Da):	0.700	Number of Peaks:	62
Above Threshold:	62	Assigned Peaks:	35	Not assigned Peaks:	27		

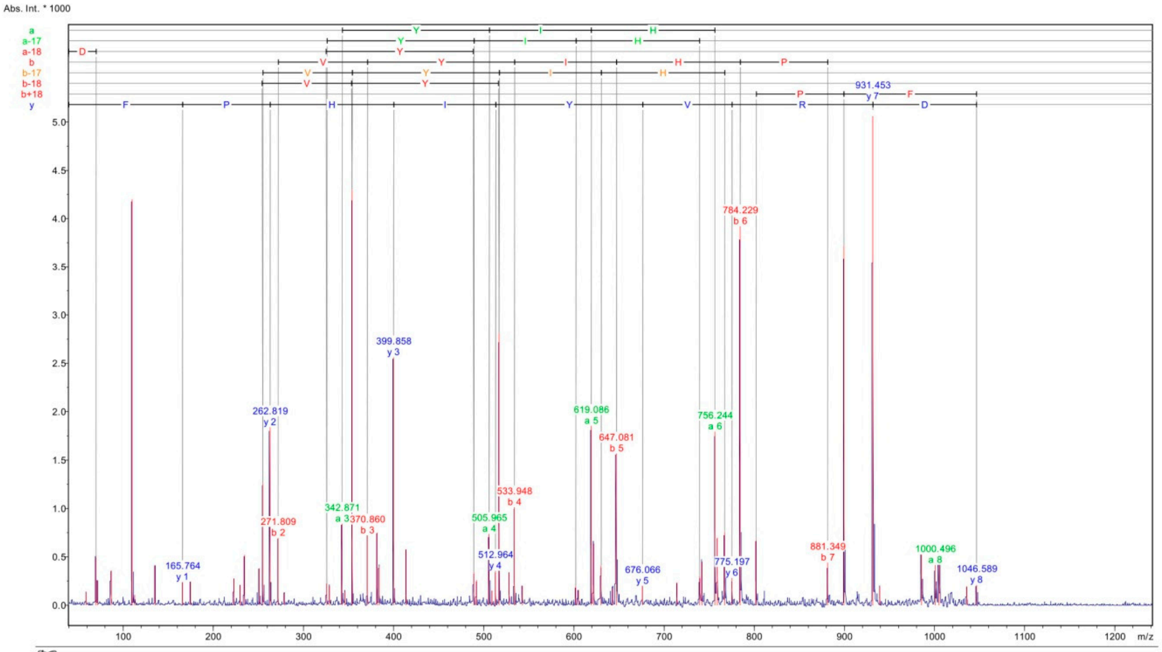

BioTools Version 3.2 Copyright © 1999-2008 Bruker Daltonik GmbH Page -1-

Spectrum Analysis Report

Display Parameter:

Parentmass:	1046.574	Mass Error:	0.032	MH+ (mono):	1046.542	MH+ (avg):	1047.189
Threshold (a.i.):	0.000	Tolerance (Da):	0.700	Number of Peaks:	62	Above Threshold:	62
Assigned Peaks:	35	Not assigned Peaks:	27				

Peaklist:

Peak	Mass	Intensity	Peak	Mass	Intensity	Peak	Mass	Intensity	Peak	Mass	Intensity	Peak	Mass	Intensity
1	58.841	132.159	2	69.865	498.245	3	71.869	256.271	4	86.850	360.129	5	109.818	4202.408
6	112.824	108.794	7	135.809	411.972	8	165.764	240.044	9	174.821	244.590	10	222.849	276.943
11	229.802	212.178	12	234.807	520.571	13	250.827	384.116	14	254.793	1256.001	15	262.819	1839.622
16	271.809	696.719	17	279.016	127.240	18	325.866	228.955	19	328.915	201.144	20	342.871	843.072
21	346.113	142.528	22	353.853	4297.906	23	370.860	720.696	24	381.850	752.246	25	383.953	400.974
26	399.858	2561.915	27	413.884	577.251	28	488.958	343.859	29	491.980	260.729	30	505.965	738.269
31	509.054	147.996	32	512.964	356.662	33	516.943	2808.390	34	528.238	341.272	35	533.948	1013.366
36	542.879	201.688	37	602.070	197.489	38	605.023	158.256	39	619.086	1853.371	40	622.068	637.158
41	630.068	412.111	42	643.303	172.112	43	647.081	1568.024	44	676.066	199.785	45	714.197	236.801
46	739.214	298.317	47	742.097	451.583	48	756.244	1794.773	49	758.970	700.023	50	767.182	754.570
51	775.197	282.431	52	784.229	3921.564	53	802.274	670.928	54	881.349	441.579	55	899.393	3717.282
56	931.453	5215.078	57	939.297	190.885	58	985.525	525.154	59	1000.496	416.983	60	1004.629	553.061
61	1035.696	191.849	62	1046.589	210.618									

Calculated Masses:
DRVYIHPF

N-Term.	Ion	a	a-17	a-18	b	b-17	b-18	b+18	c	i	x	y	z	C-Term.	Ion
1	D	88.039	71.013	**70.029**	116.034	99.008	98.024	134.045	133.061	88.039	192.066	**166.086**	149.060	8	F
2	R	244.140	227.114	226.130	**272.135**	**255.109**	**254.125**	290.146	289.162	129.113	289.118	**263.139**	246.112	7	P
3	V	**343.209**	**326.182**	**325.198**	**371.204**	**354.177**	**353.193**	389.214	388.230	**72.081**	426.177	**400.198**	383.171	6	H
4	Y	**506.272**	**489.246**	**488.262**	**534.267**	**517.241**	**516.257**	552.278	551.294	**136.076**	539.261	**513.282**	496.255	5	I
5	I	**619.356**	**602.330**	601.346	**647.351**	**630.325**	629.341	665.362	664.378	86.096	702.325	**676.345**	659.319	4	Y
6	H	**756.415**	**739.389**	738.405	**784.410**	**767.383**	766.399	**802.421**	801.437	**110.071**	801.393	**775.414**	**758.387**	3	V
7	P	853.468	836.441	835.457	**881.463**	864.436	863.452	**899.473**	898.489	**70.065**	957.494	**931.515**	914.488	2	R
8	F	**1000.536**	983.510	982.526	1028.531	1011.505	1010.521	**1046.542**	1045.558	120.081	1072.521	**1046.542**	1029.515	1	D

BioTools Version 3.2 Copyright © 1999-2008 Bruker Daltonik GmbH Page -2-

Area 2. Annotated MS/MS spectrum of ion 1311.723 *m*/*z*.

Spectrum Analysis Report

Sequence Name:		Parentmass:	1311.723	Mass Error:	0.107	MH+ (mono):	1311.616
MH+ (avg):	1312.477	Threshold (a.i.):	0.000	Tolerance (Da):	0.500	Number of Peaks:	33
Above Threshold:	33	Assigned Peaks:	22	Not assigned Peaks:	11		

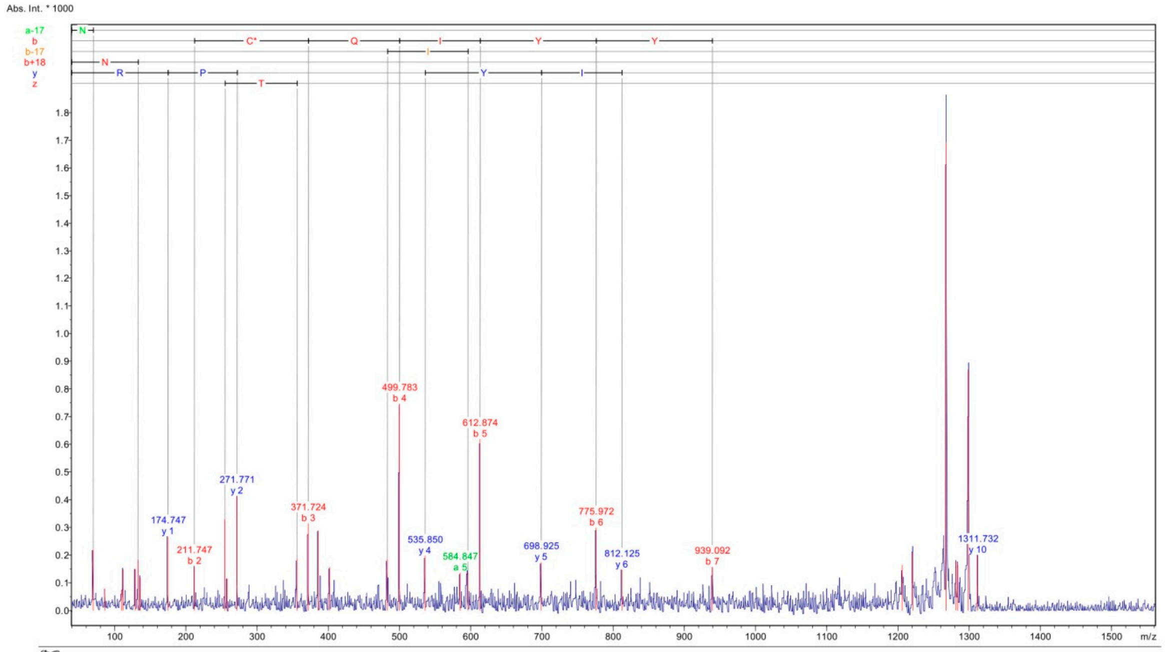

Spectrum Analysis Report

Display Parameter:

Parentmass:	1311.723	Mass Error:	0.107	MH+ (mono):	1311.616	MH+ (avg):	1312.477
Threshold (a.i.):	0.000	Tolerance (Da):	0.500	Number of Peaks:	33	Above Threshold:	33
Assigned Peaks:	22	Not assigned Peaks:	11				

Peaklist:

Peak	Mass	Intensity	Peak	Mass	Intensity	Peak	Mass	Intensity	Peak	Mass	Intensity	Peak	Mass	Intensity
1	69.828	221.927	2	85.806	79.441	3	109.711	75.361	4	111.768	149.492	5	128.783	150.630
6	132.713	179.870	7	135.737	119.601	8	174.747	267.735	9	211.747	160.403	10	254.741	331.830
11	257.697	115.933	12	271.771	414.388	13	355.763	188.594	14	371.724	314.998	15	385.741	289.759
16	401.811	157.259	17	482.749	186.716	18	499.783	747.129	19	535.850	198.157	20	584.847	137.353
21	595.894	138.116	22	612.874	619.606	23	698.925	175.176	24	775.972	299.355	25	812.125	147.909
26	939.092	158.080	27	1205.820	164.722	28	1220.642	208.929	29	1267.723	1694.006	30	1281.290	178.477
31	1283.834	165.243	32	1298.428	870.607	33	1311.732	200.803						

Calculated Masses:
NPCQIYYTPR 3: Carbamidomethyl (C)

N-Term.	Ion	a	a-17	a-18	b	b-17	b-18	b+18	c	i	x	y	z	C-Term.	Ion
1	N	87.055	**70.029**	69.045	115.050	98.024	97.040	**133.061**	132.077	87.055	201.098	**175.119**	158.092	10	R
2	P	184.108	167.082	166.097	**212.103**	195.076	194.092	230.114	229.130	**70.065**	298.151	**272.172**	**255.145**	9	P
3	C*	344.139	327.112	326.128	**372.134**	355.107	354.123	390.144	389.160	**133.043**	399.199	373.219	**356.193**	8	T
4	Q	472.197	455.171	454.187	**500.192**	**483.166**	482.182	518.203	517.219	101.071	562.262	**536.283**	519.256	7	Y
5	I	**585.281**	568.255	567.271	**613.276**	**596.250**	595.266	631.287	630.303	**86.096**	725.325	**699.346**	682.320	6	Y
6	Y	748.345	731.318	730.334	**776.340**	759.313	758.329	794.350	793.366	**136.076**	838.409	**812.430**	795.404	5	I
7	Y	911.408	894.381	893.397	**939.403**	922.376	921.392	957.413	956.429	**136.076**	966.468	940.489	923.462	4	Q
8	T	1012.456	995.429	994.445	1040.451	1023.424	1022.440	1058.461	1057.477	74.060	1126.499	1100.519	1083.493	3	C*
9	P	1109.508	1092.482	1091.498	1137.503	1120.477	1119.493	1155.514	1154.530	**70.065**	1223.551	1197.572	1180.546	2	P
10	R	1265.610	1248.583	1247.599	1293.604	1276.578	1275.594	**1311.615**	1310.631	**129.113**	1337.594	**1311.615**	1294.588	1	N

Area 3. Annotated MS/MS spectrum of ion 1252.585 *m*/*z*.

Spectrum Analysis Report

Sequence Name:		Parentmass:	1252.585	Mass Error:	-0.032	MH+ (mono):	1252.617
MH+ (avg):	1253.341	Threshold (a.i.):	0.000	Tolerance (Da):	0.700	Number of Peaks:	64
Above Threshold:	64	Assigned Peaks:	36	Not assigned Peaks:	28		

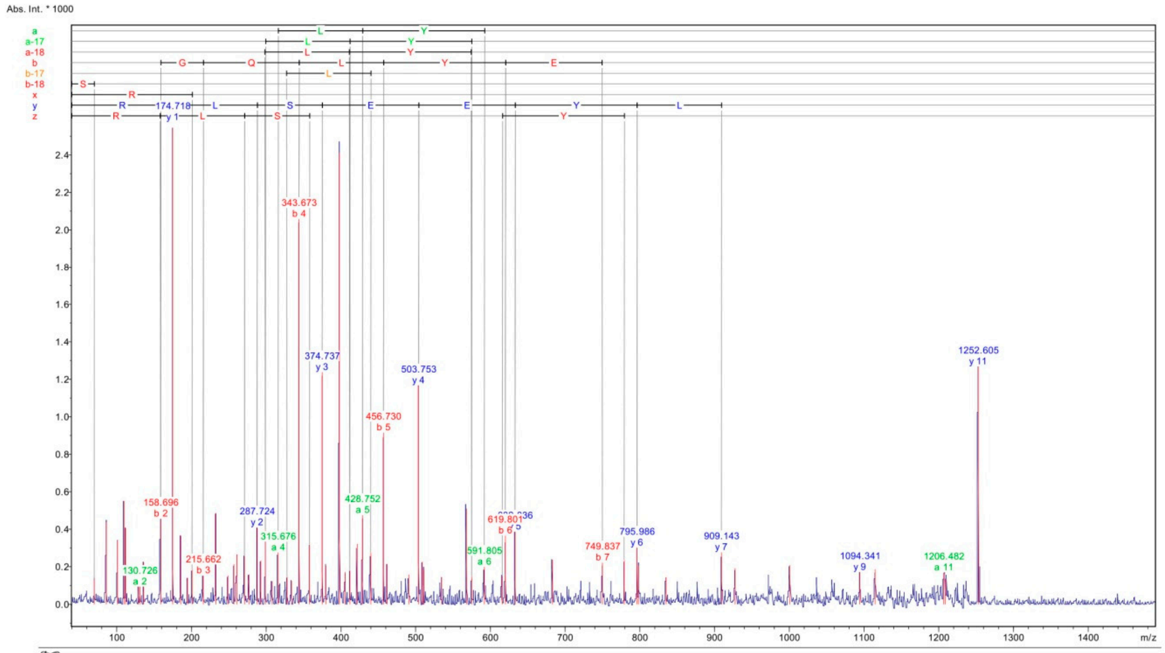

BioTools Version 3.2 Copyright © 1999-2008 Bruker Daltonik GmbH Page -1-

Spectrum Analysis Report

Display Parameter:

Parentmass:	1252.585	Mass Error:	-0.032	MH+ (mono):	1252.617	MH+ (avg):	1253.341
Threshold (a.i.):	0.000	Tolerance (Da):	0.700	Number of Peaks:	64	Above Threshold:	64
Assigned Peaks:	36	Not assigned Peaks:	28				

Peaklist:

Peak	Mass	Intensity	Peak	Mass	Intensity	Peak	Mass	Intensity	Peak	Mass	Intensity	Peak	Mass	Intensity
1	69.793	135.494	2	85.803	438.962	3	100.752	338.743	4	109.733	551.573	5	111.746	406.652
6	128.762	146.589	7	130.726	93.754	8	135.722	225.852	9	158.696	456.966	10	174.718	2575.821
11	185.678	366.654	12	194.656	141.682	13	200.702	184.725	14	215.662	153.937	15	232.689	487.120
16	248.676	154.192	17	256.686	214.025	18	260.701	267.699	19	270.728	257.021	20	276.706	160.398
21	287.724	409.669	22	292.652	234.192	23	298.708	398.223	24	307.695	123.763	25	315.676	276.524
26	327.670	145.902	27	333.704	124.781	28	343.673	2058.408	29	357.737	317.547	30	374.737	1238.367
31	379.718	207.077	32	397.713	2414.096	33	405.704	173.086	34	411.765	180.553	35	421.679	322.944
36	428.752	475.771	37	439.745	274.262	38	456.730	915.905	39	461.749	215.206	40	490.681	159.437
41	503.753	1167.013	42	508.712	227.421	43	510.749	186.775	44	534.723	142.266	45	567.820	511.059
46	574.803	149.092	47	591.805	199.786	48	615.795	152.153	49	619.801	366.533	50	632.836	387.671
51	682.923	238.330	52	749.837	222.777	53	778.901	236.575	54	795.986	302.093	55	798.106	207.831
56	834.961	144.367	57	909.143	277.815	58	927.356	192.851	59	1000.338	209.194	60	1094.341	171.618
61	1114.632	187.700	62	1206.482	169.273	63	1208.635	137.338	64	1252.605	1268.629			

Calculated Masses:
SAGQLYEESLR

N-Term.	Ion	a	a-17	a-18	b	b-17	b-18	b+18	c	i	x	y	z	C-Term.	Ion
1	S	60.044	43.018	42.034	88.039	71.013	**70.029**	106.050	105.066	60.044	**201.098**	**175.119**	**158.092**	11	R
2	A	**131.082**	114.055	113.071	**159.076**	142.050	141.066	177.087	176.103	44.049	314.182	**288.203**	**271.176**	10	L
3	G	188.103	171.076	170.092	**216.098**	199.071	198.087	234.108	**233.124**	30.034	401.214	**375.235**	**358.208**	9	S
4	Q	**316.162**	**299.135**	**298.151**	**344.156**	**327.130**	326.146	362.167	361.183	**101.071**	530.257	**504.278**	487.251	8	E
5	L	**429.246**	**412.219**	**411.235**	**457.241**	**440.214**	**439.230**	475.251	474.267	**86.096**	659.299	**633.320**	**616.294**	7	E
6	Y	**592.309**	**575.282**	**574.298**	**620.304**	603.277	602.293	638.314	637.330	**136.076**	822.363	**796.384**	**779.357**	6	Y
7	E	721.352	704.325	703.341	**749.346**	732.320	731.336	767.357	766.373	102.055	935.447	**909.468**	892.441	5	L
8	E	850.394	833.368	832.384	878.389	861.362	860.378	896.400	895.416	102.055	1063.505	1037.526	1020.500	4	Q
9	S	937.426	920.400	919.416	965.421	948.395	947.411	983.432	982.448	60.044	1120.527	**1094.548**	1077.521	3	G
10	L	1050.510	1033.484	1032.500	1078.505	1061.479	1060.495	1096.516	1095.532	**86.096**	1191.564	1165.585	1148.558	2	A
11	R	**1206.611**	1189.585	1188.601	1234.606	1217.580	1216.596	**1252.617**	1251.633	**129.113**	1278.596	**1252.617**	1235.590	1	S

BioTools Version 3.2 Copyright © 1999-2008 Bruker Daltonik GmbH Page -2-

Area 3. Annotated MS/MS spectrum of ion 1064.508 *m*/*z*.

Spectrum Analysis Report

Sequence Name:
MH+ (avg): 1065.184
Above Threshold: 83
Parentmass: 1064.508
Threshold (a.i.): 0.000
Assigned Peaks: 32
Mass Error: 0.024
Tolerance (Da): 0.700
Not assigned Peaks: 51
MH+ (mono): 1064.484
Number of Peaks: 83

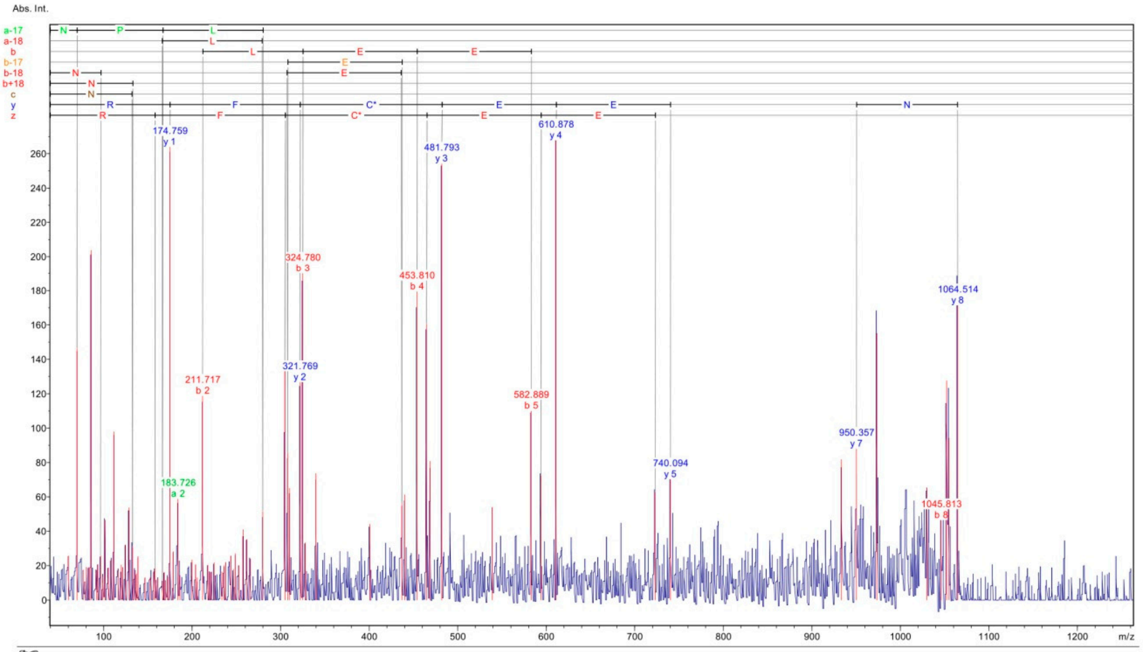

Copyright © 1999-2008 Bruker Daltonik GmbH

Spectrum Analysis Report

Display Parameter:

Parentmass: 1064.508
Threshold (a.i.): 0.000
Assigned Peaks: 32
Mass Error: 0.024
Tolerance (Da): 0.700
Not assigned Peaks: 51
MH+ (mono): 1064.484
Number of Peaks: 83
MH+ (avg): 1065.184
Above Threshold: 83

Peaklist:

Peak	Mass	Intensity	Peak	Mass	Intensity	Peak	Mass	Intensity	Peak	Mass	Intensity	Peak	Mass	Intensity
1	60.244	25.829	2	69.831	146.353	3	76.808	17.785	4	80.478	19.302	5	83.242	19.259
6	85.843	203.847	7	92.069	17.536	8	93.560	20.800	9	96.481	25.940	10	99.095	13.705
11	101.773	46.706	12	105.260	18.978	13	108.103	23.720	14	111.774	98.051	15	116.275	17.429
16	119.758	20.607	17	121.422	19.005	18	124.745	32.325	19	128.781	53.917	20	132.729	31.032
21	135.637	17.423	22	136.896	15.450	23	138.787	25.555	24	146.706	13.223	25	152.650	13.314
26	156.336	16.739	27	157.744	18.637	28	161.242	12.627	29	166.403	11.393	30	168.359	16.344
31	169.859	11.686	32	172.148	13.538	33	174.759	264.002	34	178.747	28.279	35	183.726	58.898
36	186.071	14.586	37	199.770	23.572	38	203.943	20.876	39	205.622	13.133	40	211.717	118.753
41	217.731	20.555	42	219.858	16.293	43	222.124	16.535	44	224.745	22.043	45	233.432	16.590
46	235.771	20.259	47	237.850	22.170	48	243.706	25.953	49	246.428	18.179	50	248.732	27.237
51	252.610	20.279	52	257.655	41.150	53	261.741	35.557	54	265.778	23.887	55	279.713	51.611
56	304.734	138.202	57	307.737	85.650	58	309.800	65.216	59	321.769	126.729	60	324.780	190.096
61	327.729	33.494	62	339.747	73.822	63	400.719	44.322	64	436.799	55.904	65	439.775	61.426
66	453.810	179.437	67	464.758	160.465	68	468.796	80.861	69	481.793	254.271	70	538.869	54.192
71	582.889	110.102	72	593.890	72.511	73	610.878	267.784	74	722.907	62.891	75	740.094	70.207
76	933.296	81.916	77	950.357	87.996	78	973.299	155.280	79	1029.648	65.588	80	1045.813	46.454
81	1052.186	127.770	82	1054.765	94.357	83	1064.514	171.377						

Calculated Masses:
NPLEECFR 6: Carbamidomethyl (C)

N-Term.	Ion	a	a-17	a-18	b	b-17	b-18	b+18	c	i	x	y	z	C-Term.	Ion
1	N	87.055	**70.029**	69.045	115.050	98.024	**97.040**	**133.061**	**132.077**	87.055	201.098	**175.119**	**158.092**	8	R
2	P	**184.108**	**167.082**	**166.097**	**212.103**	195.076	194.092	230.114	229.130	**70.065**	348.167	**322.187**	**305.161**	7	F
3	L	297.192	**280.166**	**279.182**	**325.187**	**308.160**	**307.176**	343.198	342.214	**86.096**	508.197	**482.218**	**465.191**	6	C*
4	E	426.235	409.208	408.224	**454.230**	**437.203**	**436.219**	472.240	471.256	**102.055**	637.240	**611.261**	**594.234**	5	E
5	E	555.277	**538.251**	537.267	**583.272**	566.246	565.262	601.283	600.299	**102.055**	766.282	**740.303**	**723.277**	4	E
6	C*	715.308	698.281	697.297	743.303	726.276	725.292	761.313	760.329	**133.043**	879.367	853.387	836.361	3	L
7	F	862.376	845.350	844.366	890.371	873.345	872.361	908.382	907.398	**120.081**	976.419	**950.440**	**933.413**	2	P
8	R	1018.477	1001.451	1000.467	**1046.472**	**1029.446**	1028.462	**1064.483**	1063.499	**129.113**	1090.462	**1064.483**	1047.456	1	N

Copyright © 1999-2008 Bruker Daltonik GmbH

Area 3. Annotated MS/MS spectrum of ion 1137.599 *m*/*z*.

Spectrum Analysis Report

Sequence Name:		Parentmass:	1137.599	Mass Error:	0.077	MH+ (mono):	1137.522
MH+ (avg):	1138.209	Threshold (a.i.):	0.000	Tolerance (Da):	0.700	Number of Peaks:	71
Above Threshold:	71	Assigned Peaks:	28	Not assigned Peaks:	43		

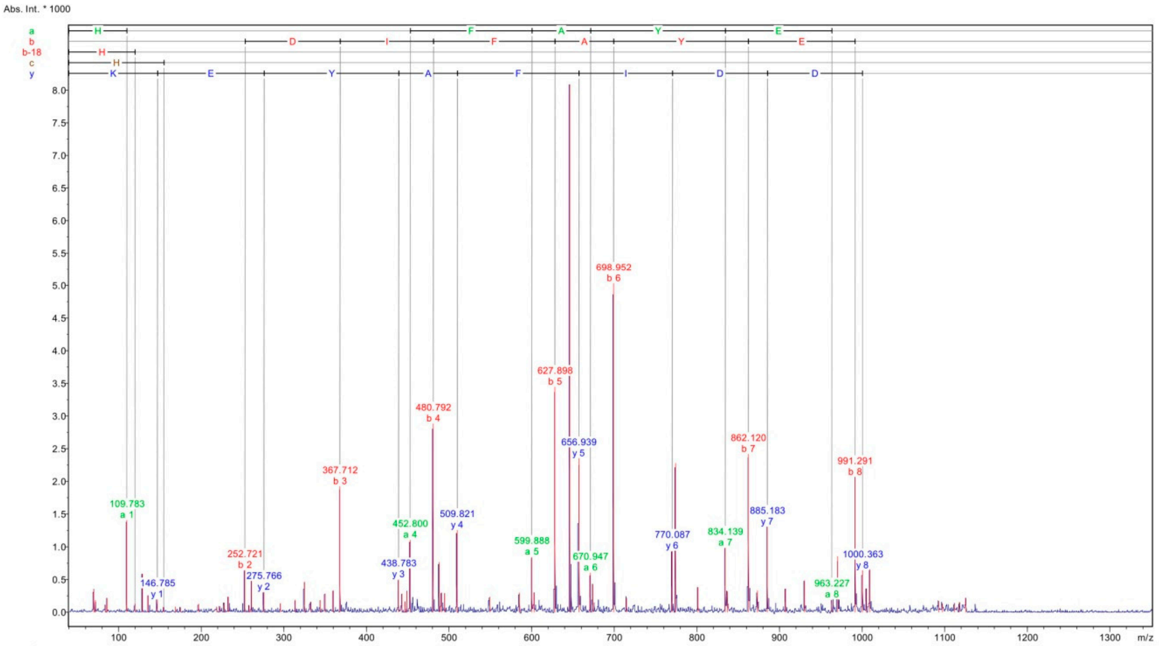

BioTools Version 3.2 Copyright © 1999-2008 Bruker Daltonik GmbH Page -1-

Spectrum Analysis Report

Display Parameter:

Parentmass:	1137.599	Mass Error:	0.077	MH+ (mono):	1137.522	MH+ (avg):	1138.209
Threshold (a.i.):	0.000	Tolerance (Da):	0.700	Number of Peaks:	71	Above Threshold:	71
Assigned Peaks:	28	Not assigned Peaks:	43				

Peaklist:

Peak	Mass	Intensity	Peak	Mass	Intensity	Peak	Mass	Intensity	Peak	Mass	Intensity	Peak	Mass	Intensity
1	69.816	343.836	2	71.826	189.351	3	83.828	113.505	4	85.829	216.619	5	109.783	1410.460
6	119.786	127.164	7	128.796	588.410	8	135.764	256.266	9	146.785	196.634	10	154.743	80.691
11	168.819	82.002	12	174.764	77.173	13	196.786	131.240	14	218.746	83.513	15	222.754	93.353
16	227.822	147.788	17	232.785	238.738	18	252.721	645.376	19	257.774	107.832	20	260.760	643.451
21	275.766	311.945	22	295.766	137.382	23	313.777	193.026	24	324.846	465.919	25	332.819	161.661
26	343.741	187.793	27	349.788	284.947	28	359.808	334.088	29	367.712	1927.375	30	438.783	501.639
31	442.828	285.223	32	446.796	167.186	33	448.823	318.046	34	452.800	1107.542	35	480.792	2886.692
36	487.939	769.126	37	490.794	299.923	38	494.721	291.238	39	509.821	1258.117	40	548.937	236.978
41	584.968	301.358	42	599.888	846.409	43	602.952	307.497	44	627.898	3449.228	45	646.015	8251.069
46	656.939	2354.501	47	670.947	604.765	48	673.901	426.309	49	698.952	5032.405	50	714.705	219.482
51	770.087	938.651	52	774.153	2281.561	53	801.163	384.730	54	834.139	986.599	55	836.802	323.125
56	862.120	2420.739	57	873.230	335.233	58	885.183	1308.410	59	907.301	361.966	60	930.213	489.515
61	963.227	193.910	62	970.304	857.672	63	991.291	2068.049	64	1000.363	639.914	65	1005.259	363.334
66	1009.321	659.582	67	1092.536	172.839	68	1097.097	136.056	69	1111.640	131.236	70	1117.472	157.670
71	1125.611	210.636												

Calculated Masses:
HDDIFAYEK

N-Term.	Ion	a	a-17	a-18	b	b-17	b-18	b+18	c	i	x	y	z	C-Term.	Ion
1	H	**110.071**	93.045	92.061	138.066	121.040	**120.056**	156.077	**155.093**	**110.071**	173.092	**147.113**	130.086	9	K
2	D	225.098	208.072	207.088	**253.093**	236.067	235.083	271.104	270.120	88.039	302.135	**276.155**	259.129	8	E
3	D	340.125	323.099	322.115	**368.120**	351.094	**350.110**	386.131	385.147	88.039	465.198	**439.219**	422.192	7	Y
4	I	**453.209**	436.183	435.199	**481.204**	464.178	463.194	499.215	498.231	**86.096**	536.235	**510.256**	493.229	6	A
5	F	**600.278**	583.251	582.267	**628.273**	611.246	610.262	**646.283**	645.299	**120.081**	683.304	**657.324**	640.298	5	F
6	A	**671.315**	654.288	653.304	**699.310**	682.283	681.299	717.320	716.336	44.049	796.388	**770.408**	753.382	4	I
7	Y	**834.378**	817.352	816.368	**862.373**	845.346	844.362	880.384	879.400	**136.076**	911.415	**885.435**	868.409	3	D
8	E	**963.421**	946.394	945.410	**991.416**	974.389	973.405	**1009.426**	1008.442	102.055	1026.441	**1000.462**	983.436	2	D
9	K	1091.516	1074.489	1073.505	1119.511	1102.484	1101.500	1137.521	1136.537	101.107	1163.500	1137.521	1120.495	1	H

BioTools Version 3.2 Copyright © 1999-2008 Bruker Daltonik GmbH Page -2-

Area 3. Annotated MS/MS spectrum of ion 1493.766 *m*/*z*.

Spectrum Analysis Report

Sequence Name:		Parentmass:	1493.766	Mass Error:	0.097	MH+ (mono):	1493.669
MH+ (avg):	1494.610	Threshold (a.i.):	0.000	Tolerance (Da):	0.700	Number of Peaks:	71
Above Threshold:	71	Assigned Peaks:	31	Not assigned Peaks:	40		

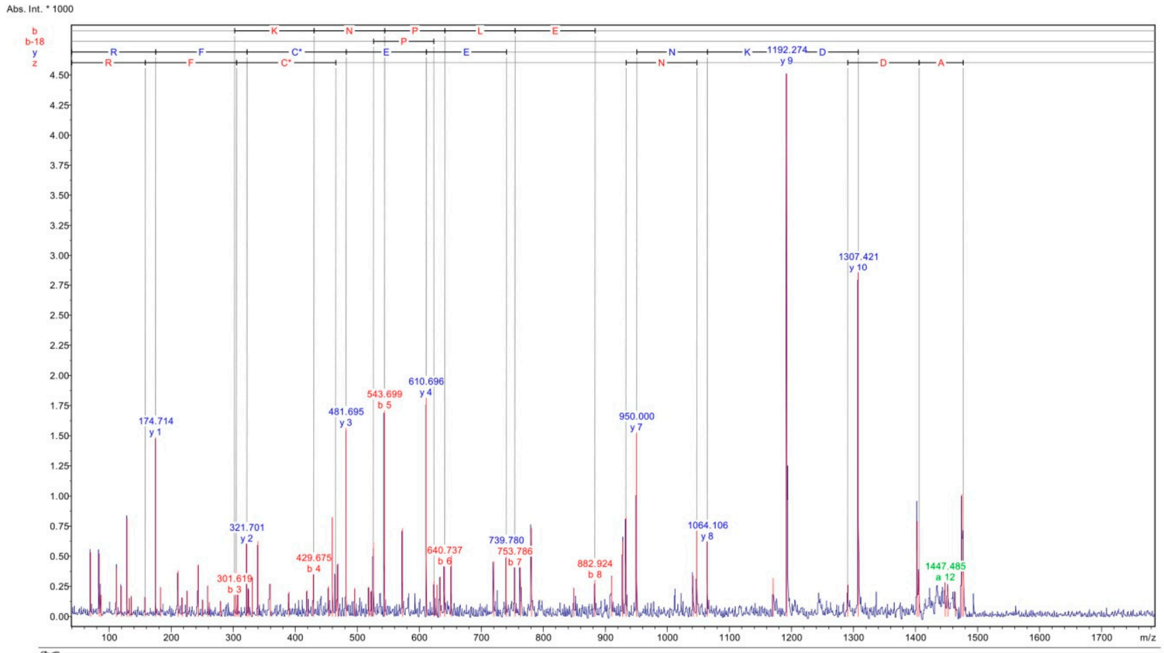

BioTools Version 3.2 Copyright © 1999-2008 Bruker Daltonik GmbH Page -1-

Spectrum Analysis Report

Display Parameter:

Parentmass:	1493.766	Mass Error:	0.097	MH+ (mono):	1493.669	MH+ (avg):	1494.610
Threshold (a.i.):	0.000	Tolerance (Da):	0.700	Number of Peaks:	71	Above Threshold:	71
Assigned Peaks:	31	Not assigned Peaks:	40				

Peaklist:

Peak	Mass	Intensity	Peak	Mass	Intensity	Peak	Mass	Intensity	Peak	Mass	Intensity	Peak	Mass	Intensity
1	69.788	533.000	2	83.780	522.383	3	86.759	179.815	4	111.736	404.479	5	119.744	252.616
6	128.742	814.149	7	132.718	147.264	8	135.673	158.545	9	157.700	157.081	10	174.714	1487.122
11	182.743	245.937	12	210.719	383.533	13	217.698	158.171	14	225.698	215.399	15	243.679	433.203
16	250.686	137.867	17	258.613	259.311	18	279.669	122.667	19	301.619	179.997	20	304.683	379.614
21	307.668	225.272	22	321.701	603.582	23	324.723	223.328	24	330.746	321.783	25	339.707	625.370
26	359.608	270.042	27	389.695	206.157	28	418.643	220.812	29	429.675	352.456	30	453.716	251.210
31	459.744	828.781	32	464.678	349.082	33	468.696	441.304	34	481.695	1565.615	35	495.677	238.176
36	518.698	248.093	37	522.642	214.592	38	525.687	616.799	39	543.699	1710.060	40	572.807	731.432
41	610.696	1816.242	42	623.742	299.958	43	628.730	265.302	44	633.667	331.401	45	640.737	414.690
46	651.638	551.091	47	719.840	457.348	48	739.780	497.298	49	753.786	406.528	50	762.730	503.373
51	780.748	735.241	52	848.942	238.046	53	882.924	303.847	54	909.848	324.258	55	927.860	628.996
56	932.935	832.334	57	950.000	1527.053	58	1041.118	341.143	59	1047.057	738.955	60	1064.106	622.161
61	1170.363	319.204	62	1192.274	4574.991	63	1290.335	265.616	64	1307.421	2854.830	65	1402.620	791.963
66	1405.563	368.177	67	1447.485	282.744	68	1452.004	245.644	69	1463.647	204.078	70	1474.443	1016.820
71	1477.058	407.507												

Calculated Masses:
ADDKNPLEECFR 10: Carbamidomethyl (C)

N-Term.	Ion	a	a-17	a-18	b	b-17	b-18	b+18	c	i	x	y	z	C-Term.	Ion
1	A	44.049	27.023	26.039	72.044	55.018	54.034	90.055	89.071	44.049	201.098	**175.119**	**158.092**	12	R
2	D	159.076	142.050	141.066	187.071	170.045	169.061	205.082	204.098	88.039	348.167	**322.187**	**305.161**	11	F
3	D	274.103	257.077	256.093	**302.098**	285.072	284.088	320.109	319.125	88.039	508.197	**482.218**	**465.191**	10	C*
4	K	402.198	385.172	384.188	**430.193**	413.167	412.183	448.204	447.220	101.107	637.240	**611.261**	594.234	9	E
5	N	516.241	499.215	498.231	**544.236**	527.210	**526.226**	562.247	561.263	**87.055**	766.282	**740.303**	723.277	8	E
6	P	613.294	596.267	595.283	**641.289**	**624.262**	**623.278**	659.299	658.315	**70.065**	879.367	853.387	836.361	7	L
7	L	726.378	709.352	708.368	**754.373**	737.346	736.362	772.384	771.400	**86.096**	976.419	**950.440**	**933.413**	6	P
8	E	855.421	838.394	837.410	**883.416**	866.389	865.405	901.426	900.442	102.055	1090.462	**1064.483**	**1047.456**	5	N
9	E	984.463	967.437	966.453	1012.458	995.432	994.448	1030.469	1029.485	102.055	1218.557	**1192.578**	1175.551	4	K
10	C*	1144.494	1127.467	1126.483	1172.489	1155.462	1154.478	1190.499	1189.515	**133.043**	1333.584	**1307.605**	**1290.578**	3	D
11	F	1291.562	1274.536	1273.552	1319.557	1302.531	1301.547	1337.568	1336.584	**120.081**	1448.611	1422.632	**1405.605**	2	D
12	R	**1447.663**	1430.637	1429.653	1475.658	1458.632	1457.648	1493.669	1492.685	**129.113**	1519.648	1493.669	**1476.642**	1	A

BioTools Version 3.2 Copyright © 1999-2008 Bruker Daltonik GmbH Page -2-

Area 3. Annotated MS/MS spectrum of ion 1260.623 *m*/*z*.

Spectrum Analysis Report

Sequence Name: Parentmass: 1260.623 Mass Error: 0.058 MH+ (mono): 1260.565
MH+ (avg): 1261.322 Threshold (a.i.): 0.000 Tolerance (Da): 0.700 Number of Peaks: 39
Above Threshold: 39 Assigned Peaks: 28 Not assigned Peaks: 11

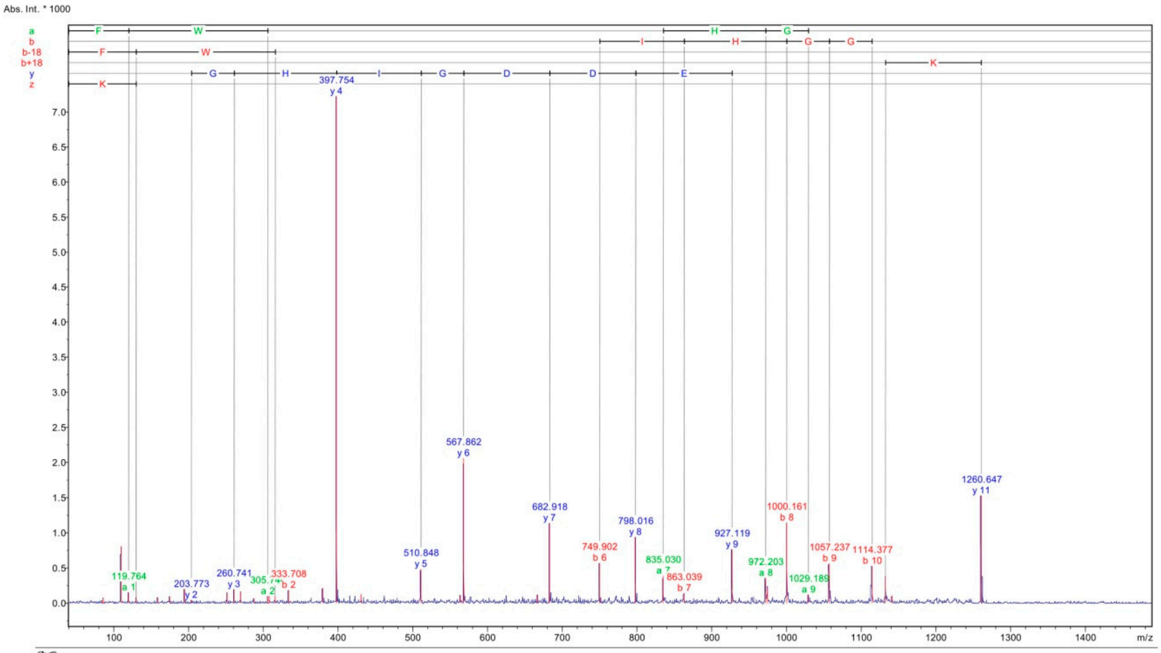

 Copyright © 1999-2008 Bruker Daltonik GmbH

Spectrum Analysis Report

Display Parameter:

Parentmass: 1260.623 Mass Error: 0.058 MH+ (mono): 1260.565 MH+ (avg): 1261.322
Threshold (a.i.): 0.000 Tolerance (Da): 0.700 Number of Peaks: 39 Above Threshold: 39
Assigned Peaks: 28 Not assigned Peaks: 11

Peaklist:

Peak	Mass	Intensity	Peak	Mass	Intensity	Peak	Mass	Intensity	Peak	Mass	Intensity	Peak	Mass	Intensity
1	83.763	41.274	2	85.811	78.589	3	109.761	809.766	4	119.764	153.613	5	129.722	78.582
6	158.744	83.771	7	174.703	97.548	8	194.716	242.710	9	203.773	44.434	10	251.679	152.532
11	260.741	197.775	12	269.701	167.081	13	287.677	67.138	14	305.744	97.987	15	307.727	139.552
16	315.684	94.405	17	333.708	187.865	18	379.733	216.777	19	397.754	7226.385	20	430.733	127.606
21	510.848	484.471	22	563.788	114.094	23	567.862	2063.657	24	666.890	121.582	25	682.918	1142.320
26	749.902	573.819	27	798.016	940.423	28	835.030	389.136	29	863.039	142.839	30	927.119	765.625
31	972.203	358.851	32	974.751	206.653	33	1000.161	1145.100	34	1029.189	120.803	35	1057.237	567.054
36	1114.377	529.165	37	1132.371	380.499	38	1141.247	108.700	39	1260.647	1530.961			

Calculated Masses:
FWEDDGIHGGK

N-Term.	Ion	a	a-17	a-18	b	b-17	b-18	b+18	c	i	x	y	z	C-Term.	Ion
1	F	**120.081**	103.054	102.070	148.076	131.049	**130.065**	166.086	165.102	**120.081**	173.092	147.113	**130.086**	11	K
2	W	**306.160**	289.134	**288.150**	**334.155**	317.128	**316.144**	352.166	351.182	**159.092**	230.114	**204.134**	187.108	10	G
3	E	435.203	418.176	417.192	463.198	446.171	445.187	481.208	480.224	102.055	**287.135**	**261.156**	244.129	9	G
4	D	550.230	533.203	532.219	578.225	561.198	560.214	596.235	595.251	88.039	424.194	**398.215**	381.188	8	H
5	D	665.257	648.230	647.246	693.251	676.225	675.241	711.262	710.278	88.039	537.278	**511.299**	494.272	7	I
6	G	722.278	705.251	704.267	**750.273**	733.246	732.262	768.284	767.299	30.034	594.299	**568.320**	551.294	6	G
7	I	**835.362**	818.336	817.352	**863.357**	846.330	845.346	881.368	880.384	**86.096**	709.326	**683.347**	**666.321**	5	D
8	H	**972.421**	955.394	954.410	**1000.416**	983.389	982.405	1018.426	1017.442	**110.071**	824.353	**798.374**	781.348	4	D
9	G	**1029.442**	1012.416	1011.432	**1057.437**	1040.411	1039.427	1075.448	1074.464	30.034	953.396	**927.417**	910.390	3	E
10	G	1086.464	1069.437	1068.453	**1114.459**	1097.432	1096.448	**1132.469**	1131.485	30.034	1139.475	1113.496	1096.469	2	W
11	K	1214.559	1197.532	1196.548	1242.554	1225.527	1224.543	**1260.564**	1259.580	101.107	1286.544	**1260.564**	1243.538	1	F

 Copyright © 1999-2008 Bruker Daltonik GmbH

Area 3. Annotated MS spectrum 1.

Spectrum Analysis Report

Sequence Name:	L-amino-acid oxidase (Fragments) OS=Macrovipera lebetina PE=1 SV=2 OXLA_MACLB					Parentmass:	
Mass Error:		MH+ (mono):	1.008	MH+ (avg):	1.008	Threshold (a.i.):	0.000
Tolerance (Da):	0.500	Number of Peaks:	67	Above Threshold:		Assigned Peaks:	
Not assigned Peaks:							

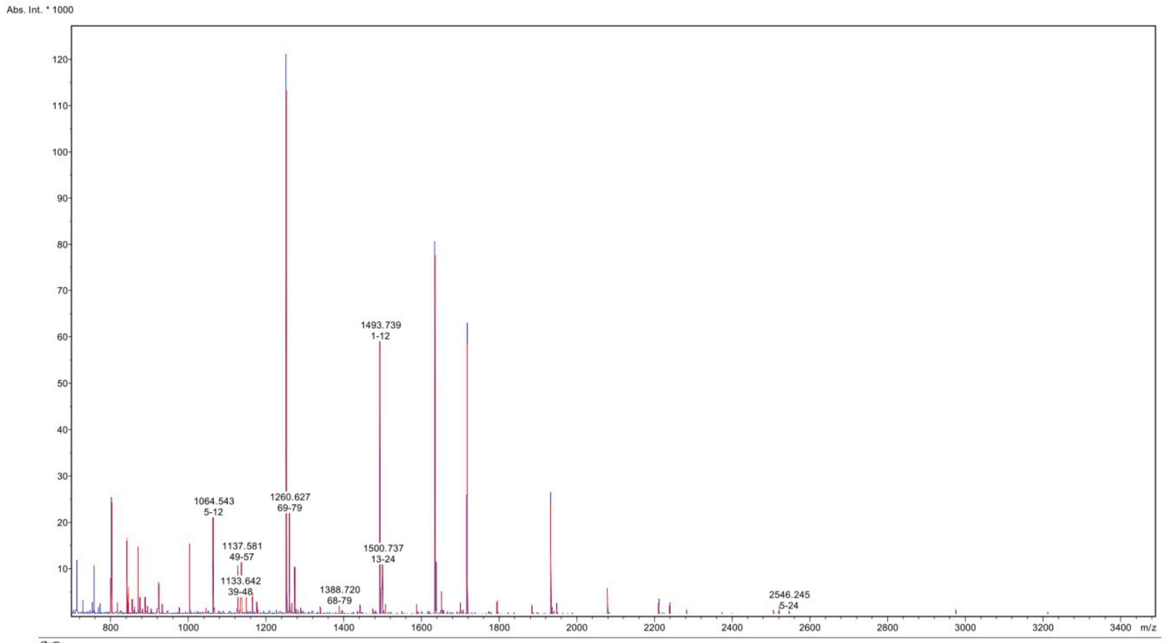

BioTools Version 3.2 Copyright © 1999-2008 Bruker Daltonik GmbH Page -1-

Spectrum Analysis Report

Sequence data:

L-amino-acid oxidase (Fragments) OS=Macrovipera lebetina PE=1 SV=2 OXLA_MACLB

Intensity Coverage:	21.5 % (131184 cnts)	Sequence Coverage MS:	51.4%
Sequence Coverage MS/MS:	0.0%	pI (isoelectric point):	4.4

10 20 30 40 50 60 70 80 90 100 110
ADDKNPLEEC FREDDYEEFL EIAKNGLEGW YANLGPMRYP VKPSEEGKHD DIFAYEKFDE IVGGMDKKFW EDDGIHGGKE TFCYSPMIQK PYQFQHFSEA LTAPVGR

Display Parameter:

Sequence Name:	L-amino-acid oxidase (Fragments) OS=Macrovipera lebetina PE=1 SV=2 OXLA_MACLB					MH+ (mono):	1.008
MH+ (avg):	1.008	Threshold (a.i.):	0.000	Tolerance (Da):	0.500	Number of Peaks:	67

Peaklist:

Peak	Mass	Intensity	Peak	Mass	Intensity	Peak	Mass	Intensity	Peak	Mass	Intensity	Peak	Mass	Intensity
1	801.544	8134.699	2	803.477	24198.183	3	817.521	2588.171	4	842.562	16642.805	5	844.535	4548.600
6	845.570	6055.236	7	856.578	3534.049	8	861.549	1715.237	9	870.597	14836.669	10	876.492	3965.253
11	882.622	1181.345	12	889.600	3842.561	13	895.526	1919.414	14	905.572	1229.809	15	919.540	1147.030
16	924.576	6592.769	17	933.630	2404.758	18	977.659	1574.993	19	1003.602	15066.075	20	1045.620	1523.005
21	1064.543	21060.262	22	1127.640	9495.382	23	1133.642	3880.082	24	1137.581	11422.342	25	1149.583	6874.682
26	1165.761	4850.660	27	1177.647	2751.347	28	1179.667	1220.383	29	1252.681	113235.987	30	1260.627	21980.865
31	1266.694	2382.128	32	1274.651	10294.676	33	1282.612	925.895	34	1290.638	1327.183	35	1340.685	1537.844
36	1388.720	1981.743	37	1396.814	878.548	38	1442.764	2144.462	39	1475.749	1213.376	40	1482.748	829.603
41	1493.739	59010.808	42	1500.737	10912.461	43	1507.750	1951.552	44	1587.834	2225.570	45	1635.834	77655.189
46	1651.825	4658.694	47	1657.845	980.369	48	1700.883	2457.596	49	1707.851	980.602	50	1717.908	58538.424
51	1774.922	757.596	52	1794.888	2825.612	53	1884.983	1898.947	54	1932.977	24085.264	55	1941.005	663.420
56	1948.969	2278.209	57	2079.079	4356.947	58	2211.188	2466.645	59	2221.206	423.256	60	2239.218	2044.002
61	2283.258	843.187	62	2373.263	402.427	63	2506.283	679.562	64	2521.318	1335.951	65	2546.245	935.866
66	2975.413	620.349	67	3212.642	295.400									

BioTools Version 3.2 Copyright © 1999-2008 Bruker Daltonik GmbH Page -2-

Area 3. Annotated MS spectrum 2.

Spectrum Analysis Report

Sequence Name:	L-amino-acid oxidase (Fragments) OS=Macrovipera lebetina PE=1 SV=2 OXLA_MACLB			Parentmass:	
Mass Error:	MH+ (mono): 1.008	MH+ (avg):	1.008	Threshold (a.i.):	0.000
Tolerance (Da): 0.500	Number of Peaks: 66	Above Threshold:		Assigned Peaks:	
Not assigned Peaks:					

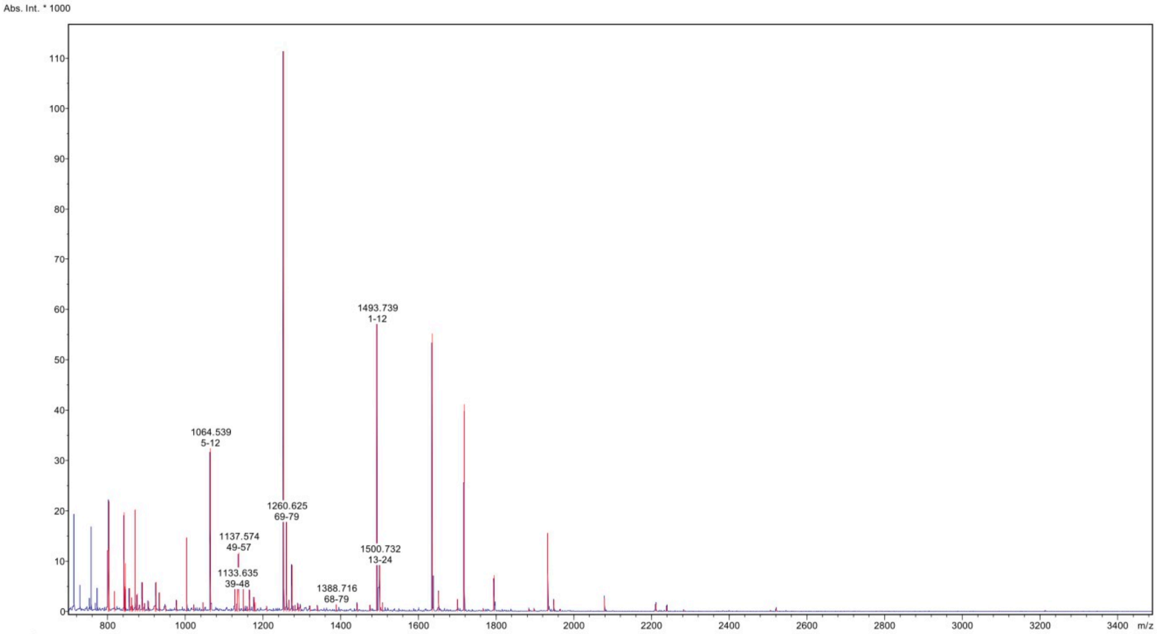

BioTools Version 3.2 Copyright © 1999-2008 Bruker Daltonik GmbH Page -1-

Spectrum Analysis Report

Sequence data:

L-amino-acid oxidase (Fragments) OS=Macrovipera lebetina PE=1 SV=2 OXLA_MACLB

Intensity Coverage:	23.1 % (134117 cnts)	Sequence Coverage MS:	51.4%
Sequence Coverage MS/MS:	0.0%	pI (isoelectric point):	4.4

```
       10         20         30         40         50         60         70         80         90         100        110
ADDKNPLEEC FREDDYEEFL EIAKNGLEGW YANLGPMRYP VKPSEEGKHD DIFAYEKFDE IVGGMDKKFW EDDGIHGGKE TFCYSPMIQK PYQFQHFSEA LTAPVGR
```

Display Parameter:

Sequence Name:	L-amino-acid oxidase (Fragments) OS=Macrovipera lebetina PE=1 SV=2 OXLA_MACLB			MH+ (mono):	1.008
MH+ (avg):	1.008	Threshold (a.i.): 0.000	Tolerance (Da): 0.500	Number of Peaks:	66

Peaklist:

Peak	Mass	Intensity	Peak	Mass	Intensity	Peak	Mass	Intensity	Peak	Mass	Intensity	Peak	Mass	Intensity
1	801.539	12485.222	2	803.469	21929.975	3	817.517	4051.369	4	842.557	19704.285	5	844.522	5000.734
6	845.566	9625.394	7	856.572	4644.810	8	861.541	2813.328	9	864.542	1327.577	10	870.594	20315.984
11	876.490	3599.005	12	882.611	1308.186	13	889.601	5801.339	14	895.521	1628.658	15	905.568	2017.214
16	922.570	1153.900	17	924.571	5911.921	18	933.628	3829.197	19	949.597	1224.110	20	977.656	2239.856
21	1003.599	14751.471	22	1021.685	1323.635	23	1045.616	1803.749	24	1064.539	32428.353	25	1127.635	8299.172
26	1133.635	4451.103	27	1137.574	11719.988	28	1149.577	4776.456	29	1159.568	985.724	30	1165.756	4264.772
31	1177.641	2892.538	32	1179.655	1865.111	33	1209.678	983.587	34	1252.676	114487.874	35	1260.625	17794.880
36	1266.689	2196.371	37	1274.646	9249.011	38	1282.609	1237.011	39	1290.634	1577.765	40	1296.629	1263.277
41	1320.650	1199.349	42	1340.678	1103.543	43	1388.716	1416.180	44	1442.767	1607.002	45	1475.763	1394.902
46	1493.739	57066.551	47	1500.732	9239.986	48	1507.746	1641.049	49	1635.830	55216.924	50	1651.823	3967.873
51	1700.873	2502.849	52	1717.907	41154.163	53	1766.840	601.329	54	1794.882	7257.849	55	1884.971	544.302
56	1897.770	565.198	57	1932.976	15665.156	58	1948.968	2344.735	59	1964.968	456.680	60	2079.079	2622.723
61	2211.181	1551.388	62	2239.215	1123.448	63	2283.240	333.280	64	2506.269	191.921	65	2521.315	644.461
66	3212.620	96.315												

BioTools Version 3.2 Copyright © 1999-2008 Bruker Daltonik GmbH Page -2-

Area 3. Annotated MS spectrum 3.

Spectrum Analysis Report

Sequence Name: L-amino-acid oxidase (Fragments) OS=Macrovipera lebetina PE=1 SV=2 OXLA_MACLB
Parentmass:
Mass Error: MH+ (mono): 1.008 MH+ (avg): 1.008 Threshold (a.i.): 0.000
Tolerance (Da): 0.500 Number of Peaks: 20 Above Threshold: Assigned Peaks:
Not assigned Peaks:

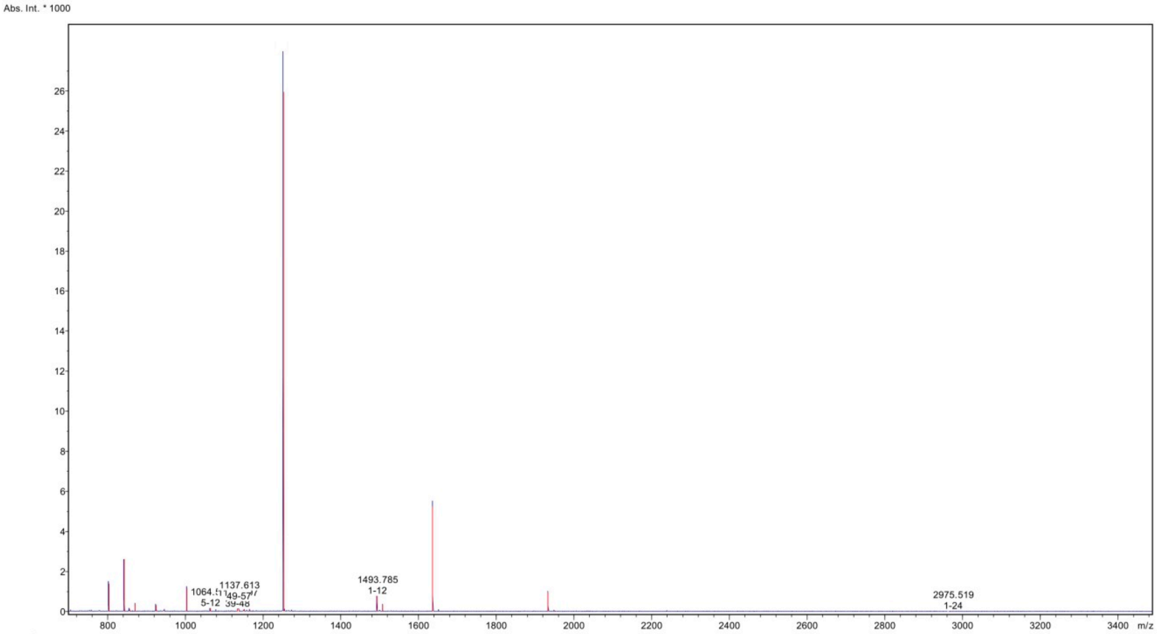

BioTools Version 3.2 Copyright © 1999-2008 Bruker Daltonik GmbH Page -1-

Spectrum Analysis Report

Sequence data:

L-amino-acid oxidase (Fragments) OS=Macrovipera lebetina PE=1 SV=2 OXLA_MACLB

Intensity Coverage: 4.1 % (1659 cnts)
Sequence Coverage MS: 40.2%
Sequence Coverage MS/MS: 0.0%
pI (isoelectric point): 4.4

```
        10         20         30         40         50         60         70         80         90        100        110
ADDKNPLEEC FREDDYEEFL EIAKNGLEGW YANLGPMRYP VKPSEEGKHD DIFAYEKFDE IVGGMDKKFW EDDGIHGGKE TFCYSPMIQK PYQFQHFSEA LTAPVGR
```

Display Parameter:

Sequence Name: L-amino-acid oxidase (Fragments) OS=Macrovipera lebetina PE=1 SV=2 OXLA_MACLB
MH+ (mono): 1.008
MH+ (avg): 1.008 Threshold (a.i.): 0.000 Tolerance (Da): 0.500
Number of Peaks: 20

Peaklist:

Peak	Mass	Intensity	Peak	Mass	Intensity	Peak	Mass	Intensity	Peak	Mass	Intensity	Peak	Mass	Intensity
1	803.479	1388.612	2	842.578	2629.728	3	856.593	140.920	4	870.614	398.020	5	924.593	331.792
6	946.556	64.000	7	1003.629	1171.226	8	1064.576	177.243	9	1078.378	22.000	10	1133.697	143.000
11	1137.613	518.253	12	1151.685	118.000	13	1165.750	86.000	14	1252.718	25956.205	15	1493.785	784.806
16	1507.802	341.611	17	1635.873	5248.347	18	1933.017	965.409	19	1948.993	69.000	20	2975.519	36.000

BioTools Version 3.2 Copyright © 1999-2008 Bruker Daltonik GmbH Page -2-

Area 4. Annotated MS/MS spectrum of ion 2250.354 *m*/*z*.

Spectrum Analysis Report

Sequence Name:		Parentmass:	2250.354	Mass Error:	0.199	MH+ (mono):	2250.155
MH+ (avg):	2251.545	Threshold (a.i.):	0.000	Tolerance (Da):	0.700	Number of Peaks:	109
Above Threshold:	109	Assigned Peaks:	33	Not assigned Peaks:	76		

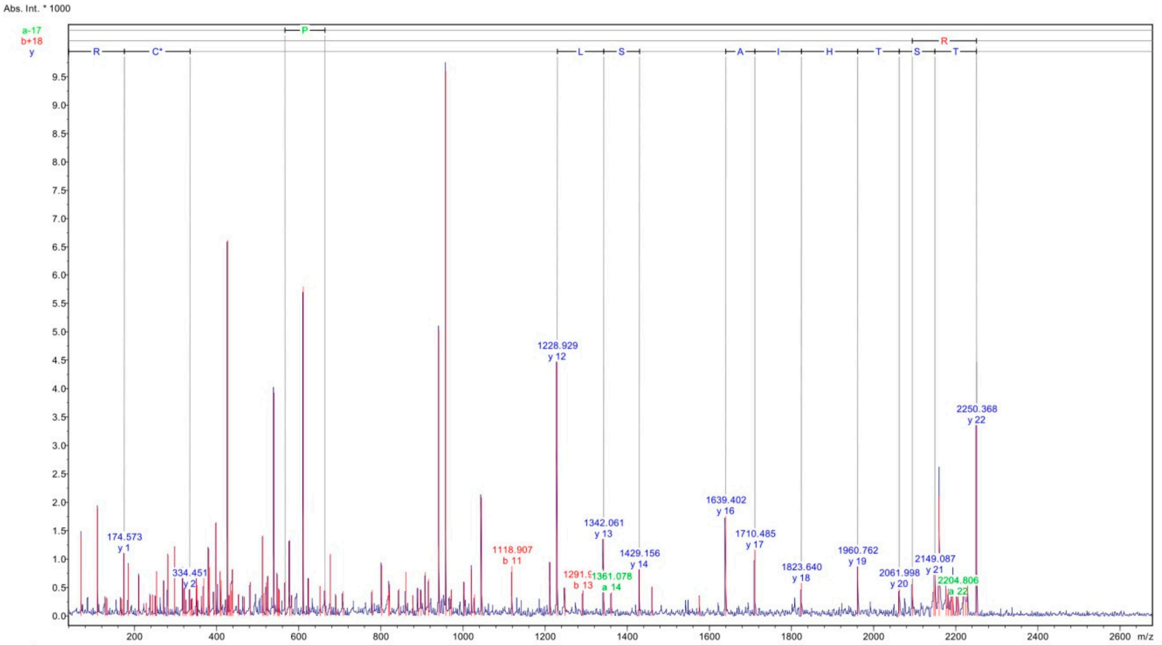

Spectrum Analysis Report

Display Parameter:

Parentmass:	2250.354	Mass Error:	0.199	MH+ (mono):	2250.155	MH+ (avg):	2251.545
Threshold (a.i.):	0.000	Tolerance (Da):	0.700	Number of Peaks:	109	Above Threshold:	109
Assigned Peaks:	33	Not assigned Peaks:	76				

Peaklist:

Peak	Mass	Intensity	Peak	Mass	Intensity	Peak	Mass	Intensity	Peak	Mass	Intensity	Peak	Mass	Intensity
1	69.704	1402.245	2	109.631	1899.934	3	128.623	318.672	4	132.588	298.546	5	166.582	310.720
6	168.570	304.675	7	174.573	1104.464	8	184.552	888.851	9	210.551	714.983	10	228.538	231.931
11	238.497	359.046	12	243.499	367.326	13	250.529	357.402	14	253.493	770.492	15	271.469	616.343
16	281.494	1070.004	17	297.502	1214.483	18	317.452	853.382	19	321.498	496.208	20	325.486	286.637
21	334.451	474.168	22	338.473	283.125	23	340.496	305.625	24	350.487	724.580	25	352.513	541.741
26	362.469	341.713	27	368.486	755.814	28	380.480	1186.026	29	382.476	857.763	30	392.518	427.618
31	398.473	1643.776	32	408.472	792.260	33	410.581	627.543	34	422.484	291.014	35	426.458	6622.632
36	430.504	367.853	37	433.460	560.535	38	435.631	448.353	39	438.469	822.118	40	453.548	386.854
41	467.504	341.025	42	481.546	598.301	43	511.514	1417.175	44	514.999	395.773	45	520.466	592.171
46	524.506	707.346	47	539.502	3926.901	48	547.595	728.977	49	550.518	487.312	50	552.496	457.340
51	566.521	611.199	52	577.487	1334.752	53	582.529	370.241	54	610.534	5796.174	55	623.550	660.622
56	651.535	528.745	57	663.625	455.657	58	676.547	1091.556	59	690.638	359.435	60	707.580	437.191
61	778.621	455.505	62	801.590	903.004	63	818.688	410.378	64	820.695	564.197	65	843.633	477.132
66	860.666	767.027	67	877.687	367.342	68	889.713	493.388	69	897.664	450.252	70	907.726	771.382
71	915.692	655.178	72	940.699	5027.423	73	957.700	9601.370	74	966.892	348.471	75	971.672	469.104
76	1002.803	592.671	77	1020.775	819.767	78	1027.733	378.584	79	1044.746	2082.606	80	1118.907	877.623
81	1211.878	945.787	82	1228.929	4471.034	83	1247.898	502.330	84	1291.986	447.530	85	1342.061	1353.378
86	1361.078	414.893	87	1429.156	819.754	88	1460.105	519.880	89	1575.079	346.618	90	1639.402	1755.679
91	1710.485	1163.936	92	1823.640	576.833	93	1960.762	870.423	94	2061.998	472.485	95	2093.930	546.367
96	2146.371	509.175	97	2149.087	720.897	98	2159.272	2115.605	99	2176.358	472.940	100	2181.962	455.090
101	2187.216	287.295	102	2190.309	559.340	103	2192.759	395.250	104	2202.682	337.659	105	2204.806	339.764
106	2218.044	620.860	107	2223.850	242.034	108	2228.079	541.812	109	2250.368	3356.849			

Calculated Masses:

TSTHIAPLSLPSSPPSVGSVCR 21: Carbamidomethyl (C)

N-Term.	Ion	a	a-17	a-18	b	b-17	b-18	b+18	c	i	x	y	z	C-Term.	Ion
1	T	74.060	57.033	56.049	102.055	85.028	84.044	120.066	119.082	74.060	201.098	**175.119**	158.092	22	R
2	S	161.092	144.066	143.082	189.087	172.060	171.076	207.098	206.114	60.044	361.129	**335.150**	**318.123**	21	C*
3	T	262.140	245.113	**244.129**	290.135	273.108	**272.124**	308.145	307.161	74.060	460.197	434.218	417.191	20	V
4	H	399.199	**382.172**	381.188	427.194	**410.167**	409.183	445.204	444.220	**110.071**	**547.229**	521.250	504.223	19	S
5	I	512.283	495.256	494.272	540.278	523.251	522.267	558.288	557.304	86.096	604.251	578.272	561.245	18	G
6	A	583.320	**566.293**	565.309	611.315	594.288	593.304	629.325	628.341	44.049	703.319	677.340	660.313	17	V
7	P	680.373	**663.346**	662.362	708.368	691.341	**690.357**	726.378	725.394	**70.065**	790.351	764.372	747.345	16	S
8	L	793.457	776.430	775.446	821.452	804.425	803.441	839.462	838.478	86.096	887.404	861.425	844.398	15	P
9	S	880.489	863.462	862.478	908.484	891.457	890.473	926.494	925.510	60.044	984.457	958.477	941.451	14	P
10	L	993.573	976.546	975.562	1021.568	1004.541	1003.557	1039.578	1038.594	86.096	1071.489	1045.510	1028.483	13	S
11	P	1090.626	1073.599	1072.615	**1118.620**	1101.594	1100.610	1136.631	1135.647	**70.065**	1158.521	1132.542	1115.515	12	S
12	S	1177.658	1160.631	1159.647	1205.652	1188.626	1187.642	1223.663	1222.679	60.044	1255.574	**1229.594**	**1212.568**	11	P
13	S	1264.690	**1247.663**	1246.679	**1292.684**	1275.658	1274.674	1310.695	1309.711	60.044	1368.658	**1342.678**	1325.652	10	L
14	P	**1361.742**	1344.716	1343.732	1389.737	1372.711	1371.727	1407.748	1406.764	**70.065**	1455.690	**1429.710**	1412.684	9	S
15	P	1458.795	1441.769	1440.785	1486.790	1469.763	1468.779	1504.801	1503.817	**70.065**	1568.774	1542.794	1525.768	8	L
16	S	1545.827	1528.801	1527.817	1573.822	1556.795	1555.811	1591.833	1590.849	60.044	1665.826	**1639.847**	1622.821	7	P
17	V	1644.896	1627.869	1626.885	1672.890	1655.864	1654.880	1690.901	1689.917	72.081	1736.864	**1710.884**	1693.858	6	A
18	G	1701.917	1684.890	1683.906	1729.912	1712.885	1711.901	1747.922	1746.938	30.034	1849.948	**1823.968**	1806.942	5	I
19	S	1788.949	1771.922	1770.938	1816.944	1799.917	1798.933	1834.955	1833.971	60.044	1987.007	**1961.027**	1944.001	4	H
20	V	1888.017	1870.991	1870.007	1916.012	1898.986	1898.002	1934.023	1933.039	72.081	2088.054	**2062.075**	2045.048	3	T
21	C*	2048.048	2031.022	2030.038	2076.043	2059.016	2058.032	**2094.054**	2093.070	**133.043**	2175.086	**2149.107**	2132.080	2	S
22	R	**2204.149**	**2187.123**	2186.139	2232.144	2215.118	2214.134	**2250.155**	2249.171	**129.113**	2276.134	**2250.155**	2233.128	1	T

Area 4. Annotated MS/MS spectrum of ion 1174.611 *m*/*z*.

Spectrum Analysis Report

Sequence Name:		Parentmass:	1174.611	Mass Error:	0.007	MH+ (mono):	1174.604
MH+ (avg):	1175.384	Threshold (a.i.):	0.000	Tolerance (Da):	0.700	Number of Peaks:	39
Above Threshold:	39	Assigned Peaks:	19	Not assigned Peaks:	20		

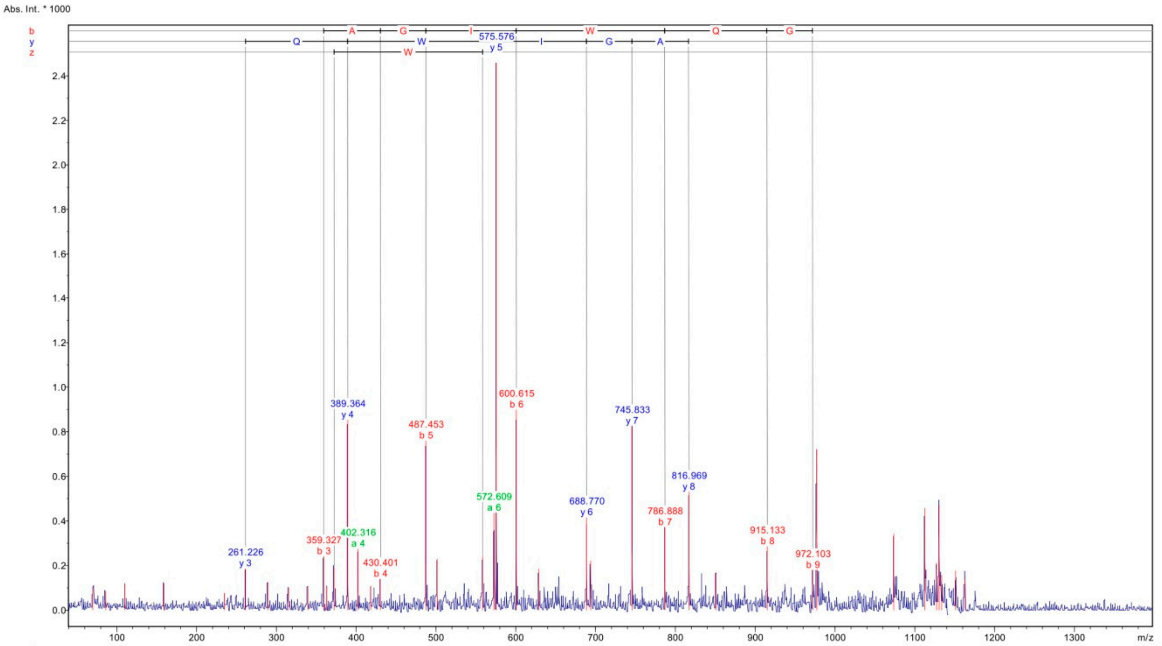

 Copyright © 1999-2008 Bruker Daltonik GmbH

Spectrum Analysis Report

Display Parameter:

Parentmass:	1174.611	Mass Error:	0.007	MH+ (mono):	1174.604	MH+ (avg):	1175.384
Threshold (a.i.):	0.000	Tolerance (Da):	0.700	Number of Peaks:	39	Above Threshold:	39
Assigned Peaks:	19	Not assigned Peaks:	20				

Peaklist:

Peak	Mass	Intensity	Peak	Mass	Intensity	Peak	Mass	Intensity	Peak	Mass	Intensity	Peak	Mass	Intensity
1	70.050	101.997	2	86.072	85.428	3	110.052	115.615	4	159.122	120.026	5	235.150	77.562
6	261.226	187.337	7	289.217	127.517	8	315.284	103.203	9	339.324	109.214	10	359.327	241.524
11	363.316	106.219	12	372.354	192.287	13	389.364	853.476	14	402.316	275.784	15	418.353	109.686
16	430.401	140.629	17	487.453	760.209	18	501.477	229.955	19	558.522	241.919	20	572.609	436.554
21	575.576	2504.424	22	600.615	899.637	23	628.666	185.665	24	688.770	416.279	25	693.785	222.439
26	745.833	825.995	27	786.888	372.179	28	816.969	530.552	29	851.051	166.463	30	915.133	286.600
31	972.103	180.034	32	977.216	722.641	33	1073.466	342.881	34	1112.526	457.598	35	1127.186	213.070
36	1130.557	470.899	37	1133.770	158.144	38	1151.241	177.595	39	1162.446	142.045			

Calculated Masses:
VVCAGIWQGGK 3: Carbamidomethyl (C)

N-Term.	Ion	a	a-17	a-18	b	b-17	b-18	b+18	c	i	x	y	z	C-Term.	Ion
1	V	72.081	55.054	54.070	100.076	83.049	82.065	118.086	117.102	72.081	173.092	147.113	130.086	11	K
2	V	171.149	154.123	153.139	199.144	182.118	181.134	217.155	216.171	72.081	230.114	204.134	187.108	10	G
3	C*	331.180	314.153	313.169	**359.175**	342.148	341.164	377.185	376.201	133.043	287.135	**261.156**	244.129	9	G
4	A	**402.217**	385.190	384.206	**430.212**	413.185	412.201	448.222	447.238	44.049	415.194	**389.214**	**372.188**	8	Q
5	G	459.238	442.212	441.228	**487.233**	470.207	469.223	505.244	504.260	30.034	**601.273**	**575.294**	**558.267**	7	W
6	I	**572.322**	555.296	554.312	**600.317**	583.291	582.307	618.328	617.344	**86.096**	714.357	**688.378**	671.351	6	I
7	W	758.402	741.375	740.391	**786.397**	769.370	768.386	804.407	803.423	**159.092**	771.378	**745.399**	728.373	5	G
8	Q	886.460	869.434	868.450	**914.455**	897.429	896.445	932.466	931.482	101.071	842.416	**816.436**	799.410	4	A
9	G	943.482	926.455	925.471	**971.477**	954.450	953.466	989.487	988.503	30.034	1002.446	976.467	959.440	3	C*
10	G	1000.503	983.477	982.493	1028.498	1011.472	1010.488	1046.509	1045.525	30.034	1101.515	1075.535	1058.509	2	V
11	K	1128.598	1111.572	1110.588	1156.593	1139.567	1138.583	1174.604	1173.620	101.107	1200.583	1174.604	1157.577	1	V

 Copyright © 1999-2008 Bruker Daltonik GmbH

Area 4. Annotated MS/MS spectrum of ion 1235.644 *m*/*z*.

Spectrum Analysis Report

Sequence Name:		Parentmass:	1235.644	Mass Error:	0.023	MH+ (mono):	1235.621
MH+ (avg):	1236.424	Threshold (a.i.):	0.000	Tolerance (Da):	0.700	Number of Peaks:	28
Above Threshold:	28	Assigned Peaks:	19	Not assigned Peaks:	9		

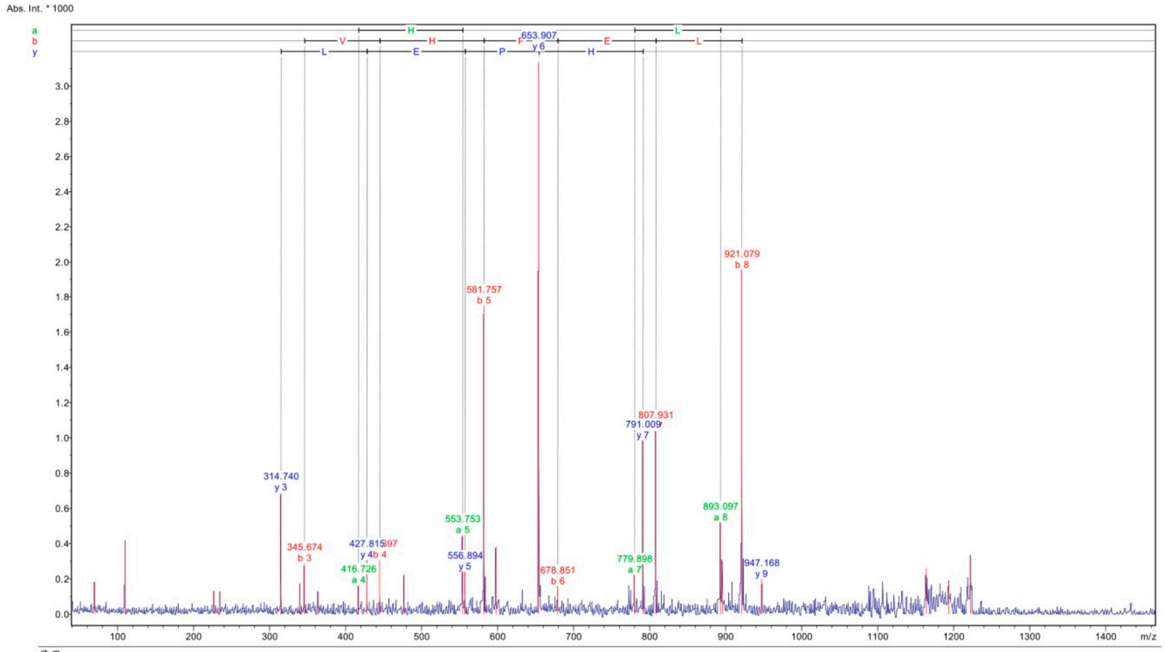

Spectrum Analysis Report

Display Parameter:

Parentmass:	1235.644	Mass Error:	0.023	MH+ (mono):	1235.621	MH+ (avg):	1236.424
Threshold (a.i.):	0.000	Tolerance (Da):	0.700	Number of Peaks:	28	Above Threshold:	28
Assigned Peaks:	19	Not assigned Peaks:	9				

Peaklist:

Peak	Mass	Intensity	Peak	Mass	Intensity	Peak	Mass	Intensity	Peak	Mass	Intensity	Peak	Mass	Intensity
1	69.801	181.133	2	109.748	418.087	3	226.681	132.129	4	234.701	128.984	5	314.740	690.275
6	339.725	177.557	7	345.674	288.488	8	363.688	129.053	9	416.726	164.709	10	427.815	308.084
11	444.697	307.910	12	476.743	225.134	13	553.753	448.557	14	556.894	241.612	15	581.757	1749.883
16	597.762	385.589	17	653.907	3196.613	18	678.851	157.215	19	779.898	223.123	20	791.009	986.103
21	807.931	1038.627	22	893.097	519.896	23	895.815	301.669	24	921.079	1955.584	25	947.168	199.627
26	1163.943	259.224	27	1193.662	184.929	28	1222.296	321.576						

Calculated Masses:
CQGVHPELPAK 1: Carbamidomethyl (C)

N-Term.	Ion	a	a-17	a-18	b	b-17	b-18	b+18	c	i	x	y	z	C-Term.	Ion
1	C*	133.043	116.016	115.032	161.038	144.011	143.027	179.048	178.064	133.043	173.092	147.113	130.086	11	K
2	Q	261.102	244.075	243.091	289.097	272.070	271.086	307.107	306.123	101.071	244.129	218.150	201.123	10	A
3	G	318.123	301.097	300.112	**346.118**	329.091	328.107	**364.129**	**363.145**	30.034	341.182	**315.203**	298.176	9	P
4	V	**417.191**	400.165	399.181	**445.186**	**428.160**	**427.176**	463.197	462.213	72.081	454.266	**428.287**	411.260	8	L
5	H	**554.250**	537.224	536.240	**582.245**	565.219	564.235	600.256	599.272	**110.071**	583.309	**557.329**	540.303	7	E
6	P	651.303	634.277	633.293	**679.298**	662.272	661.287	697.309	696.325	**70.065**	680.361	**654.382**	637.356	6	P
7	E	**780.346**	763.319	762.335	**808.341**	**791.314**	**790.330**	826.351	825.367	102.055	817.420	**791.441**	774.414	5	H
8	L	**893.430**	876.403	875.419	**921.425**	904.398	903.414	939.435	938.451	86.096	916.489	890.509	873.483	4	V
9	P	990.483	973.456	972.472	1018.477	1001.451	1000.467	1036.488	1035.504	**70.065**	973.510	**947.531**	930.504	3	G
10	A	1061.520	1044.493	1043.509	1089.515	1072.488	1071.504	1107.525	1106.541	44.049	1101.569	1075.589	1058.563	2	Q
11	K	1189.615	1172.588	1171.604	1217.610	1200.583	1199.599	1235.620	1234.636	101.107	1261.599	1235.620	1218.594	1	C*

Area 4. Annotated MS spectrum 1.

Spectrum Analysis Report

Sequence Name: Snake venom serine protease nikobin OS=Vipera nikolskii GN=sp-VN PE=2 SV=1 VSP_VIPNI
Parentmass:
Mass Error:
MH+ (mono): 1.008
MH+ (avg): 1.008
Threshold (a.i.): 0.000
Tolerance (Da): 0.500
Number of Peaks: 69
Above Threshold:
Assigned Peaks:
Not assigned Peaks:

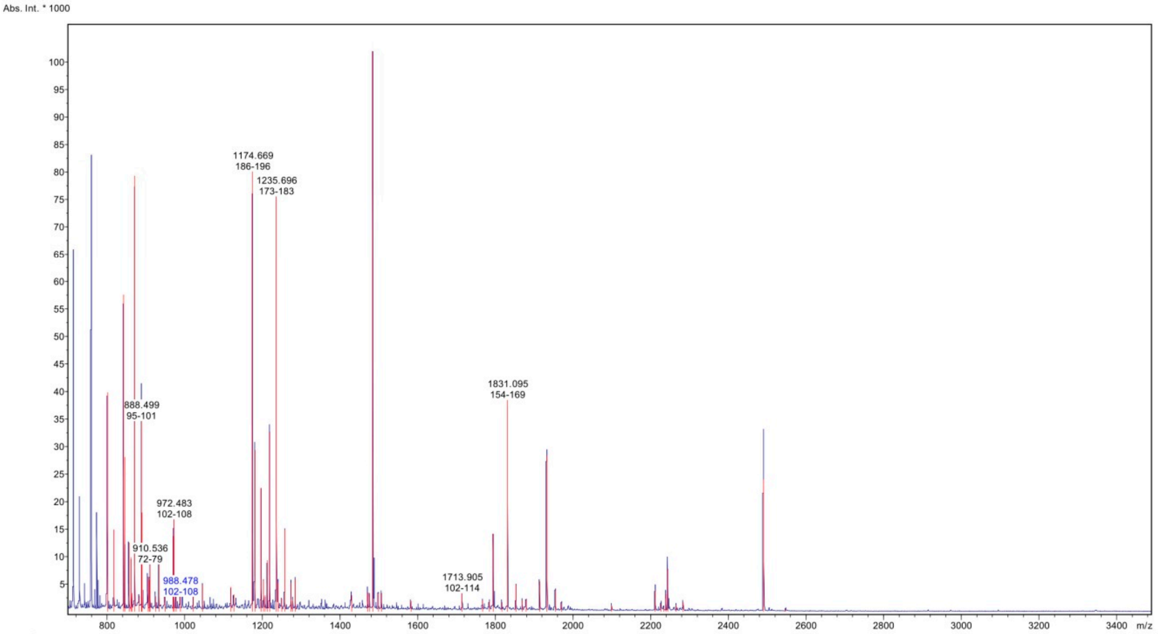

Spectrum Analysis Report

Sequence data:

Snake venom serine protease nikobin OS=Vipera nikolskii GN=sp-VN PE=2 SV=1 VSP_VIPNI

Intensity Coverage: 26.3 % (254171 cnts)
Sequence Coverage MS: 25.7%
Sequence Coverage MS/MS: 0.0%
pI (isoelectric point): 9.3

10	20	30	40	50	60	70	80	90	100	110	120	130	140
MVLIRVLANL	LLLQLSYAQK	SSELVIGGDE	CNINEHPFLA	FVTSDRRRCA	GTLINQEWVL	TAAHCNGKYM	KIELGVHDKM	VRNEDKQTRV	PKQKFFCLSS	KEYTMWDKDI	MLIRLNTPVN	NSTHIAPVSL	ASRPPVVGSV
**150**	**160**	**170**	**180**	**190**	**200**	**210**	**220**	**230**	**240**	**250**	**260**		
CRIMGWGTIS	SPKVILPDVP	HCANIEIIKY	SKCQGVHPEL	PAKGRVVCAG	IWQGGKDSCH	GDSGAPLICN	GQLQGLLSWG	GDPCAQPLQP	GLYTDIFDYS	DWIQSIIAGN	TTATCPP		

Display Parameter:

Sequence Name: Snake venom serine protease nikobin OS=Vipera nikolskii GN=sp-VN PE=2 SV=1 VSP_VIPNI
MH+ (mono): 1.008
MH+ (avg): 1.008
Threshold (a.i.): 0.000
Tolerance (Da): 0.500
Number of Peaks: 69

Peaklist:

Peak	Mass	Intensity	Peak	Mass	Intensity	Peak	Mass	Intensity	Peak	Mass	Intensity	Peak	Mass	Intensity
1	801.550	39834.187	2	817.523	14963.576	3	842.571	57572.624	4	845.580	28093.509	5	856.586	12519.786
6	861.552	9883.100	7	864.551	3400.430	8	870.606	79303.799	9	882.622	2894.025	10	888.499	34598.894
11	889.610	18013.919	12	905.582	6376.282	13	908.549	5781.317	14	910.536	8590.279	15	924.494	2756.636
16	933.631	10000.323	17	949.597	4296.209	18	970.581	13745.887	19	972.483	16773.744	20	977.664	5574.950
21	988.478	2695.049	22	1021.692	3268.343	23	1045.632	4927.422	24	1118.654	4515.914	25	1126.631	3151.449
26	1174.669	80038.090	27	1180.673	29332.917	28	1196.669	22467.562	29	1202.668	5714.930	30	1206.666	2979.560
31	1212.652	9352.284	32	1218.664	32682.370	33	1235.696	75510.299	34	1240.662	5325.754	35	1249.701	2419.854
36	1257.689	14875.203	37	1273.667	5295.622	38	1277.763	2170.663	39	1284.762	5925.101	40	1429.797	2991.299
41	1471.193	3474.314	42	1475.833	3285.354	43	1484.886	105996.575	44	1498.888	3315.789	45	1506.865	3420.735
46	1582.770	1942.570	47	1713.905	3342.931	48	1766.867	2051.617	49	1783.928	1813.310	50	1794.892	14152.249
51	1816.893	1740.194	52	1831.095	38458.801	53	1853.098	4845.198	54	1869.075	2110.683	55	1879.143	2209.982
56	1913.954	5512.612	57	1931.975	28365.400	58	1953.972	3843.281	59	1969.953	1606.469	60	2098.992	1148.578
61	2211.175	3882.271	62	2226.133	1423.716	63	2233.195	762.463	64	2239.209	3152.196	65	2244.136	7794.219
66	2265.151	1225.987	67	2283.226	1765.606	68	2490.260	23985.453	69	2547.272	463.445			

Area 4. Annotated MS spectrum 2.

Spectrum Analysis Report

Sequence Name: Snake venom serine protease nikobin OS=Vipera nikolskii GN=sp-VN PE=2 SV=1 VSP_VIPNI
Parentmass:
Mass Error: MH+ (mono): 1.008 MH+ (avg): 1.008 Threshold (a.i.): 0.000
Tolerance (Da): 0.500 Number of Peaks: 76 Above Threshold: Assigned Peaks:
Not assigned Peaks:

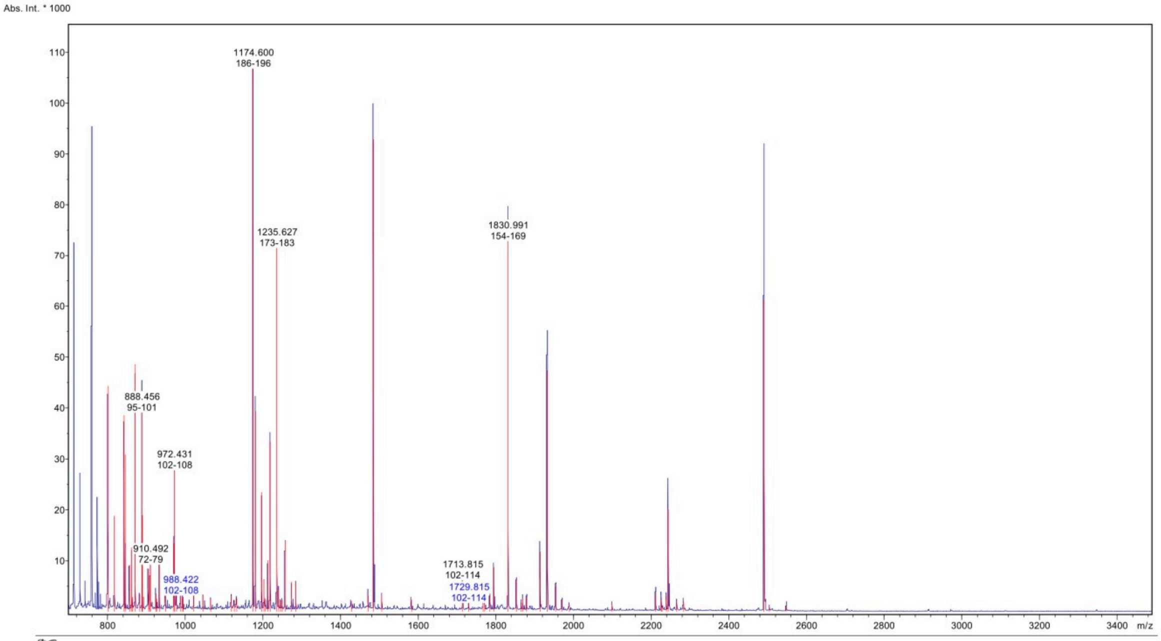

Copyright © 1999-2008 Bruker Daltonik GmbH

Spectrum Analysis Report

Sequence data:

Snake venom serine protease nikobin OS=Vipera nikolskii GN=sp-VN PE=2 SV=1 VSP_VIPNI

Intensity Coverage: 29.3 % (321607 cnts)
Sequence Coverage MS: 25.7%
Sequence Coverage MS/MS: 0.0%
pI (isoelectric point): 9.3

10 20 30 40 50 60 70 80 90 100 110 120 130 140
MVLIRVLANL LLLQLSYAQK SSELVIGGDE CNINEHPFLA FVTSDRRRCA GTLINQEWVL TAAHCNGKYM KIELGVHDKM VRNEDKQTRV PKQKFFCLSS KEYTMWDKDI MLIRLNTPVN NSTHIAPVSL ASRPPVVGSV

150 160 170 180 190 200 210 220 230 240 250 260
CRIMGWGTIS SPKVILPDVP HCANIEIIKY SKCQGVHPEL PAKGRVVCAG IWQGGKDSCH GDSGAPLICN GQLQGLLSWG GDPCAQPLQP GLYTDIFDYS DWIQSIIAGN TTATCPP

Display Parameter:

Sequence Name: Snake venom serine protease nikobin OS=Vipera nikolskii GN=sp-VN PE=2 SV=1 VSP_VIPNI
MH+ (mono): 1.008
MH+ (avg): 1.008 Threshold (a.i.): 0.000 Tolerance (Da): 0.500
Number of Peaks: 76

Peaklist:

Peak	Mass	Intensity	Peak	Mass	Intensity	Peak	Mass	Intensity	Peak	Mass	Intensity	Peak	Mass	Intensity
1	801.515	44316.243	2	817.488	18882.076	3	842.530	38547.398	4	845.542	30888.027	5	856.543	9184.432
6	861.514	12622.246	7	864.518	2867.780	8	870.565	48609.999	9	882.578	3402.528	10	888.456	39066.048
11	889.570	18924.302	12	905.536	8419.885	13	908.504	7142.005	14	910.492	9224.758	15	924.440	3565.763
16	926.416	2537.890	17	933.587	11548.972	18	949.563	5219.277	19	970.530	13400.134	20	972.431	27765.765
21	977.619	6963.394	22	988.422	3131.025	23	993.567	2604.110	24	994.407	2935.533	25	1021.643	3887.691
26	1045.578	3184.254	27	1065.656	2519.104	28	1118.592	3101.777	29	1126.577	2284.741	30	1132.615	2573.014
31	1174.600	106752.718	32	1180.602	39345.824	33	1196.606	23469.442	34	1202.604	6225.679	35	1206.602	2612.644
36	1212.591	10105.994	37	1218.593	33313.036	38	1235.627	71475.766	39	1240.596	4267.915	40	1245.575	2229.363
41	1249.625	2347.495	42	1257.633	13795.346	43	1273.609	5928.280	44	1284.689	5738.854	45	1427.766	2093.672
46	1471.106	3112.069	47	1484.800	92853.593	48	1505.775	2991.055	49	1582.683	2378.287	50	1713.815	6134.549
51	1729.815	1701.926	52	1766.783	1599.095	53	1770.834	2895.387	54	1783.840	3097.811	55	1794.785	8731.265
56	1830.991	72802.734	57	1853.002	6422.255	58	1865.033	1823.148	59	1868.969	2863.325	60	1879.036	2790.443
61	1913.863	11742.464	62	1931.869	47397.329	63	1953.870	4963.527	64	1969.848	2331.196	65	1988.909	1296.369
66	2098.887	1401.806	67	2211.065	3549.537	68	2226.009	2334.889	69	2227.982	1187.188	70	2239.093	3068.230
71	2244.022	20005.504	72	2265.032	1930.839	73	2283.107	1912.625	74	2490.140	61302.645	75	2504.145	852.873
76	2547.156	1246.504												

Copyright © 1999-2008 Bruker Daltonik GmbH

Area 4. Annotated MS/MS spectrum of ion 972.471 *m*/*z*.

Spectrum Analysis Report

Sequence Name:		Parentmass:	972.471	Mass Error:	0.058	MH+ (mono):	972.414
MH+ (avg):	973.083	Threshold (a.i.):	0.000	Tolerance (Da):	0.700	Number of Peaks:	50
Above Threshold:	50	Assigned Peaks:	19	Not assigned Peaks:	31		

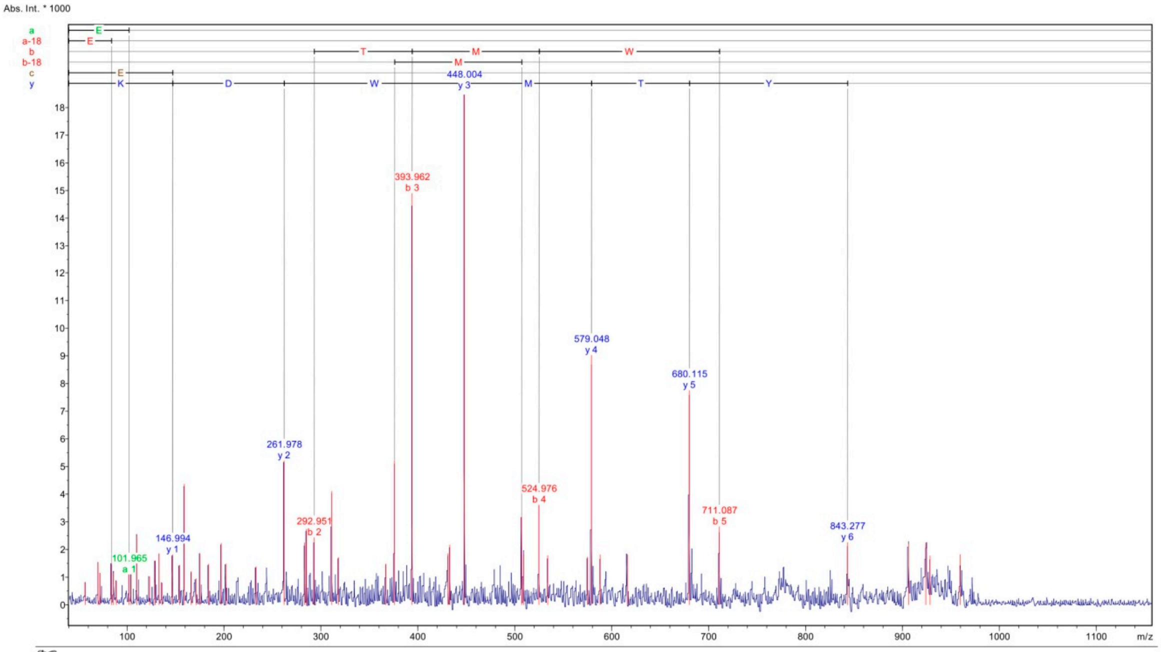

BioTools Version 3.2 Copyright © 1999-2008 Bruker Daltonik GmbH Page -1-

Spectrum Analysis Report

Display Parameter:

Parentmass:	972.471	Mass Error:	0.058	MH+ (mono):	972.414	MH+ (avg):	973.083
Threshold (a.i.):	0.000	Tolerance (Da):	0.700	Number of Peaks:	50	Above Threshold:	50
Assigned Peaks:	19	Not assigned Peaks:	31				

Peaklist:

Peak	Mass	Intensity	Peak	Mass	Intensity	Peak	Mass	Intensity	Peak	Mass	Intensity	Peak	Mass	Intensity
1	56.973	829.496	2	69.981	1503.596	3	72.001	1155.524	4	83.980	1474.844	5	85.981	1169.402
6	88.982	858.964	7	101.965	1101.256	8	103.970	1549.259	9	109.963	2518.579	10	122.984	1031.361
11	128.998	1603.410	12	132.964	1862.959	13	135.971	802.770	14	146.994	1819.768	15	153.972	1441.114
16	158.975	4372.663	17	165.994	1217.130	18	170.963	961.834	19	174.975	1856.325	20	183.976	1486.099
21	196.994	2240.549	22	201.969	1494.273	23	232.958	1395.919	24	261.978	5217.631	25	283.082	2256.590
26	285.023	2745.510	27	292.951	2443.301	28	311.134	4119.536	29	317.993	1721.690	30	367.039	1492.483
31	375.960	5222.764	32	393.962	14905.571	33	431.000	1866.568	34	432.954	2174.708	35	448.004	18619.124
36	506.989	3194.571	37	508.993	1839.846	38	524.976	3621.844	39	533.975	1799.542	40	575.058	1741.496
41	579.048	9031.054	42	588.027	1820.581	43	616.031	1820.650	44	680.115	7760.318	45	711.087	2834.050
46	843.277	2264.893	47	906.090	2202.201	48	923.995	2249.990	49	928.423	1777.919	50	959.645	1828.821

Calculated Masses:
EYTMWDK

N-Term.	Ion	a	a-17	a-18	b	b-17	b-18	b+18	c	i	x	y	z	C-Term.	Ion
1	E	**102.055**	85.028	**84.044**	130.050	113.023	112.039	148.060	**147.076**	**102.055**	173.092	**147.113**	130.086	7	K
2	Y	265.118	248.092	247.108	**293.113**	276.087	275.103	**311.124**	310.140	**136.076**	288.119	**262.140**	245.113	6	D
3	T	366.166	349.139	348.155	**394.161**	377.134	**376.150**	412.171	411.187	74.060	474.198	**448.219**	**431.193**	5	W
4	M	497.206	480.180	479.196	**525.201**	508.175	**507.191**	543.212	542.228	**104.053**	605.239	**579.260**	562.233	4	M
5	W	683.286	666.259	665.275	**711.281**	694.254	693.270	729.291	728.307	**159.092**	706.286	**680.307**	663.281	3	T
6	D	798.313	781.286	780.302	826.308	809.281	808.297	844.318	**843.334**	88.039	869.350	**843.371**	826.344	2	Y
7	K	926.408	909.381	908.397	954.403	937.376	936.392	972.413	971.429	101.107	998.392	972.413	955.387	1	E

BioTools Version 3.2 Copyright © 1999-2008 Bruker Daltonik GmbH Page -2-

Area 4. Annotated MS/MS spectrum of ion 1831.064 *m*/*z*.

Spectrum Analysis Report

Sequence Name:		Parentmass:	1831.064	Mass Error:	0.048	MH+ (mono):	1831.015
MH+ (avg):	1832.197	Threshold (a.i.):	0.000	Tolerance (Da):	0.700	Number of Peaks:	88
Above Threshold:	88	Assigned Peaks:	42	Not assigned Peaks:	46		

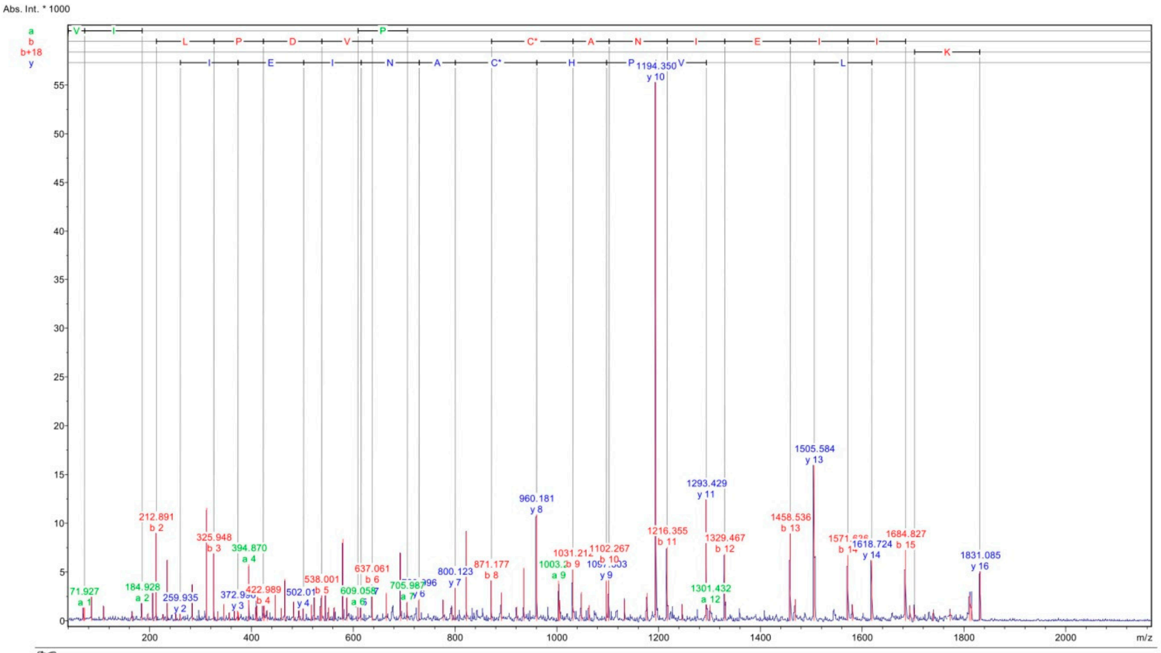

BioTools Version 3.2 Copyright © 1999-2008 Bruker Daltonik GmbH Page -1-

Spectrum Analysis Report

Display Parameter:

Parentmass:	1831.064	Mass Error:	0.048	MH+ (mono):	1831.015	MH+ (avg):	1832.197
Threshold (a.i.):	0.000	Tolerance (Da):	0.700	Number of Peaks:	88	Above Threshold:	88
Assigned Peaks:	42	Not assigned Peaks:	46				

Peaklist:

Peak	Mass	Intensity	Peak	Mass	Intensity	Peak	Mass	Intensity	Peak	Mass	Intensity	Peak	Mass	Intensity
1	69.907	1966.970	2	71.927	1351.990	3	85.934	3142.713	4	109.870	1481.515	5	165.834	960.110
6	184.928	1810.875	7	196.895	806.859	8	206.876	2861.971	9	212.891	8999.850	10	226.920	684.187
11	234.872	6288.193	12	251.891	1149.003	13	259.935	714.319	14	283.905	3763.893	15	311.888	11589.454
16	325.948	6902.517	17	333.911	912.807	18	345.849	1737.566	19	356.928	875.041	20	366.879	1987.455
21	372.996	1014.525	22	374.914	887.414	23	394.870	5798.055	24	408.927	1441.182	25	410.927	2142.645
26	422.989	1542.229	27	424.952	2625.377	28	430.896	1107.935	29	446.967	3578.238	30	458.881	1312.167
31	465.890	4306.174	32	482.906	1999.816	33	493.934	1093.310	34	502.019	1279.171	35	517.981	1675.845
36	523.997	3058.916	37	535.922	1475.297	38	538.001	2600.792	39	545.950	3315.117	40	551.930	1409.607
41	562.927	1410.641	42	579.907	8401.381	43	587.942	2445.924	44	609.058	1418.051	45	615.067	1363.506
46	637.061	3697.697	47	664.999	2906.010	48	692.992	6947.665	49	705.987	1966.974	50	729.096	2237.539
51	777.044	2149.433	52	794.060	1416.023	53	800.123	3384.932	54	822.032	9137.087	55	871.177	4135.491
56	891.061	2927.755	57	921.092	1470.341	58	935.112	5451.750	59	960.181	10875.962	60	1003.246	4126.474
61	1006.231	2143.709	62	1031.212	5278.714	63	1048.211	2974.510	64	1063.335	1603.712	65	1097.303	4151.842
66	1102.267	5750.599	67	1133.268	2166.408	68	1177.307	2863.056	69	1194.350	55309.982	70	1216.355	7562.069
71	1246.337	1641.877	72	1293.429	12425.309	73	1301.432	1656.863	74	1329.467	6849.995	75	1458.536	8963.918
76	1468.673	2235.222	77	1505.584	15980.754	78	1571.636	6768.149	79	1581.118	1655.940	80	1618.724	6205.549
81	1684.827	7240.957	82	1693.389	1482.703	83	1702.832	1381.715	84	1740.200	961.052	85	1772.879	956.534
86	1811.148	2635.460	87	1814.570	1710.355	88	1831.085	5062.948						

Calculated Masses:
VILPDVPHCANIEIIK 9: Carbamidomethyl (C)

N-Term.	Ion	a	a-17	a-18	b	b-17	b-18	b+18	c	i	x	y	z	C-Term.	Ion
1	V	**72.081**	55.054	54.070	100.076	83.049	82.065	118.086	117.102	**72.081**	173.092	147.113	130.086	16	K
2	I	**185.165**	168.138	167.154	**213.160**	196.133	195.149	231.170	230.186	**86.096**	286.176	**260.197**	243.170	15	I
3	L	298.249	281.222	280.238	**326.244**	309.217	308.233	344.254	343.270	**86.096**	399.260	**373.281**	**356.254**	14	I
4	P	**395.302**	378.275	377.291	**423.297**	406.270	405.286	441.307	440.323	**70.065**	528.303	**502.324**	485.297	13	E
5	D	510.329	**493.302**	492.318	**538.324**	521.297	520.313	556.334	555.350	88.039	641.387	**615.408**	598.381	12	I
6	V	**609.397**	592.370	591.386	**637.392**	620.365	619.381	655.403	654.418	**72.081**	755.430	**729.451**	712.424	11	N
7	P	**706.450**	689.423	688.439	734.445	717.418	716.434	752.455	751.471	**70.065**	826.467	**800.488**	783.461	10	A
8	H	843.509	826.482	825.498	**871.504**	854.477	853.493	889.514	888.530	**110.071**	986.498	**960.518**	943.492	9	C*
9	C*	**1003.539**	986.513	985.529	**1031.534**	1014.508	1013.524	1049.545	**1048.561**	133.043	1123.556	**1097.577**	1080.551	8	H
10	A	1074.576	1057.550	1056.566	**1102.571**	1085.545	1084.561	1120.582	1119.598	44.049	1220.609	**1194.630**	**1177.603**	7	P
11	N	1188.619	1171.593	1170.609	**1216.614**	1199.588	1198.604	1234.625	1233.641	87.055	1319.678	**1293.698**	1276.672	6	V
12	I	**1301.703**	1284.677	1283.693	**1329.698**	1312.672	1311.688	1347.709	1346.725	**86.096**	1434.705	1408.725	1391.699	5	D
13	E	1430.746	1413.719	1412.735	**1458.741**	1441.714	1440.730	1476.752	1475.768	102.055	1531.757	**1505.778**	1488.752	4	P
14	I	1543.830	1526.804	1525.820	**1571.825**	1554.798	1553.814	1589.836	1588.852	**86.096**	1644.841	**1618.862**	1601.836	3	L
15	I	1656.914	1639.888	1638.904	**1684.909**	1667.883	1666.899	**1702.920**	1701.936	**86.096**	1757.925	1731.946	1714.920	2	I
16	K	1785.009	1767.983	1766.999	1813.004	1795.978	1794.993	**1831.015**	1830.031	101.107	1856.994	**1831.015**	**1813.988**	1	V

BioTools Version 3.2 Copyright © 1999-2008 Bruker Daltonik GmbH Page -2-

Area 5. Annotated MS/MS spectrum of ion 1512.781 *m*/*z*.

Spectrum Analysis Report

Sequence Name:		Parentmass:	1512.781	Mass Error:	0.094	MH+ (mono):	1512.687
MH+ (avg):	1513.616	Threshold (a.i.):	0.000	Tolerance (Da):	0.700	Number of Peaks:	30
Above Threshold:	30	Assigned Peaks:	19	Not assigned Peaks:	11		

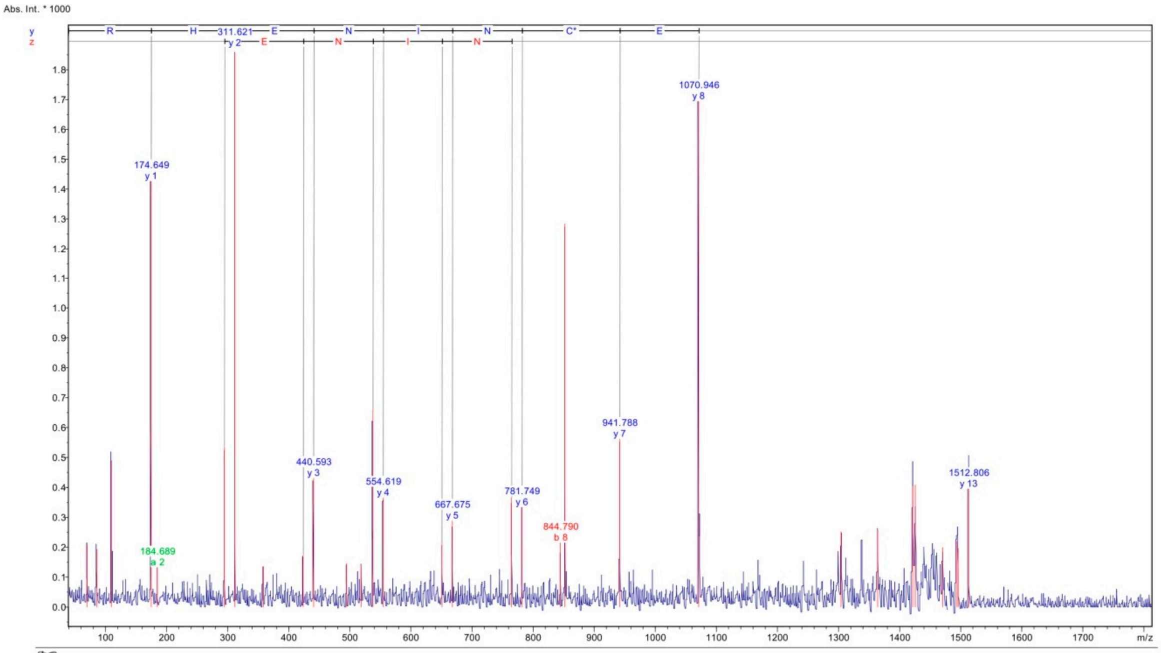

BioTools Version 3.2 Copyright © 1999-2008 Bruker Daltonik GmbH Page -1-

Spectrum Analysis Report

Display Parameter:

Parentmass:	1512.781	Mass Error:	0.094	MH+ (mono):	1512.687	MH+ (avg):	1513.616
Threshold (a.i.):	0.000	Tolerance (Da):	0.700	Number of Peaks:	30	Above Threshold:	30
Assigned Peaks:	19	Not assigned Peaks:	11				

Peaklist:

Peak	Mass	Intensity	Peak	Mass	Intensity	Peak	Mass	Intensity	Peak	Mass	Intensity	Peak	Mass	Intensity
1	69.682	206.384	2	85.747	194.033	3	109.699	490.776	4	174.649	1426.767	5	184.689	132.349
6	294.610	535.696	7	311.621	1872.651	8	358.525	136.449	9	423.598	172.522	10	440.593	431.561
11	494.607	146.687	12	518.534	140.528	13	537.592	658.944	14	554.619	365.902	15	650.650	214.409
16	667.675	288.688	17	764.743	366.633	18	781.749	333.828	19	844.790	215.131	20	851.799	1284.091
21	941.788	562.711	22	1070.946	1694.309	23	1303.875	252.813	24	1364.220	258.583	25	1421.415	404.718
26	1425.493	408.885	27	1470.553	200.507	28	1493.692	221.755	29	1495.691	196.822	30	1512.806	395.537

Calculated Masses:
VIGGDECNINEHR 7: Carbamidomethyl (C)

N-Term.	Ion	a	a-17	a-18	b	b-17	b-18	b+18	c	i	x	y	z	C-Term.	Ion
1	V	72.081	55.054	54.070	100.076	83.049	82.065	118.086	117.102	72.081	201.098	**175.119**	158.092	13	R
2	I	**185.165**	168.138	167.154	213.160	196.133	195.149	231.170	230.186	**86.096**	338.157	**312.178**	**295.151**	12	H
3	G	242.186	225.160	224.176	270.181	253.155	252.171	288.192	287.208	30.034	467.200	**441.220**	**424.194**	11	E
4	G	299.208	282.181	281.197	327.203	310.176	309.192	345.213	344.229	30.034	581.243	**555.263**	**538.237**	10	N
5	D	414.235	397.208	396.224	442.230	425.203	**424.219**	460.240	459.256	88.039	694.327	**668.347**	**651.321**	9	I
6	E	543.277	526.251	525.267	571.272	**554.246**	553.262	589.283	588.299	102.055	808.370	**782.390**	**765.364**	8	N
7	C*	703.308	686.281	685.297	731.303	714.276	713.292	749.313	748.329	133.043	968.400	**942.421**	925.394	7	C*
8	N	817.351	800.324	799.340	**845.346**	828.319	827.335	863.356	862.372	87.055	1097.443	**1071.464**	1054.437	6	E
9	I	930.435	913.408	912.424	958.430	**941.403**	940.419	976.440	975.456	**86.096**	1212.470	1186.491	1169.464	5	D
10	N	1044.478	1027.451	1026.467	1072.473	1055.446	1054.462	1090.483	1089.499	87.055	1269.491	1243.512	1226.485	4	G
11	E	1173.520	1156.494	1155.510	1201.515	1184.489	1183.505	1219.526	1218.542	102.055	1326.513	1300.533	1283.507	3	G
12	H	1310.579	1293.553	1292.569	1338.574	1321.548	1320.564	1356.585	1355.601	**110.071**	1439.597	1413.618	1396.591	2	I
13	R	1466.680	1449.654	1448.670	1494.675	1477.649	1476.665	**1512.686**	1511.702	129.113	1538.665	**1512.686**	**1495.659**	1	V

BioTools Version 3.2 Copyright © 1999-2008 Bruker Daltonik GmbH Page -2-

Area 5. Annotated MS/MS spectrum of ion 888.452 *m*/*z*.

Spectrum Analysis Report

Sequence Name:		Parentmass:	888.452	Mass Error:	0.023	MH+ (mono):	888.429
MH+ (avg):	889.053	Threshold (a.i.):	0.000	Tolerance (Da):	0.700	Number of Peaks:	53
Above Threshold:	53	Assigned Peaks:	18	Not assigned Peaks:	35		

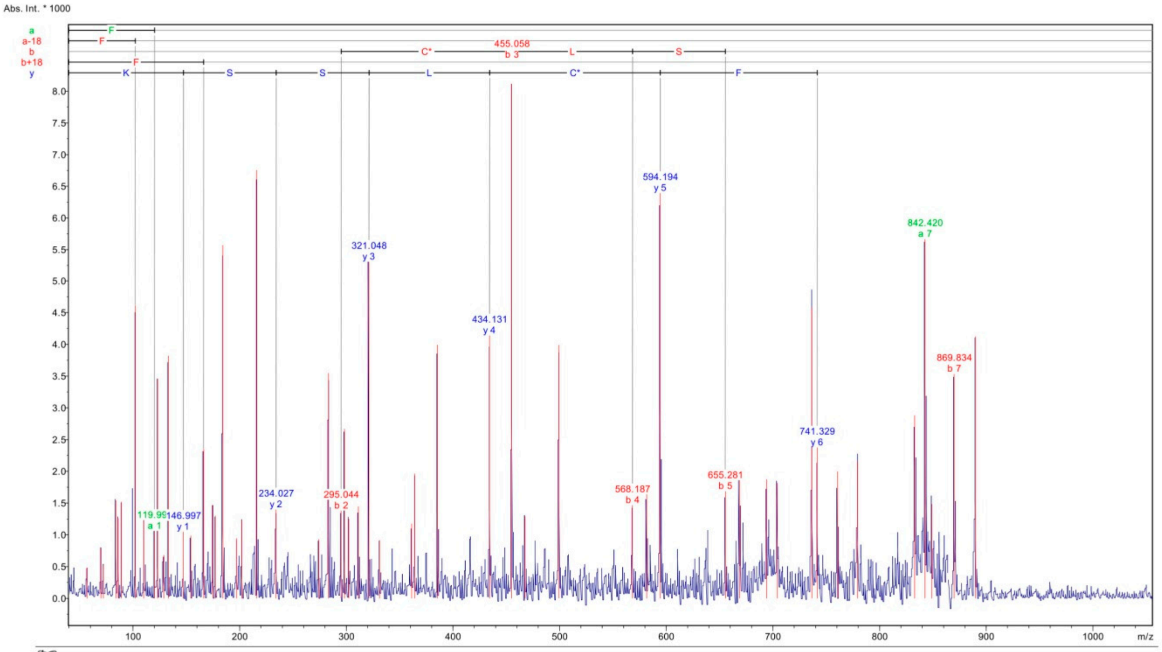

 Copyright © 1999-2008 Bruker Daltonik GmbH

Spectrum Analysis Report

Display Parameter:

Parentmass:	888.452	Mass Error:	0.023	MH+ (mono):	888.429	MH+ (avg):	889.053
Threshold (a.i.):	0.000	Tolerance (Da):	0.700	Number of Peaks:	53	Above Threshold:	53
Assigned Peaks:	18	Not assigned Peaks:	35				

Peaklist:

Peak	Mass	Intensity	Peak	Mass	Intensity	Peak	Mass	Intensity	Peak	Mass	Intensity	Peak	Mass	Intensity
1	56.902	488.413	2	69.942	795.242	3	71.986	526.068	4	83.972	1544.545	5	86.009	1261.505
6	88.965	1525.353	7	102.000	4615.877	8	109.973	1266.961	9	119.993	1061.291	10	123.020	3459.474
11	129.000	683.436	12	132.973	3822.179	13	146.997	1048.126	14	153.984	990.236	15	166.055	2349.984
16	175.014	1473.619	17	177.026	1303.520	18	184.083	5571.883	19	197.007	948.157	20	202.025	1231.880
21	216.052	6758.664	22	234.027	1400.228	23	274.017	931.482	24	283.206	3548.925	25	295.044	1380.273
26	298.168	2668.943	27	302.138	1255.318	28	311.227	1448.461	29	321.048	5308.246	30	331.043	918.732
31	361.047	1179.058	32	364.046	1966.261	33	385.274	3995.934	34	434.131	4140.731	35	455.058	8495.811
36	467.361	1294.450	37	499.343	3991.787	38	568.187	1466.639	39	581.456	1661.112	40	594.194	6395.061
41	655.281	1687.851	42	668.454	1931.467	43	694.094	1876.646	44	704.033	1820.428	45	736.480	4577.611
46	741.329	2376.846	47	760.405	2000.854	48	779.201	2152.524	49	832.711	2882.154	50	842.420	5668.875
51	848.794	1487.540	52	869.834	3536.499	53	889.653	4127.877						

Calculated Masses:
FFCLSSK 3: Carbamidomethyl (C)

N-Term.	Ion	a	a-17	a-18	b	b-17	b-18	b+18	c	i	x	y	z	C-Term.	Ion
1	F	**120.081**	103.054	**102.070**	148.076	131.049	130.065	**166.086**	165.102	**120.081**	173.092	**147.113**	130.086	7	K
2	F	267.149	250.123	249.139	**295.144**	278.118	277.134	313.155	312.171	**120.081**	260.124	**234.145**	217.118	6	S
3	C*	427.180	410.153	409.169	**455.175**	438.148	437.164	473.185	472.201	**133.043**	347.156	**321.177**	304.150	5	S
4	L	540.264	523.237	522.253	**568.259**	551.232	550.248	586.269	585.285	**86.096**	460.240	**434.261**	417.234	4	L
5	S	627.296	610.269	609.285	**655.291**	638.264	637.280	673.301	672.317	60.044	620.271	**594.292**	577.265	3	C*
6	S	714.328	697.301	696.317	742.323	725.296	724.312	**760.333**	759.349	60.044	767.339	**741.360**	724.333	2	F
7	K	**842.423**	825.396	824.412	**870.418**	853.391	852.407	888.428	887.444	101.107	914.408	888.428	871.402	1	F

 Copyright © 1999-2008 Bruker Daltonik GmbH

Area 5. Annotated MS/MS spectrum of ion 1189.656 *m*/*z*.

Spectrum Analysis Report

Sequence Name:		Parentmass:	1189.656	Mass Error:	0.016	MH+ (mono):	1189.640
MH+ (avg):	1190.439	Threshold (a.i.):	0.000	Tolerance (Da):	0.700	Number of Peaks:	44
Above Threshold:	44	Assigned Peaks:	17	Not assigned Peaks:	27		

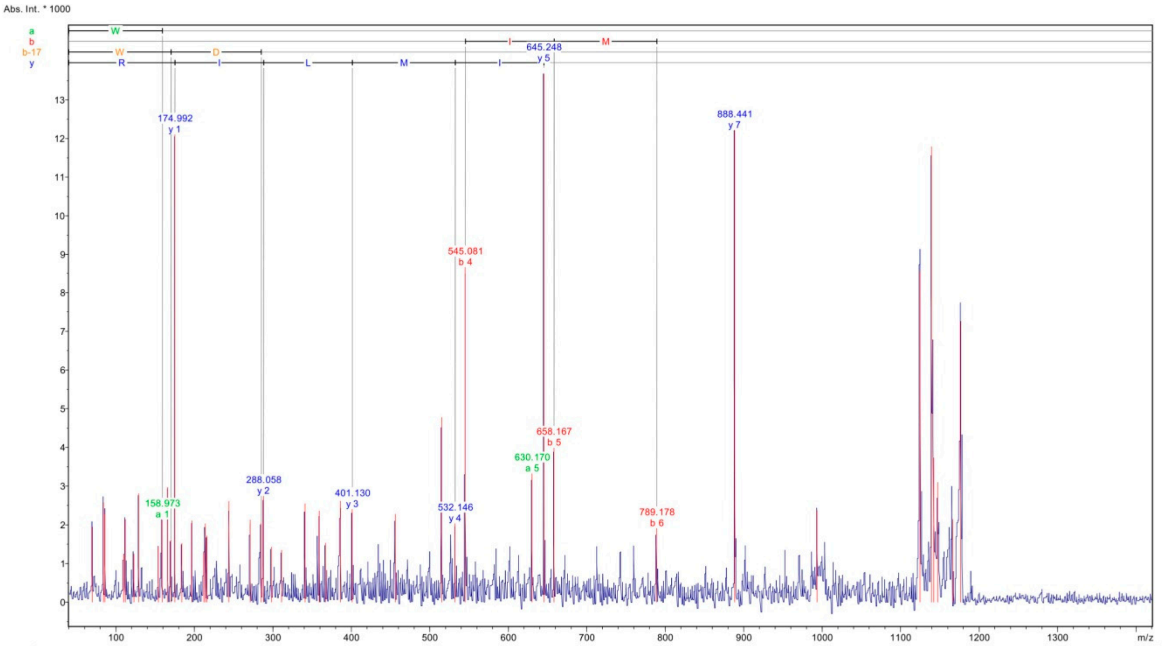

Spectrum Analysis Report

Display Parameter:

Parentmass:	1189.656	Mass Error:	0.016	MH+ (mono):	1189.640	MH+ (avg):	1190.439
Threshold (a.i.):	0.000	Tolerance (Da):	0.700	Number of Peaks:	44	Above Threshold:	44
Assigned Peaks:	17	Not assigned Peaks:	27				

Peaklist:

Peak	Mass	Intensity	Peak	Mass	Intensity	Peak	Mass	Intensity	Peak	Mass	Intensity	Peak	Mass	Intensity
1	69.988	1951.459	2	83.975	2581.839	3	86.009	2275.108	4	109.939	1227.294	5	111.972	2149.867
6	122.999	1256.430	7	128.997	2813.319	8	153.962	1444.612	9	158.973	2136.322	10	166.011	2977.716
11	169.931	1621.322	12	174.992	12107.406	13	183.988	1531.105	14	196.992	2114.026	15	211.998	1302.597
16	213.998	2036.555	17	215.989	1723.260	18	243.996	2613.610	19	271.030	2135.326	20	285.019	2055.698
21	288.058	2748.332	22	298.051	1431.459	23	311.067	1352.426	24	341.019	2551.690	25	359.018	2369.847
26	367.077	1536.193	27	386.099	2618.908	28	401.130	2404.586	29	456.090	2280.326	30	515.140	4780.260
31	532.146	2035.626	32	545.081	8669.033	33	630.170	3330.533	34	645.248	13950.409	35	658.167	3993.054
36	789.178	1913.414	37	888.441	12218.851	38	993.368	2377.892	39	1124.561	8559.726	40	1139.183	11795.402
41	1142.121	3739.024	42	1147.181	3103.887	43	1166.125	2145.961	44	1176.590	7263.730			

Calculated Masses:
WDKDIMLIR

N-Term.	Ion	a	a-17	a-18	b	b-17	b-18	b+18	c	i	x	y	z	C-Term.	Ion
1	W	**159.092**	142.065	141.081	187.087	**170.060**	169.076	205.097	204.113	**159.092**	201.098	**175.119**	158.092	9	R
2	D	274.119	257.092	256.108	302.114	**285.087**	284.103	320.124	319.140	88.039	314.182	**288.203**	**271.176**	8	I
3	K	402.214	385.187	384.203	430.208	413.182	412.198	448.219	447.235	101.107	427.266	**401.287**	384.261	7	L
4	D	517.241	500.214	499.230	**545.235**	528.209	527.225	563.246	562.262	88.039	558.307	**532.328**	**515.301**	6	M
5	I	**630.325**	613.298	612.314	**658.320**	641.293	640.309	676.330	675.346	**86.096**	671.391	**645.412**	628.385	5	I
6	M	761.365	744.339	743.355	**789.360**	772.333	771.349	807.371	806.387	104.053	786.418	760.439	743.412	4	D
7	L	874.449	857.423	856.439	902.444	885.418	884.433	920.455	919.471	**86.096**	914.513	**888.534**	871.507	3	K
8	I	987.533	970.507	969.523	1015.528	998.502	997.518	1033.539	1032.555	**86.096**	1029.540	1003.560	986.534	2	D
9	R	1143.634	1126.608	1125.624	1171.629	1154.603	1153.619	1189.640	1188.656	**129.113**	1215.619	1189.640	1172.613	1	W

Area 5. Annotated MS/MS spectrum of ion 1208.642 *m*/*z*.

Spectrum Analysis Report

Sequence Name:		Parentmass:	1208.642	Mass Error:	0.007	MH+ (mono):	1208.635
MH+ (avg):	1209.439	Threshold (a.i.):	0.000	Tolerance (Da):	0.700	Number of Peaks:	44
Above Threshold:	44	Assigned Peaks:	21	Not assigned Peaks:	23		

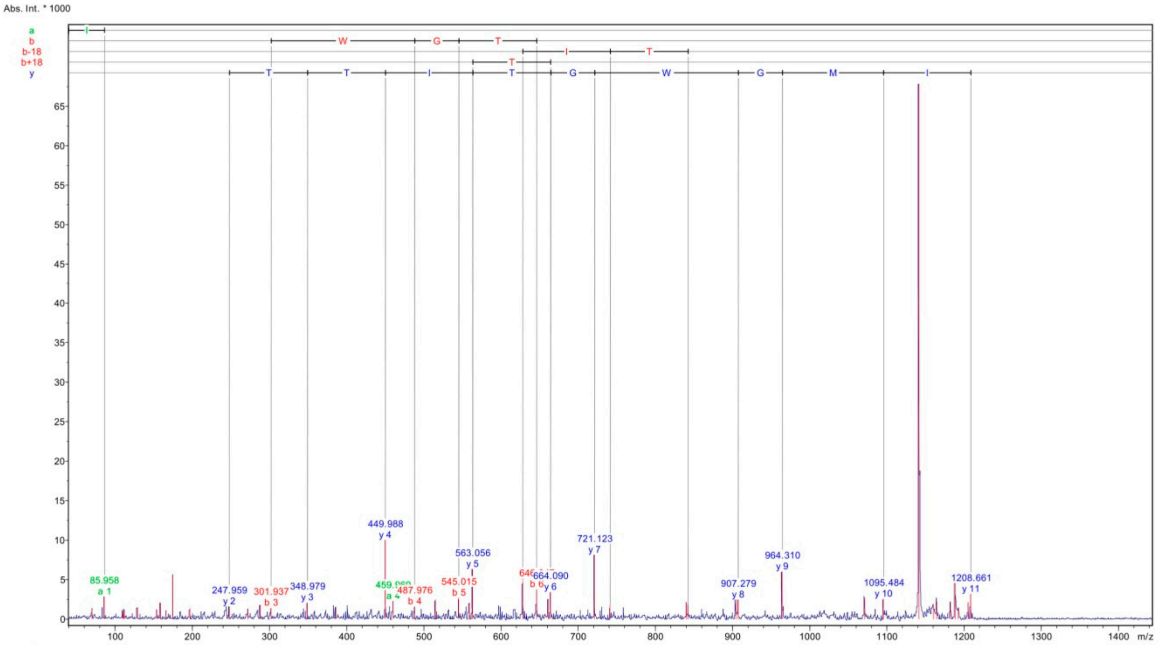

BioTools Version 3.2 Copyright © 1999-2008 Bruker Daltonik GmbH Page -1-

Spectrum Analysis Report

Display Parameter:

Parentmass:	1208.642	Mass Error:	0.007	MH+ (mono):	1208.635	MH+ (avg):	1209.439
Threshold (a.i.):	0.000	Tolerance (Da):	0.700	Number of Peaks:	44	Above Threshold:	44
Assigned Peaks:	21	Not assigned Peaks:	23				

Peaklist:

Peak	Mass	Intensity	Peak	Mass	Intensity	Peak	Mass	Intensity	Peak	Mass	Intensity	Peak	Mass	Intensity
1	69.939	1316.609	2	85.958	2853.790	3	109.899	1052.899	4	111.923	1210.909	5	128.942	1475.463
6	153.922	1205.242	7	158.929	2085.436	8	165.920	1144.857	9	174.930	5682.127	10	196.926	1382.486
11	247.959	1617.452	12	271.949	1329.827	13	287.986	1853.963	14	301.937	1448.276	15	348.979	2118.559
16	386.012	1574.154	17	449.988	10023.731	18	459.969	2304.335	19	487.976	1613.690	20	514.987	2503.822
21	545.015	2712.625	22	559.001	2089.724	23	563.056	6343.080	24	628.031	4771.806	25	646.047	3799.679
26	661.148	2584.398	27	664.090	3454.761	28	721.123	8140.831	29	741.100	1749.907	30	840.291	2163.035
31	842.167	1681.462	32	904.269	2606.545	33	907.279	2469.624	34	964.310	6035.407	35	1071.298	2751.792
36	1095.484	2585.529	37	1141.387	68295.724	38	1160.236	1999.814	39	1164.598	2224.776	40	1182.292	2274.138
41	1188.286	5800.729	42	1192.227	1383.739	43	1205.334	2192.811	44	1208.661	3180.918			

Calculated Masses:
IMGWGTITTTK

N-Term.	Ion	a	a-17	a-18	b	b-17	b-18	b+18	c	i	x	y	z	C-Term.	Ion
1	I	**86.096**	69.070	68.086	114.091	97.065	96.081	132.102	131.118	**86.096**	173.092	147.113	130.086	11	K
2	M	217.137	200.110	199.126	245.132	228.105	227.121	263.142	262.158	104.053	274.140	**248.160**	231.134	10	T
3	G	274.158	257.132	256.148	**302.153**	285.127	284.143	320.164	319.180	30.034	375.187	**349.208**	332.182	9	T
4	W	**460.238**	443.211	442.227	**488.233**	471.206	470.222	506.243	505.259	**159.092**	476.235	**450.256**	433.229	8	T
5	G	517.259	500.233	499.249	**545.254**	528.228	527.243	**563.265**	562.281	30.034	589.319	**563.340**	546.313	7	I
6	T	618.307	601.280	600.296	**646.302**	629.275	**628.291**	**664.312**	663.328	74.060	690.367	**664.388**	647.361	6	T
7	I	731.391	714.364	713.380	759.386	742.359	**741.375**	777.396	776.412	**86.096**	747.388	**721.409**	704.382	5	G
8	T	832.439	815.412	814.428	860.433	843.407	**842.423**	878.444	877.460	74.060	933.468	**907.488**	890.462	4	W
9	T	933.486	916.460	915.476	961.481	944.455	943.471	979.492	978.508	74.060	990.489	**964.510**	947.483	3	G
10	T	1034.534	1017.507	1016.523	1062.529	1045.502	1044.518	1080.539	1079.555	74.060	1121.530	**1095.550**	1078.524	2	M
11	K	1162.629	1145.602	1144.618	1190.624	1173.597	1172.613	**1208.634**	1207.650	101.107	1234.614	**1208.634**	**1191.608**	1	I

BioTools Version 3.2 Copyright © 1999-2008 Bruker Daltonik GmbH Page -2-

Area 5. Annotated MS/MS spectrum of ion 1498.790 *m*/*z*.

Spectrum Analysis Report

Sequence Name:		Parentmass:	1498.790	Mass Error:	0.119	MH+ (mono):	1498.671
MH+ (avg):	1499.590	Threshold (a.i.):	0.000	Tolerance (Da):	0.400	Number of Peaks:	25
Above Threshold:	25	Assigned Peaks:	16	Not assigned Peaks:	9		

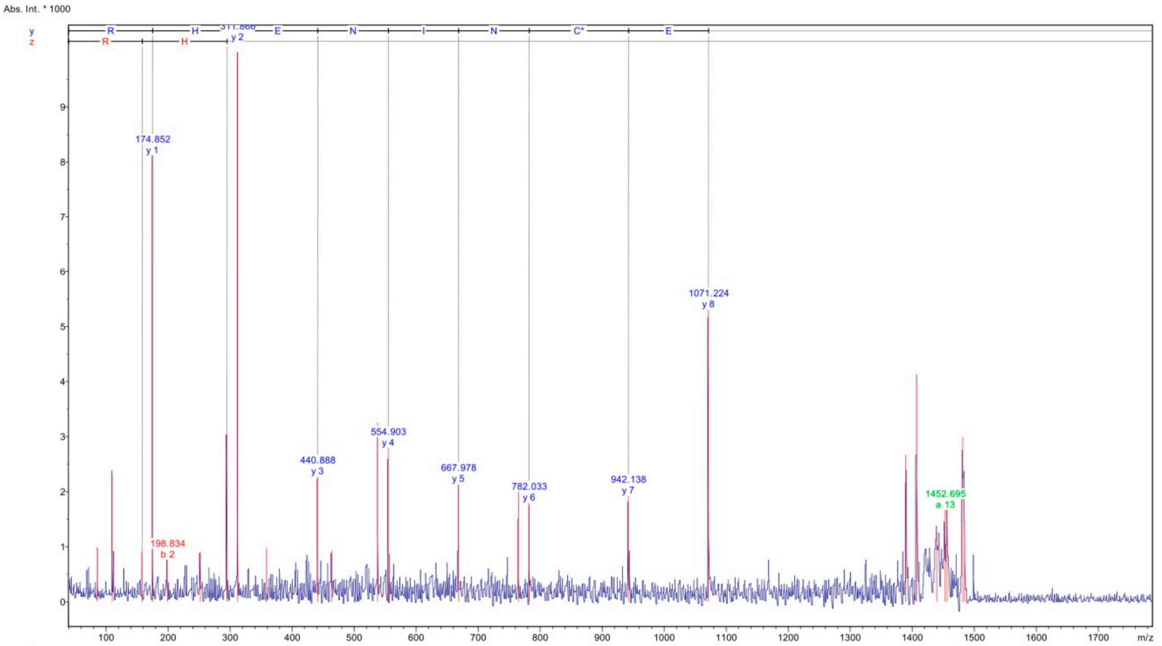

Copyright © 1999-2008 Bruker Daltonik GmbH

Spectrum Analysis Report

Display Parameter:

Parentmass:	1498.790	Mass Error:	0.119	MH+ (mono):	1498.671	MH+ (avg):	1499.590
Threshold (a.i.):	0.000	Tolerance (Da):	0.400	Number of Peaks:	25	Above Threshold:	25
Assigned Peaks:	16	Not assigned Peaks:	9				

Peaklist:

Peak	Mass	Intensity	Peak	Mass	Intensity	Peak	Mass	Intensity	Peak	Mass	Intensity	Peak	Mass	Intensity
1	85.886	965.286	2	109.840	2304.396	3	157.837	889.073	4	174.852	8119.756	5	198.834	763.869
6	251.835	911.903	7	294.851	3065.086	8	311.866	10175.752	9	358.812	986.094	10	440.888	2278.247
11	463.914	942.031	12	537.869	3256.406	13	554.903	2794.946	14	667.978	2140.914	15	764.989	2011.967
16	782.033	1800.155	17	942.138	1928.426	18	1071.224	5308.886	19	1389.488	2674.597	20	1407.110	4024.491
21	1439.512	1259.628	22	1452.695	1667.502	23	1455.973	1774.642	24	1481.239	2998.493	25	1484.227	1471.720

Calculated Masses:
VVGGDECNINEHR 7: Carbamidomethyl (C)

N-Term.	Ion	a	a-17	a-18	b	b-17	b-18	b+18	c	i	x	y	z	C-Term.	Ion
1	V	72.081	55.054	54.070	100.076	83.049	82.065	118.086	117.102	72.081	201.098	**175.119**	**158.092**	13	R
2	V	171.149	154.123	153.139	**199.144**	182.118	181.134	217.155	216.171	72.081	338.157	**312.178**	**295.151**	12	H
3	G	228.171	211.144	210.160	256.166	239.139	238.155	274.176	273.192	30.034	467.200	**441.220**	424.194	11	E
4	G	285.192	268.166	267.182	313.187	296.160	**295.176**	331.198	330.214	30.034	581.243	**555.263**	**538.237**	10	N
5	D	400.219	383.193	382.208	428.214	411.187	410.203	446.225	445.241	88.039	694.327	**668.347**	651.321	9	I
6	E	529.262	512.235	511.251	557.257	540.230	539.246	575.267	574.283	102.055	808.370	**782.390**	**765.364**	8	N
7	C*	689.292	672.266	671.282	717.287	700.261	699.277	735.298	734.314	133.043	968.400	**942.421**	925.394	7	C*
8	N	803.335	786.309	785.325	831.330	814.304	813.320	849.341	848.357	87.055	1097.443	**1071.464**	1054.437	6	E
9	I	916.419	899.393	898.409	944.414	927.388	926.404	962.425	961.441	**86.096**	1212.470	1186.491	1169.464	5	D
10	N	1030.462	1013.436	1012.452	1058.457	1041.431	1040.447	1076.468	1075.484	87.055	1269.491	1243.512	1226.485	4	G
11	E	1159.505	1142.478	1141.494	1187.500	1170.473	1169.489	1205.510	1204.526	102.055	1326.513	1300.533	1283.507	3	G
12	H	1296.564	1279.537	1278.553	1324.559	1307.532	1306.548	1342.569	1341.585	**110.071**	1425.581	1399.602	1382.575	2	V
13	R	**1452.665**	1435.638	1434.654	1480.660	1463.633	1462.649	1498.670	1497.686	129.113	1524.650	1498.670	1481.644	1	V

Copyright © 1999-2008 Bruker Daltonik GmbH

Area 5. Annotated MS/MS spectrum of ion 1592.776 *m*/*z*.

Spectrum Analysis Report

Sequence Name:		Parentmass:	1592.776	Mass Error:	-0.002	MH+ (mono):	1592.778
MH+ (avg):	1593.850	Threshold (a.i.):	0.000	Tolerance (Da):	0.700	Number of Peaks:	32
Above Threshold:	32	Assigned Peaks:	25	Not assigned Peaks:	7		

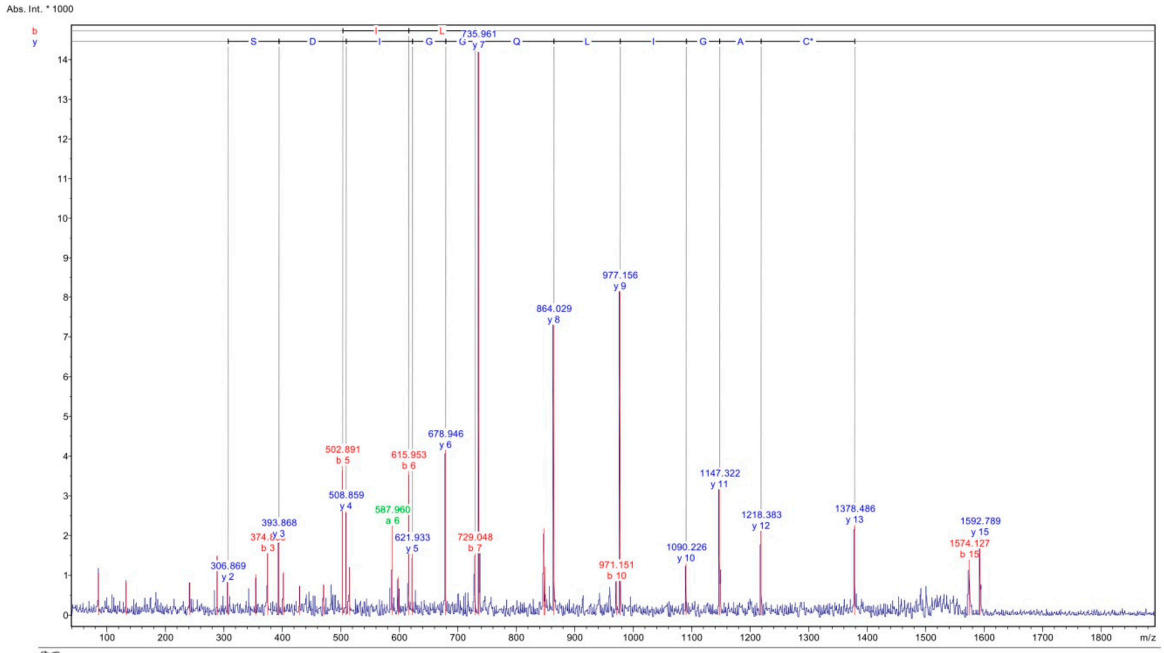

BioTools Version 3.2 Copyright © 1999-2008 Bruker Daltonik GmbH Page -1-

Spectrum Analysis Report

Display Parameter:

Parentmass:	1592.776	Mass Error:	-0.002	MH+ (mono):	1592.778	MH+ (avg):	1593.850
Threshold (a.i.):	0.000	Tolerance (Da):	0.700	Number of Peaks:	32	Above Threshold:	32
Assigned Peaks:	25	Not assigned Peaks:	7				

Peaklist:

Peak	Mass	Intensity	Peak	Mass	Intensity	Peak	Mass	Intensity	Peak	Mass	Intensity	Peak	Mass	Intensity
1	85.905	1063.412	2	132.847	830.626	3	241.891	817.863	4	288.827	1496.918	5	306.869	829.366
6	354.904	1029.595	7	374.863	1551.329	8	393.868	1915.657	9	401.873	1086.438	10	429.880	743.303
11	470.889	757.054	12	502.891	3759.363	13	508.859	2611.117	14	514.891	1165.983	15	587.960	2245.748
16	597.986	969.469	17	615.953	3623.859	18	621.933	1545.009	19	678.946	4151.695	20	729.048	1554.907
21	735.961	14240.065	22	847.039	2181.152	23	848.956	1222.166	24	864.029	7304.431	25	971.151	855.766
26	977.156	8147.261	27	1090.226	1301.177	28	1147.322	3160.719	29	1218.383	2116.883	30	1378.486	2272.304
31	1574.127	1396.536	32	1592.789	1961.810									

Calculated Masses:
TLCAGILQGGIDSCK 3: Carbamidomethyl (C) 14: Carbamidomethyl (C)

N-Term.	Ion	a	a-17	a-18	b	b-17	b-18	b+18	c	i	x	y	z	C-Term.	Ion
1	T	74.060	57.033	56.049	102.055	85.028	84.044	120.066	119.082	74.060	173.092	147.113	130.086	15	K
2	L	187.144	170.118	169.134	215.139	198.112	197.128	233.150	232.166	**86.096**	333.123	**307.143**	290.117	14	C*
3	C*	347.175	330.148	329.164	**375.170**	358.143	357.159	**393.180**	392.196	**133.043**	420.155	**394.175**	377.149	13	S
4	A	418.212	**401.185**	400.201	446.207	429.180	428.196	464.217	463.233	44.049	535.182	**509.202**	492.176	12	D
5	G	475.233	458.207	457.223	**503.228**	486.202	485.218	521.239	520.255	30.034	648.266	**622.286**	605.260	11	I
6	I	**588.317**	571.291	570.307	**616.312**	599.286	**598.302**	634.323	633.339	**86.096**	705.287	**679.308**	662.281	10	G
7	L	701.401	684.375	683.391	**729.396**	712.370	711.386	747.407	746.423	**86.096**	762.309	**736.329**	719.303	9	G
8	Q	829.460	812.433	811.449	857.455	840.428	839.444	875.466	874.481	101.071	890.367	**864.388**	**847.361**	8	Q
9	G	886.481	869.455	868.471	914.476	897.450	896.466	932.487	931.503	30.034	1003.451	**977.472**	960.446	7	L
10	G	943.503	926.476	925.492	**971.498**	954.471	953.487	989.508	988.524	30.034	1116.535	**1090.556**	1073.530	6	I
11	I	1056.587	1039.560	1038.576	1084.582	1067.555	1066.571	1102.593	1101.608	**86.096**	1173.557	**1147.578**	1130.551	5	G
12	D	1171.614	1154.587	1153.603	1199.609	1182.582	1181.598	1217.619	1216.635	88.039	1244.594	**1218.615**	1201.588	4	A
13	S	1258.646	1241.619	1240.635	1286.641	1269.614	1268.630	1304.651	1303.667	60.044	1404.625	**1378.645**	1361.619	3	C*
14	C*	1418.677	1401.650	1400.666	1446.672	1429.645	1428.661	1464.682	1463.698	**133.043**	1517.709	1491.729	1474.703	2	L
15	K	1546.772	1529.745	1528.761	**1574.767**	1557.740	1556.756	**1592.777**	1591.793	101.107	1618.756	**1592.777**	1575.751	1	T

BioTools Version 3.2 Copyright © 1999-2008 Bruker Daltonik GmbH Page -2-

Area 6. Annotated MS/MS spectrum of ion 1537.689 *m*/*z*.

Spectrum Analysis Report

Sequence Name:		Parentmass:	1537.689	Mass Error:	0.015	MH+ (mono):	1537.675
MH+ (avg):	1538.664	Threshold (a.i.):	0.000	Tolerance (Da):	0.700	Number of Peaks:	42
Above Threshold:	42	Assigned Peaks:	22	Not assigned Peaks:	20		

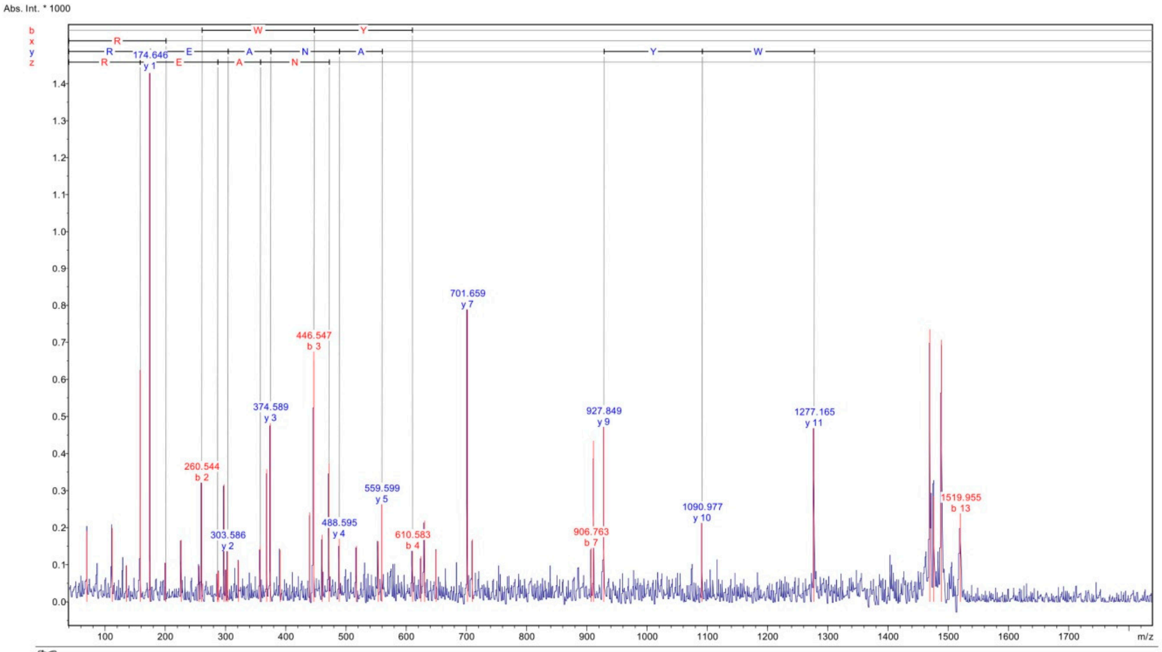

Copyright © 1999-2008 Bruker Daltonik GmbH

Spectrum Analysis Report

Display Parameter:

Parentmass:	1537.689	Mass Error:	0.015	MH+ (mono):	1537.675	MH+ (avg):	1538.664
Threshold (a.i.):	0.000	Tolerance (Da):	0.700	Number of Peaks:	42	Above Threshold:	42
Assigned Peaks:	22	Not assigned Peaks:	20				

Peaklist:

Peak	Mass	Intensity	Peak	Mass	Intensity	Peak	Mass	Intensity	Peak	Mass	Intensity	Peak	Mass	Intensity
1	69.736	191.706	2	111.712	199.027	3	135.665	98.715	4	158.634	624.835	5	174.646	1434.016
6	200.552	105.650	7	226.596	166.881	8	256.613	97.210	9	260.544	321.461	10	286.561	79.908
11	288.547	80.255	12	297.570	317.376	13	300.565	86.750	14	303.586	136.642	15	321.596	110.425
16	357.551	150.069	17	368.564	357.901	18	374.589	481.942	19	390.577	140.414	20	439.563	241.021
21	446.547	674.706	22	460.571	181.084	23	471.563	373.817	24	488.595	169.667	25	517.540	150.616
26	553.548	163.703	27	559.599	262.805	28	610.583	137.552	29	624.608	123.420	30	630.607	218.757
31	649.632	138.638	32	701.659	788.346	33	709.640	168.762	34	906.763	145.267	35	910.778	434.375
36	927.849	471.356	37	1090.977	212.287	38	1277.165	468.729	39	1469.146	734.927	40	1475.690	286.271
41	1488.484	707.030	42	1519.955	237.926									

Calculated Masses:
MEWYPEAAANAER

N-Term.	Ion	a	a-17	a-18	b	b-17	b-18	b+18	c	i	x	y	z	C-Term.	Ion
1	M	104.053	87.026	86.042	132.048	115.021	114.037	150.058	149.074	104.053	**201.098**	**175.119**	**158.092**	13	R
2	E	233.095	216.069	215.085	**261.090**	244.064	243.080	279.101	278.117	102.055	330.141	**304.162**	**287.135**	12	E
3	W	419.175	402.148	401.164	**447.170**	430.143	429.159	465.180	464.196	**159.092**	401.178	**375.199**	**358.172**	11	A
4	Y	582.238	565.212	564.228	**610.233**	593.206	592.222	628.244	627.260	**136.076**	515.221	**489.242**	**472.215**	10	N
5	P	679.291	662.264	661.280	707.286	690.259	689.275	725.296	724.312	**70.065**	586.258	**560.279**	543.252	9	A
6	E	808.333	791.307	790.323	836.328	819.302	818.318	854.339	853.355	102.055	657.295	631.316	614.289	8	A
7	A	879.371	862.344	861.360	**907.365**	890.339	889.355	925.376	924.392	44.049	728.332	**702.353**	685.326	7	A
8	A	950.408	933.381	932.397	978.403	961.376	960.392	996.413	995.429	44.049	857.375	831.396	814.369	6	E
9	A	1021.445	1004.418	1003.434	1049.440	1032.413	1031.429	1067.450	1066.466	44.049	954.428	**928.448**	**911.422**	5	P
10	N	1135.488	1118.461	1117.477	1163.483	1146.456	1145.472	1181.493	1180.509	87.055	1117.491	**1091.512**	1074.485	4	Y
11	A	1206.525	1189.498	1188.514	1234.520	1217.493	1216.509	1252.530	1251.546	44.049	1303.570	**1277.591**	1260.564	3	W
12	E	1335.567	1318.541	1317.557	1363.562	1346.536	1345.552	1381.573	1380.589	102.055	1432.613	1406.634	1389.607	2	E
13	R	1491.669	1474.642	1473.658	**1519.663**	1502.637	1501.653	1537.674	1536.690	129.113	1563.653	1537.674	**1520.647**	1	M

Copyright © 1999-2008 Bruker Daltonik GmbH

Area 6. Annotated MS/MS spectrum of ion 1919.083 *m*/*z*.

Spectrum Analysis Report

Sequence Name:		Parentmass:	1919.083	Mass Error:	0.048	MH+ (mono):	1919.035
MH+ (avg):	1920.155	Threshold (a.i.):	0.000	Tolerance (Da):	0.800	Number of Peaks:	64
Above Threshold:	64	Assigned Peaks:	47	Not assigned Peaks:	17		

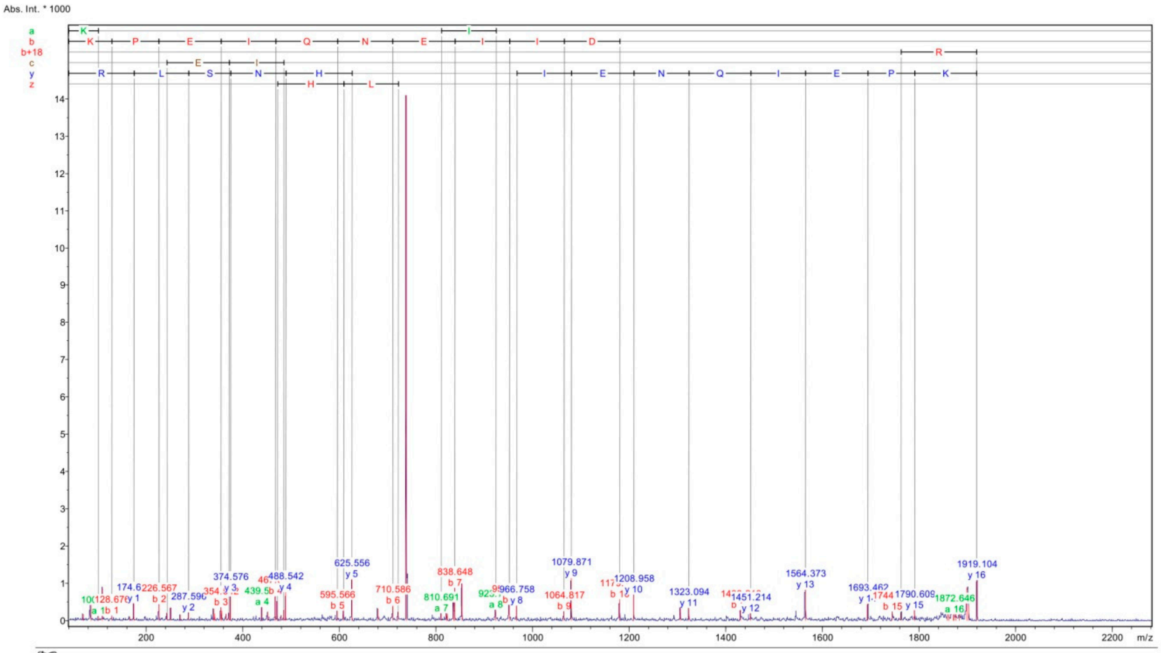

BioTools Version 3.2 Copyright © 1999-2008 Bruker Daltonik GmbH Page -1-

Spectrum Analysis Report

Display Parameter:

Parentmass:	1919.083	Mass Error:	0.048	MH+ (mono):	1919.035	MH+ (avg):	1920.155
Threshold (a.i.):	0.000	Tolerance (Da):	0.800	Number of Peaks:	64	Above Threshold:	64
Assigned Peaks:	47	Not assigned Peaks:	17				

Peaklist:

Peak	Mass	Intensity	Peak	Mass	Intensity	Peak	Mass	Intensity	Peak	Mass	Intensity	Peak	Mass	Intensity
1	69.743	183.286	2	85.737	547.237	3	100.669	124.960	4	109.657	862.611	5	128.676	133.026
6	165.593	113.922	7	174.630	461.209	8	226.567	439.126	9	243.539	204.924	10	250.562	350.474
11	270.587	163.836	12	287.596	219.746	13	339.550	321.415	14	341.565	191.932	15	354.542	357.949
16	356.539	271.400	17	365.528	184.630	18	371.485	216.771	19	374.576	741.663	20	439.571	366.190
21	451.549	247.247	22	467.541	659.778	23	471.535	537.121	24	479.497	150.778	25	484.530	303.255
26	488.542	760.584	27	595.566	264.610	28	608.550	451.697	29	625.556	1111.603	30	679.580	331.562
31	710.586	408.518	32	721.596	246.278	33	738.618	14315.169	34	810.691	193.603	35	821.572	195.225
36	823.631	189.622	37	836.619	729.155	38	838.648	878.129	39	853.648	994.284	40	923.726	292.645
41	951.750	422.505	42	966.758	404.363	43	1064.817	250.717	44	1079.871	1141.066	45	1179.903	573.248
46	1208.958	698.111	47	1306.034	356.315	48	1323.094	335.478	49	1430.218	286.163	50	1451.214	193.102
51	1564.373	830.713	52	1693.462	453.782	53	1744.548	238.599	54	1762.588	205.288	55	1790.609	276.629
56	1858.073	273.107	57	1861.462	162.332	58	1870.262	168.108	59	1872.646	167.528	60	1876.000	187.942
61	1883.316	162.279	62	1899.004	898.076	63	1902.249	410.830	64	1919.104	1082.336			

Calculated Masses:
KPEIQNEIIDLHNSLR

N-Term.	Ion	a	a-17	a-18	b	b-17	b-18	b+18	c	i	x	y	z	C-Term.	Ion
1	K	**101.107**	84.081	83.097	**129.102**	112.076	111.092	147.113	146.129	**101.107**	201.098	**175.119**	158.092	16	R
2	P	198.160	181.134	180.150	**226.155**	209.128	208.144	**244.166**	**243.182**	**70.065**	314.182	**288.203**	**271.176**	15	L
3	E	327.203	310.176	309.192	**355.198**	338.171	337.187	373.208	**372.224**	102.055	401.214	**375.235**	358.208	14	S
4	I	**440.287**	423.260	422.276	**468.282**	**451.255**	450.271	486.292	**485.308**	**86.096**	515.257	**489.278**	**472.251**	13	N
5	Q	568.345	551.319	550.335	**596.340**	579.314	578.330	614.351	613.367	**101.071**	652.316	**626.337**	**609.310**	12	H
6	N	682.388	665.362	664.378	**710.383**	693.357	692.373	728.394	727.410	87.055	765.400	739.421	**722.394**	11	L
7	E	**811.431**	794.404	793.420	**839.426**	822.399	**821.415**	857.436	856.452	102.055	880.427	854.448	837.421	10	D
8	I	**924.515**	907.488	906.504	**952.510**	935.483	934.499	970.520	969.536	**86.096**	993.511	**967.532**	950.505	9	I
9	I	1037.599	1020.572	1019.588	**1065.594**	1048.567	1047.583	1083.604	1082.620	**86.096**	1106.595	**1080.616**	1063.589	8	I
10	D	1152.626	1135.599	1134.615	**1180.621**	1163.594	1162.610	1198.631	1197.647	88.039	1235.638	**1209.659**	1192.632	7	E
11	L	1265.710	1248.683	1247.699	1293.705	1276.678	1275.694	1311.715	1310.731	**86.096**	1349.681	**1323.702**	**1306.675**	6	N
12	H	1402.769	1385.742	1384.758	**1430.764**	1413.737	1412.753	1448.774	1447.790	**110.071**	1477.739	**1451.760**	1434.734	5	Q
13	N	1516.812	1499.785	1498.801	1544.807	1527.780	1526.796	1562.817	1561.833	87.055	1590.823	**1564.844**	1547.818	4	I
14	S	1603.844	1586.817	1585.833	1631.839	1614.812	1613.828	1649.849	1648.865	60.044	1719.866	**1693.887**	1676.860	3	E
15	L	1716.928	1699.901	1698.917	**1744.923**	1727.896	1726.912	**1762.933**	**1761.949**	**86.096**	1816.919	**1790.940**	1773.913	2	P
16	R	**1873.029**	1856.002	1855.018	1901.024	**1883.997**	**1883.013**	**1919.034**	1918.050	**129.113**	1945.014	**1919.034**	**1902.008**	1	K

BioTools Version 3.2 Copyright © 1999-2008 Bruker Daltonik GmbH Page -2-

Area 6. Annotated MS spectrum.

Spectrum Analysis Report

Sequence Name:	Cysteine-rich venom protein OS=Vipera berus PE=1 SV=1 CRVP_VIPBE					Parentmass:	
Mass Error:		MH+ (mono):	1.008	MH+ (avg):	1.008	Threshold (a.i.):	0.000
Tolerance (Da):	0.500	Number of Peaks:	15	Above Threshold:		Assigned Peaks:	
Not assigned Peaks:							

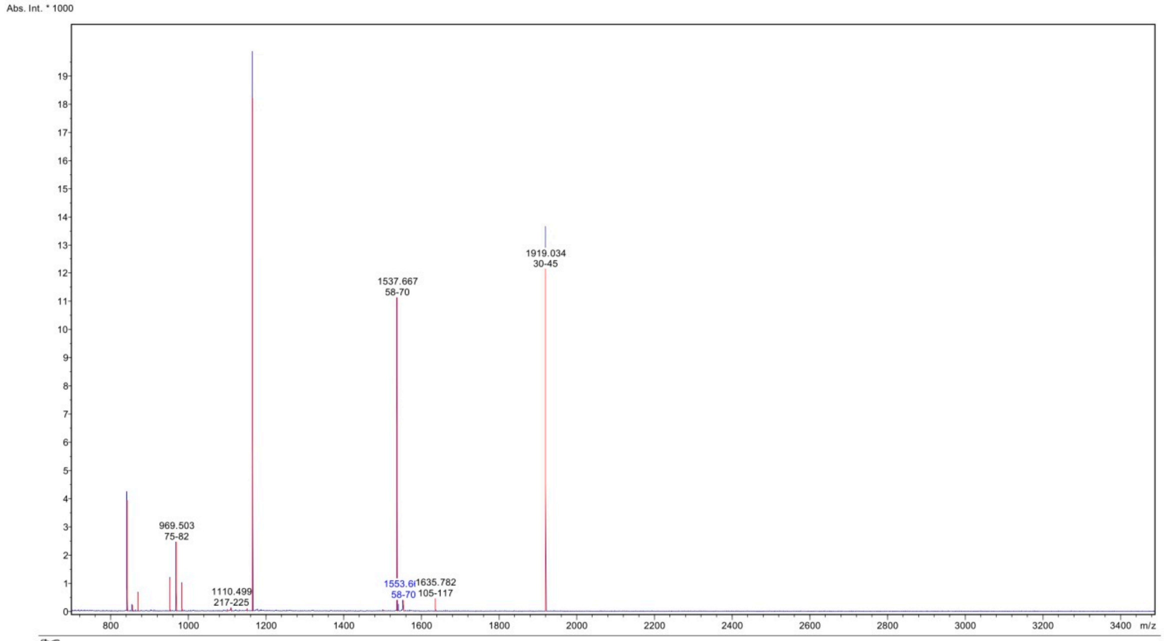

BioTools Version 3.2 Copyright © 1999-2008 Bruker Daltonik GmbH Page -1-

Spectrum Analysis Report

Sequence data:

Cysteine-rich venom protein OS=Vipera berus PE=1 SV=1 CRVP_VIPBE

Intensity Coverage:	51.3 % (26768 cnts)	Sequence Coverage MS:	24.7%
Sequence Coverage MS/MS:	0.0%	pI (isoelectric point):	6.4

```
        10         20         30         40         50         60         70         80         90        100        110        120        130        140
MIAFLVLPIL AAVLQQSSGN VDFDSESPRK PEIQNEIIDL HNSLRRSVNP TASNMLKMEW YPEAAANAER WAFRCILSHS PRDSRVIGGI KCGENIYMST SPMKWTAIIH EWHGEEKDFV YGQGASPANA VVGHYTQIVW
       150        160        170        180        190        200        210        220        230        240
YKSYRSGCAA AYCPSSEYKY FYVCQYCPAG NMQGKTATPY TSGPPCGDCP SACDNGLCTN PCTHEDKFTN CKDLVKQGCN NNYLKTNCPA SCSCHNEII
```

Display Parameter:

Sequence Name:	Cysteine-rich venom protein OS=Vipera berus PE=1 SV=1 CRVP_VIPBE					MH+ (mono):	1.008
MH+ (avg):	1.008	Threshold (a.i.):	0.000	Tolerance (Da):	0.500	Number of Peaks:	15

Peaklist:

Peak	Mass	Intensity	Peak	Mass	Intensity	Peak	Mass	Intensity	Peak	Mass	Intensity	Peak	Mass	Intensity
1	842.515	3945.359	2	856.532	233.056	3	870.546	651.165	4	952.473	1166.092	5	969.503	2475.883
6	983.518	984.923	7	1100.545	82.000	8	1110.499	137.000	9	1151.610	108.000	10	1165.519	18221.673
11	1502.510	44.000	12	1537.667	11131.574	13	1553.667	413.301	14	1635.782	470.947	15	1919.034	12139.459

BioTools Version 3.2 Copyright © 1999-2008 Bruker Daltonik GmbH Page -2-

Area 7. Annotated MS/MS spectrum of ion 1352.702 *m/z*.

Spectrum Analysis Report

Sequence Name:		Parentmass:	1352.702	Mass Error:	-0.046	MH+ (mono):	1352.747
MH+ (avg):	1353.617	Threshold (a.i.):	0.000	Tolerance (Da):	0.700	Number of Peaks:	77
Above Threshold:	77	Assigned Peaks:	38	Not assigned Peaks:	39		

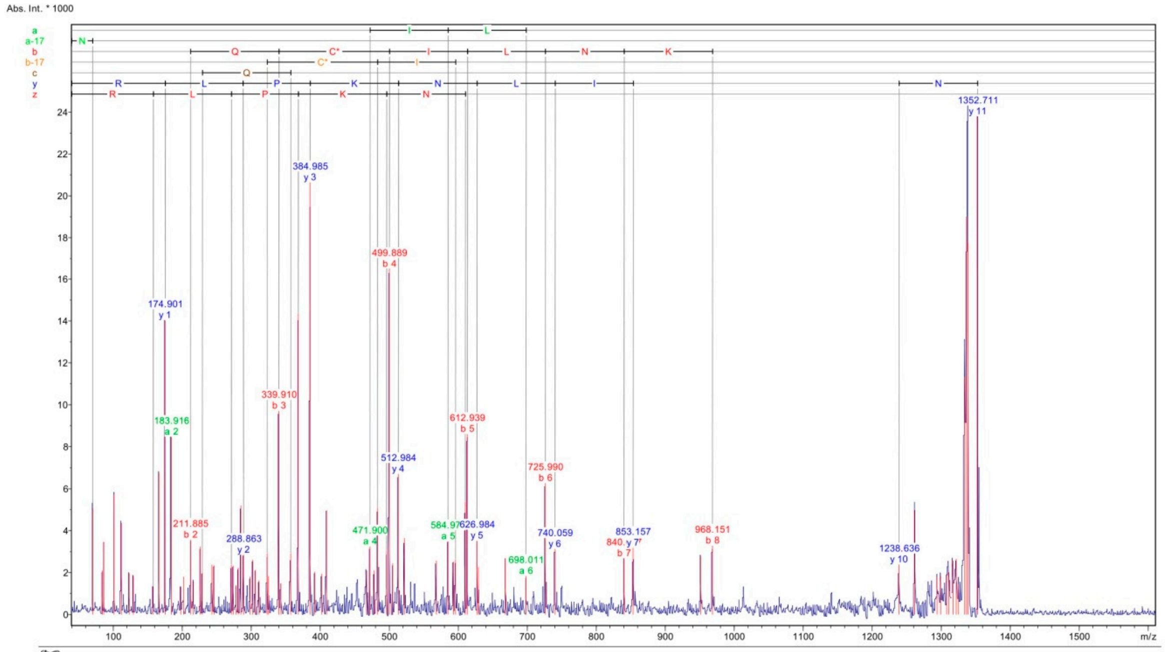

BioTools Version 3.2 Copyright © 1999-2008 Bruker Daltonik GmbH Page -1-

Spectrum Analysis Report

Display Parameter:

Parentmass:	1352.702	Mass Error:	-0.046	MH+ (mono):	1352.747	MH+ (avg):	1353.617
Threshold (a.i.):	0.000	Tolerance (Da):	0.700	Number of Peaks:	77	Above Threshold:	77
Assigned Peaks:	38	Not assigned Peaks:	39				

Peaklist:

Peak	Mass	Intensity	Peak	Mass	Intensity	Peak	Mass	Intensity	Peak	Mass	Intensity	Peak	Mass	Intensity
1	69.910	5065.252	2	83.872	2102.419	3	85.917	3457.762	4	100.895	5758.177	5	111.885	4371.352
6	122.878	1974.106	7	128.902	1851.665	8	157.876	1329.482	9	165.920	6765.764	10	174.901	14039.179
11	183.916	8476.028	12	197.881	1342.141	13	201.866	1805.846	14	211.885	3567.100	15	215.872	1576.102
16	225.896	3247.634	17	228.897	2012.625	18	242.878	2379.322	19	245.892	2348.402	20	270.923	2374.222
21	273.887	2327.593	22	277.870	1402.269	23	280.901	2241.263	24	284.930	5205.459	25	288.863	2848.383
26	297.926	1804.046	27	301.948	2619.556	28	305.868	2137.419	29	310.960	1628.405	30	322.888	2925.059
31	324.958	1829.386	32	339.910	9718.122	33	342.921	1495.028	34	356.924	2905.438	35	367.966	14354.929
36	384.985	20649.534	37	391.896	2055.676	38	401.915	1955.561	39	408.913	4974.402	40	467.060	2099.741
41	471.900	3247.470	42	477.964	2110.729	43	482.893	5106.122	44	495.973	3065.326	45	499.889	16508.671
46	504.931	2442.597	47	512.984	6706.245	48	521.951	3648.397	49	567.992	2579.260	50	584.971	3495.130
51	592.968	2531.506	52	595.918	2629.541	53	609.987	5385.366	54	612.939	8611.992	55	626.984	3522.573
56	628.961	2267.172	57	668.150	2673.040	58	698.011	1835.941	59	725.990	6273.565	60	740.059	3126.003
61	840.047	2679.019	62	853.157	3181.052	63	951.138	2795.373	64	968.151	3284.891	65	1238.636	2388.804
66	1261.676	4978.990	67	1293.612	1808.194	68	1298.874	1837.302	69	1307.571	1951.766	70	1310.845	2023.643
71	1316.960	2578.750	72	1321.573	2655.980	73	1324.875	1475.611	74	1333.454	11352.822	75	1336.458	19014.176
76	1338.504	17785.474	77	1352.711	23800.547									

Calculated Masses:
NPQCILNKPLR 4: Carbamidomethyl (C)

N-Term.	Ion	a	a-17	a-18	b	b-17	b-18	b+18	c	i	x	y	z	C-Term.	Ion
1	N	87.055	**70.029**	69.045	115.050	98.024	97.040	133.061	132.077	87.055	201.098	**175.119**	**158.092**	11	R
2	P	**184.108**	167.082	**166.097**	**212.103**	195.076	194.092	230.114	**229.130**	**70.065**	314.182	**288.203**	**271.176**	10	L
3	Q	312.167	295.140	294.156	**340.162**	**323.135**	322.151	358.172	**357.188**	**101.071**	411.235	**385.256**	**368.229**	9	P
4	C*	**472.197**	455.171	454.187	**500.192**	**483.166**	482.182	518.203	517.219	133.043	539.330	**513.351**	**496.324**	8	K
5	I	**585.281**	**568.255**	567.271	**613.276**	**596.250**	**595.266**	631.287	630.303	**86.096**	653.373	**627.394**	**610.367**	7	N
6	L	**698.365**	681.339	680.355	**726.360**	709.334	708.350	744.371	743.387	**86.096**	766.457	**740.478**	723.451	6	L
7	N	812.408	795.382	794.398	**840.403**	823.377	822.393	858.414	857.430	87.055	879.541	**853.562**	836.535	5	I
8	K	940.503	923.477	922.493	**968.498**	**951.472**	**950.488**	986.509	985.525	**101.107**	1039.572	1013.592	996.566	4	C*
9	P	1037.556	1020.530	1019.545	1065.551	1048.524	1047.540	1083.562	1082.578	**70.065**	1167.630	1141.651	1124.624	3	Q
10	L	1150.640	1133.614	1132.630	1178.635	1161.608	1160.624	1196.646	1195.662	**86.096**	1264.683	**1238.704**	1221.677	2	P
11	R	1306.741	1289.715	1288.731	1334.736	1317.710	**1316.726**	**1352.747**	1351.763	**129.113**	1378.726	**1352.747**	1335.720	1	N

BioTools Version 3.2 Copyright © 1999-2008 Bruker Daltonik GmbH Page -2-

Area 8. Annotated MS/MS spectrum of ion 906.470 *m*/*z*.

Spectrum Analysis Report

Sequence Name:		Parentmass:	906.470	Mass Error:	0.074	MH+ (mono):	906.396
MH+ (avg):	906.918	Threshold (a.i.):	0.000	Tolerance (Da):	0.700	Number of Peaks:	36
Above Threshold:	36	Assigned Peaks:	20	Not assigned Peaks:	16		

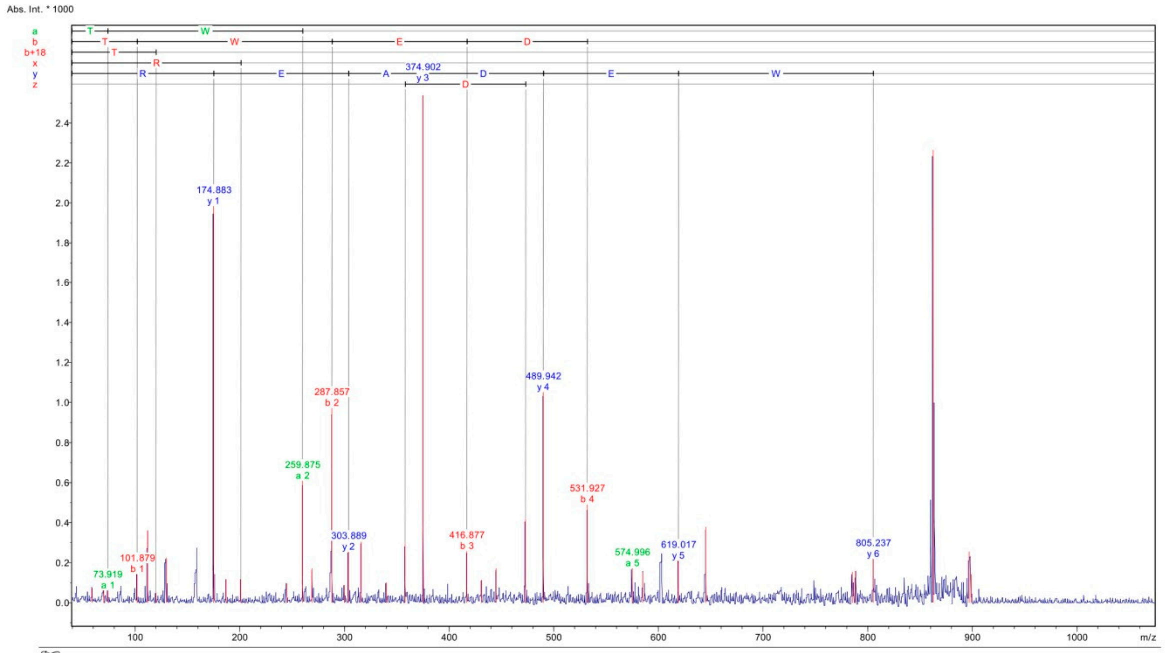

Copyright © 1999-2008 Bruker Daltonik GmbH

Spectrum Analysis Report

Display Parameter:

Parentmass:	906.470	Mass Error:	0.074	MH+ (mono):	906.396	MH+ (avg):	906.918
Threshold (a.i.):	0.000	Tolerance (Da):	0.700	Number of Peaks:	36	Above Threshold:	36
Assigned Peaks:	20	Not assigned Peaks:	16				

Peaklist:

Peak	Mass	Intensity	Peak	Mass	Intensity	Peak	Mass	Intensity	Peak	Mass	Intensity	Peak	Mass	Intensity
1	58.919	73.528	2	69.922	119.166	3	73.919	61.187	4	101.879	143.001	5	111.903	362.691
6	119.914	48.501	7	129.865	225.340	8	174.883	1984.574	9	186.853	117.581	10	200.847	119.080
11	244.825	96.662	12	259.875	608.640	13	268.851	171.612	14	287.857	972.167	15	299.856	89.139
16	303.889	254.334	17	315.845	304.714	18	339.896	101.396	19	357.875	293.583	20	374.902	2600.803
21	416.877	257.521	22	430.889	114.263	23	444.875	171.333	24	472.912	417.532	25	489.942	1051.680
26	531.927	490.850	27	574.996	172.049	28	585.032	152.353	29	619.017	209.573	30	645.034	378.852
31	785.239	153.821	32	788.156	160.231	33	805.237	217.994	34	862.402	2265.972	35	896.831	255.080
36	898.793	143.110												

Calculated Masses:
TWEDAER

N-Term.	Ion	a	a-17	a-18	b	b-17	b-18	b+18	c	i	x	y	z	C-Term.	Ion
1	T	**74.060**	57.033	56.049	**102.055**	85.028	84.044	**120.066**	119.082	**74.060**	**201.098**	**175.119**	158.092	7	R
2	W	**260.139**	243.113	242.129	**288.134**	271.108	270.124	306.145	305.161	159.092	330.141	**304.162**	287.135	6	E
3	E	389.182	372.155	371.171	**417.177**	400.150	399.166	435.187	434.203	**102.055**	401.178	**375.199**	**358.172**	5	A
4	D	504.209	487.182	486.198	**532.204**	515.177	514.193	550.214	549.230	88.039	516.205	**490.226**	**473.199**	4	D
5	A	**575.246**	558.219	557.235	603.241	586.214	**585.230**	621.251	620.267	44.049	**645.247**	**619.268**	602.242	3	E
6	E	704.289	687.262	686.278	732.284	715.257	714.273	750.294	749.310	**102.055**	831.327	**805.348**	**788.321**	2	W
7	R	860.390	843.363	842.379	888.385	871.358	870.374	906.395	905.411	129.113	932.374	906.395	889.369	1	T

Copyright © 1999-2008 Bruker Daltonik GmbH

Area 8. Annotated MS/MS spectrum of ion 1033.604 *m*/*z*.

Spectrum Analysis Report

Sequence Name:		Parentmass:	1033.604	Mass Error:	0.097	MH+ (mono):	1033.507
MH+ (avg):	1034.106	Threshold (a.i.):	0.000	Tolerance (Da):	0.700	Number of Peaks:	57
Above Threshold:	57	Assigned Peaks:	32	Not assigned Peaks:	25		

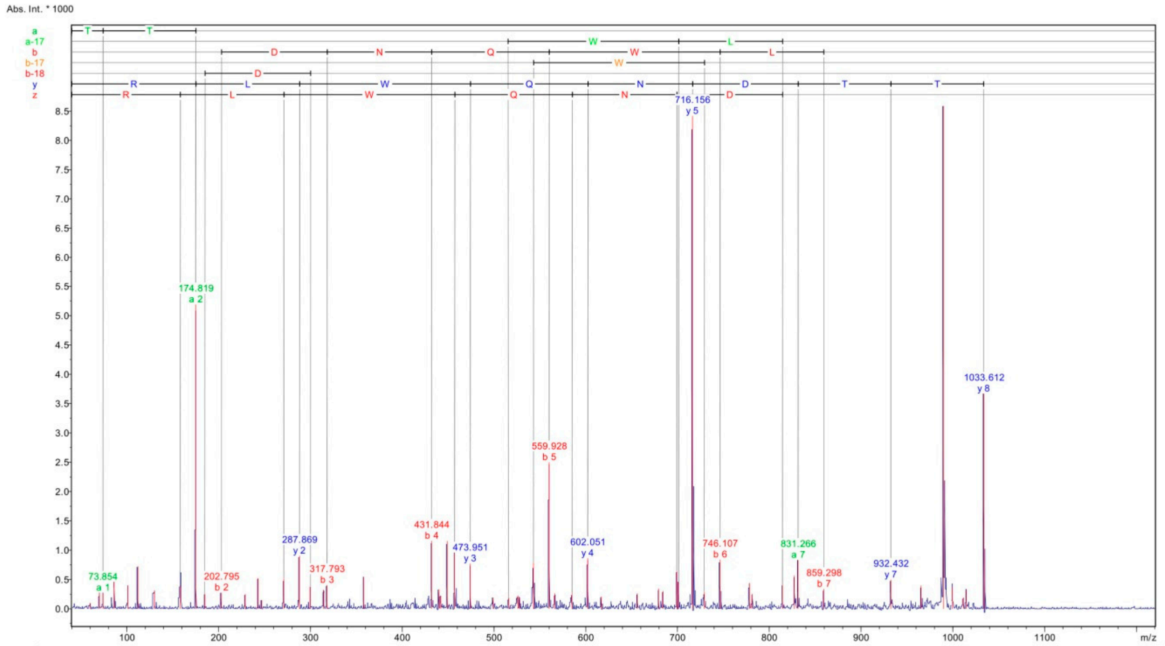

Copyright © 1999-2008 Bruker Daltonik GmbH

Spectrum Analysis Report

Display Parameter:

Parentmass:	1033.604	Mass Error:	0.097	MH+ (mono):	1033.507	MH+ (avg):	1034.106
Threshold (a.i.):	0.000	Tolerance (Da):	0.700	Number of Peaks:	57	Above Threshold:	57
Assigned Peaks:	32	Not assigned Peaks:	25				

Peaklist:

Peak	Mass	Intensity	Peak	Mass	Intensity	Peak	Mass	Intensity	Peak	Mass	Intensity	Peak	Mass	Intensity
1	59.840	101.370	2	69.846	437.583	3	73.854	274.026	4	85.863	528.492	5	100.833	399.318
6	111.833	725.201	7	129.803	308.694	8	157.791	393.084	9	174.819	5189.708	10	184.781	253.916
11	202.795	276.433	12	228.818	253.908	13	242.789	524.626	14	246.775	150.569	15	270.846	513.006
16	287.869	905.603	17	299.828	378.465	18	313.856	325.729	19	317.793	404.945	20	357.802	541.391
21	431.844	1158.222	22	439.870	335.817	23	441.825	220.350	24	448.872	1159.970	25	456.913	1100.083
26	473.951	772.124	27	498.911	195.145	28	515.874	188.803	29	525.890	226.461	30	527.933	198.167
31	542.889	786.334	32	559.928	2498.327	33	565.996	266.541	34	584.994	254.202	35	602.051	864.090
36	616.989	209.650	37	656.068	265.087	38	679.103	348.157	39	684.045	303.659	40	699.106	637.395
41	701.044	458.358	42	716.156	8417.641	43	729.063	283.549	44	746.107	840.568	45	778.231	440.221
46	781.265	257.797	47	814.238	403.801	48	827.212	571.534	49	831.266	834.490	50	859.298	337.601
51	932.432	495.990	52	965.330	398.028	53	989.548	8714.517	54	999.542	363.953	55	1011.521	194.321
56	1014.629	322.874	57	1033.612	3667.912									

Calculated Masses:
TTDNQWLR

N-Term.	Ion	a	a-17	a-18	b	b-17	b-18	b+18	c	i	x	y	z	C-Term.	Ion
1	T	**74.060**	57.033	56.049	102.055	85.028	84.044	120.066	119.082	**74.060**	201.098	**175.119**	**158.092**	8	R
2	T	**175.108**	**158.081**	**157.097**	**203.103**	186.076	**185.092**	221.113	220.129	**74.060**	**314.182**	**288.203**	**271.176**	7	L
3	D	290.135	273.108	272.124	**318.130**	301.103	**300.119**	336.140	335.156	88.039	500.262	**474.282**	**457.256**	6	W
4	N	404.178	387.151	386.167	**432.173**	415.146	414.162	450.183	**449.199**	87.055	628.320	**602.341**	**585.314**	5	Q
5	Q	532.236	**515.210**	514.226	**560.231**	**543.205**	**542.221**	578.242	577.258	**101.071**	742.363	**716.384**	**699.357**	4	N
6	W	718.315	**701.289**	700.305	**746.310**	**729.284**	728.300	764.321	763.337	159.092	857.390	**831.411**	**814.384**	3	D
7	L	**831.400**	**814.373**	813.389	**859.394**	842.368	841.384	877.405	876.421	**86.096**	958.438	**932.458**	915.432	2	T
8	R	987.501	970.474	969.490	1015.496	998.469	997.485	**1033.506**	1032.522	**129.113**	1059.485	**1033.506**	1016.480	1	T

Copyright © 1999-2008 Bruker Daltonik GmbH

Area 8. Annotated MS/MS spectrum of ion 1708.910 *m*/*z*.

Spectrum Analysis Report

Sequence Name:		Parentmass:	1708.910	Mass Error:	0.066	MH+ (mono):	1708.845
MH+ (avg):	1709.882	Threshold (a.i.):	0.000	Tolerance (Da):	0.700	Number of Peaks:	89
Above Threshold:	89	Assigned Peaks:	32	Not assigned Peaks:	57		

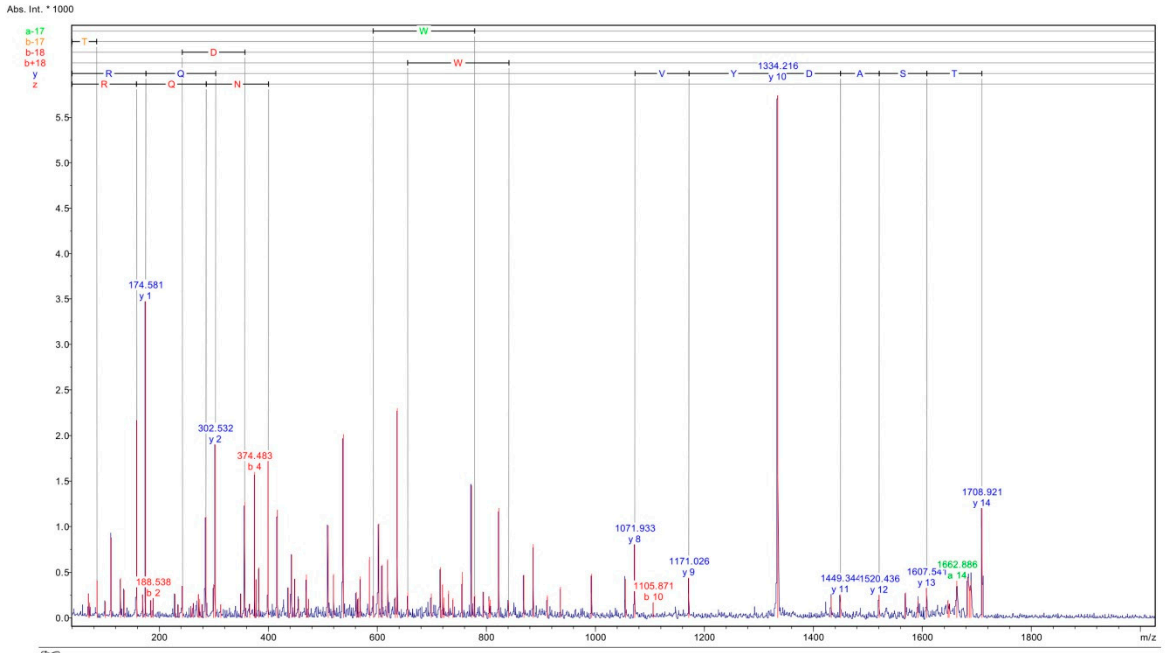

BioTools Version 3.2 Copyright © 1999-2008 Bruker Daltonik GmbH Page -1-

Spectrum Analysis Report

Display Parameter:

Parentmass:	1708.910	Mass Error:	0.066	MH+ (mono):	1708.845	MH+ (avg):	1709.882
Threshold (a.i.):	0.000	Tolerance (Da):	0.700	Number of Peaks:	89	Above Threshold:	89
Assigned Peaks:	32	Not assigned Peaks:	57				

Peaklist:

Peak	Mass	Intensity	Peak	Mass	Intensity	Peak	Mass	Intensity	Peak	Mass	Intensity	Peak	Mass	Intensity
1	69.693	264.716	2	71.760	168.365	3	85.680	405.676	4	100.650	181.989	5	111.640	882.285
6	128.632	439.026	7	135.584	308.408	8	158.585	2163.822	9	169.530	256.952	10	174.581	3475.044
11	184.535	193.875	12	188.538	215.046	13	228.543	262.439	14	234.530	143.860	15	242.509	351.518
16	257.549	131.234	17	262.534	163.152	18	271.557	263.750	19	273.472	207.729	20	278.489	150.691
21	285.521	1112.259	22	299.533	368.081	23	302.532	1901.775	24	312.520	149.129	25	349.474	266.467
26	356.512	1276.036	27	365.487	154.426	28	374.483	1600.544	29	377.472	426.950	30	382.493	555.788
31	399.512	1783.981	32	416.517	1188.761	33	436.448	333.033	34	442.485	693.721	35	448.497	428.419
36	455.529	209.125	37	469.541	475.858	38	473.507	212.889	39	509.489	1015.736	40	519.488	482.350
41	537.477	2014.807	42	561.500	286.127	43	564.494	218.821	44	568.521	454.901	45	585.518	668.474
46	592.509	245.185	47	602.549	1035.623	48	608.557	584.374	49	618.534	643.592	50	632.536	229.486
51	636.534	2300.353	52	655.594	281.546	53	698.553	267.778	54	715.620	556.423	55	719.556	369.977
56	721.553	224.098	57	730.609	299.273	58	738.594	215.727	59	755.607	506.658	60	772.647	1454.402
61	778.573	253.699	62	794.591	285.718	63	804.637	232.461	64	806.632	200.703	65	822.624	1204.604
66	840.688	189.552	67	868.720	470.238	68	885.748	811.804	69	911.696	247.492	70	935.725	342.734
71	992.750	485.733	72	1054.857	427.905	73	1071.933	806.260	74	1105.871	172.301	75	1171.026	442.651
76	1334.216	5888.905	77	1432.326	228.242	78	1449.344	256.885	79	1520.436	251.054	80	1569.133	279.825
81	1591.428	196.577	82	1607.541	328.454	83	1646.898	198.769	84	1648.764	154.734	85	1662.886	408.585
86	1682.764	449.988	87	1685.724	337.695	88	1688.633	405.012	89	1708.921	1202.921			

Calculated Masses:
TSADYVWIGLWNQR

N-Term.	Ion	a	a-17	a-18	b	b-17	b-18	b+18	c	i	x	y	z	C-Term.	Ion
1	T	74.060	57.033	56.049	102.055	**85.028**	84.044	120.066	119.082	74.060	201.098	**175.119**	**158.092**	14	R
2	S	161.092	144.066	143.082	**189.087**	172.060	171.076	207.098	206.114	60.044	329.157	**303.178**	**286.151**	13	Q
3	A	232.129	215.103	214.119	260.124	**243.098**	**242.114**	**278.135**	277.151	44.049	443.200	417.220	**400.194**	12	N
4	D	347.156	330.130	329.146	**375.151**	358.124	**357.140**	393.162	392.178	88.039	629.279	603.300	586.273	11	W
5	Y	510.219	493.193	492.209	538.214	521.188	520.204	556.225	555.241	**136.076**	742.363	716.384	699.357	10	L
6	V	609.288	**592.261**	591.277	637.283	620.256	619.272	**655.293**	654.309	**72.081**	799.385	773.405	756.379	9	G
7	W	795.367	**778.341**	777.357	823.362	**806.336**	805.352	**841.373**	**840.389**	**159.092**	912.469	886.489	869.463	8	I
8	I	908.451	891.425	890.441	936.446	919.420	918.436	954.457	953.473	**86.096**	1098.548	**1072.569**	**1055.542**	7	W
9	G	965.473	948.446	947.462	993.468	976.441	975.457	1011.478	1010.494	30.034	1197.616	**1171.637**	1154.611	6	V
10	L	1078.557	1061.530	1060.546	**1106.552**	1089.525	1088.541	1124.562	1123.578	**86.096**	1360.680	**1334.700**	1317.674	5	Y
11	W	1264.636	1247.610	1246.626	1292.631	1275.604	1274.620	1310.642	1309.658	**159.092**	1475.707	**1449.727**	**1432.701**	4	D
12	N	1378.679	1361.652	1360.668	1406.674	1389.647	1388.663	1424.684	1423.700	87.055	1546.744	**1520.764**	1503.738	3	A
13	Q	1506.738	1489.711	1488.727	1534.733	1517.706	1516.722	1552.743	1551.759	**101.071**	1633.776	**1607.797**	**1590.770**	2	S
14	R	**1662.839**	1645.812	1644.828	1690.834	1673.807	1672.823	**1708.844**	1707.860	**129.113**	1734.823	**1708.844**	1691.818	1	T

BioTools Version 3.2 Copyright © 1999-2008 Bruker Daltonik GmbH Page -2-

Area 8. Annotated MS/MS spectrum of ion 920.462 *m*/*z*.

Spectrum Analysis Report

Sequence Name:		Parentmass:	920.462	Mass Error:	0.050	MH+ (mono):	920.411
MH+ (avg):	920.945	Threshold (a.i.):	0.000	Tolerance (Da):	0.700	Number of Peaks:	35
Above Threshold:	35	Assigned Peaks:	22	Not assigned Peaks:	13		

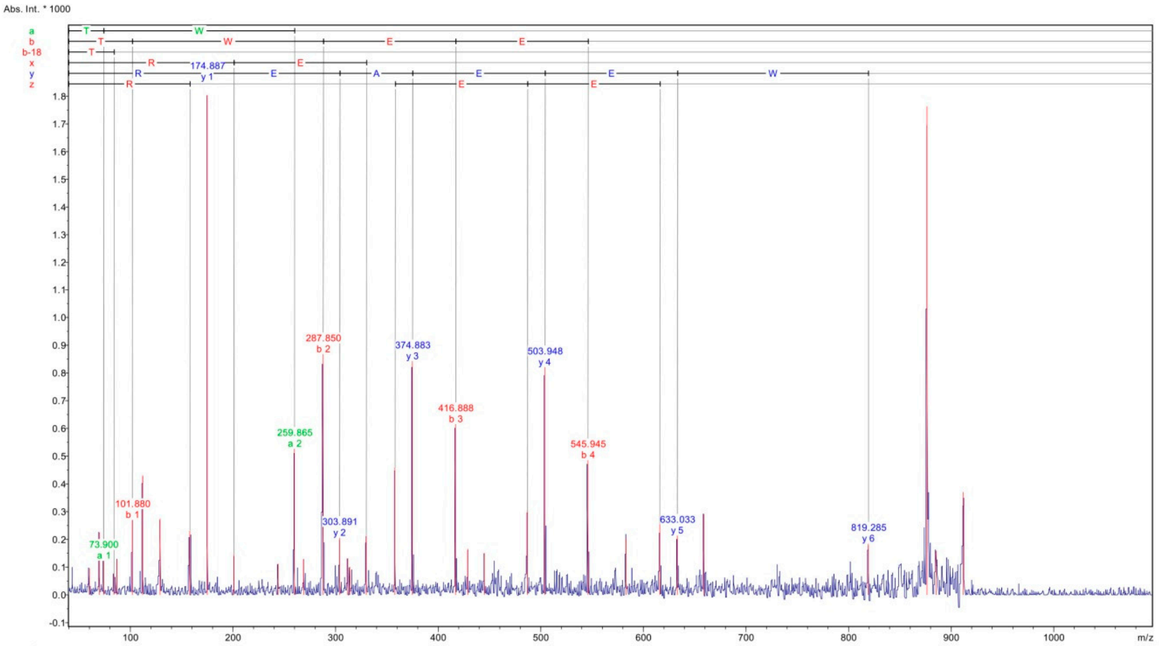

Spectrum Analysis Report

Display Parameter:

Parentmass:	920.462	Mass Error:	0.050	MH+ (mono):	920.411	MH+ (avg):	920.945
Threshold (a.i.):	0.000	Tolerance (Da):	0.700	Number of Peaks:	35	Above Threshold:	35
Assigned Peaks:	22	Not assigned Peaks:	13				

Peaklist:

Peak	Mass	Intensity	Peak	Mass	Intensity	Peak	Mass	Intensity	Peak	Mass	Intensity	Peak	Mass	Intensity
1	59.892	96.345	2	69.900	225.016	3	73.900	123.150	4	83.947	74.618	5	86.919	127.255
6	101.880	270.253	7	111.901	431.080	8	128.897	274.630	9	157.872	230.725	10	174.887	1851.706
11	200.840	143.767	12	243.862	112.382	13	259.865	526.660	14	268.850	129.909	15	287.850	867.312
16	303.891	205.426	17	311.832	131.390	18	313.858	100.588	19	329.831	210.146	20	357.859	458.432
21	374.883	842.048	22	416.888	615.411	23	428.870	165.409	24	444.870	150.927	25	486.878	301.315
26	503.948	820.205	27	545.945	485.607	28	583.036	205.010	29	615.987	254.610	30	633.033	213.817
31	659.039	291.379	32	819.285	184.862	33	876.392	1763.633	34	885.407	159.648	35	911.749	370.751

Calculated Masses:
TWEEAER

N-Term.	Ion	a	a-17	a-18	b	b-17	b-18	b+18	c	i	x	y	z	C-Term.	Ion
1	T	**74.060**	57.033	56.049	**102.055**	85.028	**84.044**	120.066	119.082	**74.060**	**201.098**	**175.119**	**158.092**	7	R
2	W	**260.139**	243.113	242.129	**288.134**	271.108	270.124	306.145	305.161	159.092	**330.141**	**304.162**	287.135	6	E
3	E	389.182	372.155	371.171	**417.177**	400.150	399.166	435.187	434.203	**102.055**	401.178	**375.199**	**358.172**	5	A
4	E	518.225	501.198	500.214	**546.219**	529.193	528.209	564.230	563.246	**102.055**	530.221	**504.241**	**487.215**	4	E
5	A	589.262	572.235	571.251	617.257	600.230	599.246	635.267	634.283	44.049	**659.263**	**633.284**	**616.257**	3	E
6	E	718.304	701.278	700.294	746.299	729.273	728.289	764.310	763.326	**102.055**	845.342	**819.363**	802.337	2	W
7	R	874.405	857.379	856.395	902.400	**885.374**	884.390	920.411	919.427	**129.113**	946.390	920.411	903.384	1	T

Area 8. Annotated MS/MS spectrum of ion 1427.645 *m*/*z*.

Spectrum Analysis Report

Sequence Name:		Parentmass:	1427.645	Mass Error:	0.018	MH+ (mono):	1427.627
MH+ (avg):	1428.550	Threshold (a.i.):	0.000	Tolerance (Da):	0.700	Number of Peaks:	55
Above Threshold:	55	Assigned Peaks:	27	Not assigned Peaks:	28		

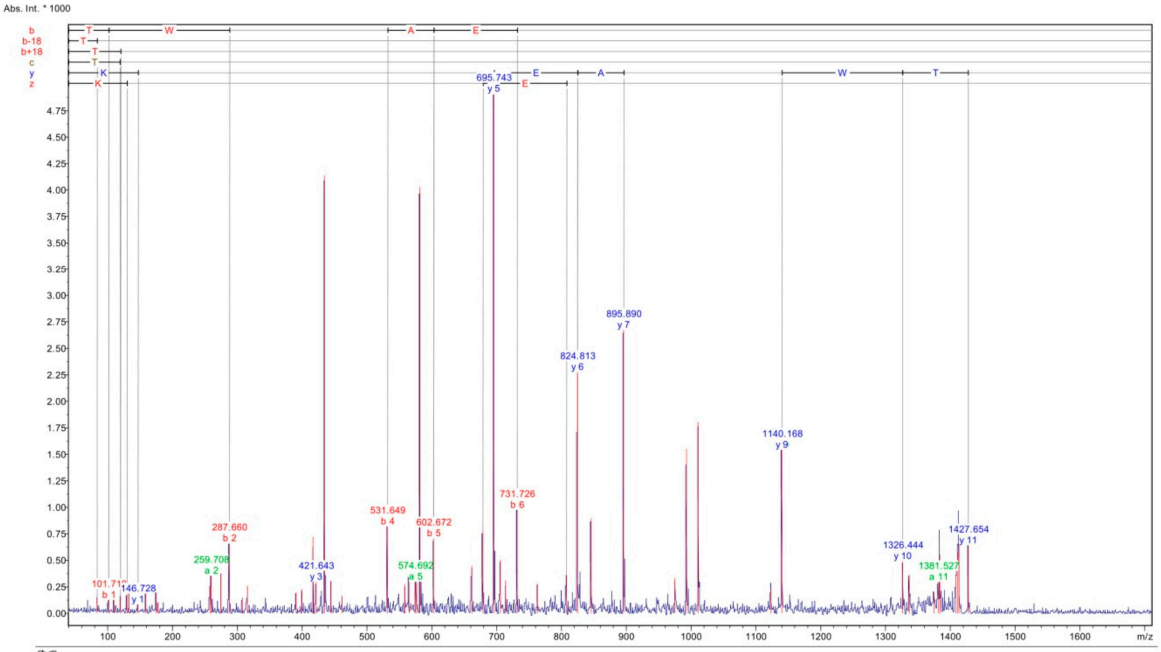

Spectrum Analysis Report

Display Parameter:

Parentmass:	1427.645	Mass Error:	0.018	MH+ (mono):	1427.627	MH+ (avg):	1428.550
Threshold (a.i.):	0.000	Tolerance (Da):	0.700	Number of Peaks:	55	Above Threshold:	55
Assigned Peaks:	27	Not assigned Peaks:	28				

Peaklist:

Peak	Mass	Intensity	Peak	Mass	Intensity	Peak	Mass	Intensity	Peak	Mass	Intensity	Peak	Mass	Intensity
1	83.770	144.580	2	101.718	125.030	3	109.708	136.340	4	119.712	151.598	5	129.709	170.773
6	132.661	242.407	7	146.728	83.109	8	158.690	236.530	9	174.694	198.569	10	177.668	103.013
11	257.694	256.620	12	259.708	354.628	13	274.702	378.302	14	287.660	659.658	15	307.634	136.978
16	315.641	258.347	17	390.651	196.284	18	399.664	231.286	19	416.652	721.904	20	421.643	297.300
21	434.672	4137.895	22	444.624	307.552	23	461.649	166.272	24	531.649	819.787	25	558.657	276.134
26	564.686	334.246	27	574.692	298.139	28	576.662	286.335	29	581.699	4029.075	30	602.672	707.719
31	661.697	447.624	32	678.712	789.238	33	695.743	4911.063	34	705.731	508.412	35	713.737	311.937
36	731.726	974.290	37	762.861	279.438	38	807.811	367.857	39	824.813	2277.068	40	845.795	896.486
41	895.890	2673.718	42	974.936	333.997	43	992.979	1552.715	44	1010.988	1807.045	45	1123.207	226.778
46	1140.168	1541.822	47	1326.444	491.708	48	1336.487	364.776	49	1374.892	199.717	50	1381.527	295.139
51	1383.731	553.662	52	1408.327	369.054	53	1411.200	573.370	54	1413.246	654.028	55	1427.654	641.563

Calculated Masses:
TWDEAEKFCNK 9: Carbamidomethyl (C)

N-Term.	Ion	a	a-17	a-18	b	b-17	b-18	b+18	c	i	x	y	z	C-Term.	Ion
1	T	74.060	57.033	56.049	**102.055**	85.028	**84.044**	**120.066**	**119.082**	74.060	173.092	**147.113**	**130.086**	11	K
2	W	**260.139**	243.113	242.129	**288.134**	271.108	270.124	306.145	305.161	**159.092**	**287.135**	261.156	244.129	10	N
3	D	375.166	358.140	357.156	403.161	386.135	385.151	**421.172**	420.188	88.039	447.166	**421.186**	404.160	9	C*
4	E	504.209	487.182	486.198	**532.204**	515.177	514.193	550.214	549.230	**102.055**	594.234	568.255	551.228	8	F
5	A	**575.246**	**558.219**	557.235	**603.241**	586.214	585.230	621.251	620.267	44.049	722.329	**696.350**	**679.323**	7	K
6	E	704.289	687.262	686.278	**732.284**	715.257	**714.273**	750.294	749.310	**102.055**	851.372	**825.392**	**808.366**	6	E
7	K	832.384	815.357	814.373	860.378	843.352	842.368	878.389	877.405	**101.107**	922.409	**896.429**	879.403	5	A
8	F	979.452	962.425	961.441	1007.447	990.420	989.436	1025.457	1024.473	**120.081**	1051.451	1025.472	1008.446	4	E
9	C*	**1139.483**	1122.456	1121.472	1167.478	1150.451	1149.467	1185.488	1184.504	**133.043**	1166.478	**1140.499**	**1123.472**	3	D
10	N	1253.526	1236.499	1235.515	1281.520	1264.494	1263.510	1299.531	1298.547	87.055	1352.558	**1326.578**	1309.552	2	W
11	K	**1381.621**	1364.594	1363.610	1409.615	1392.589	1391.605	**1427.626**	1426.642	**101.107**	1453.605	**1427.626**	**1410.599**	1	T

Area 8. Annotated MS/MS spectrum of ion 2161.931 *m*/*z*.

Spectrum Analysis Report

Sequence Name:
MH+ (avg): 2163.331
Above Threshold: 63
Parentmass: 2161.931
Threshold (a.i.): 0.000
Assigned Peaks: 16
Mass Error: 0.056
Tolerance (Da): 0.700
Not assigned Peaks: 47
MH+ (mono): 2161.875
Number of Peaks: 63

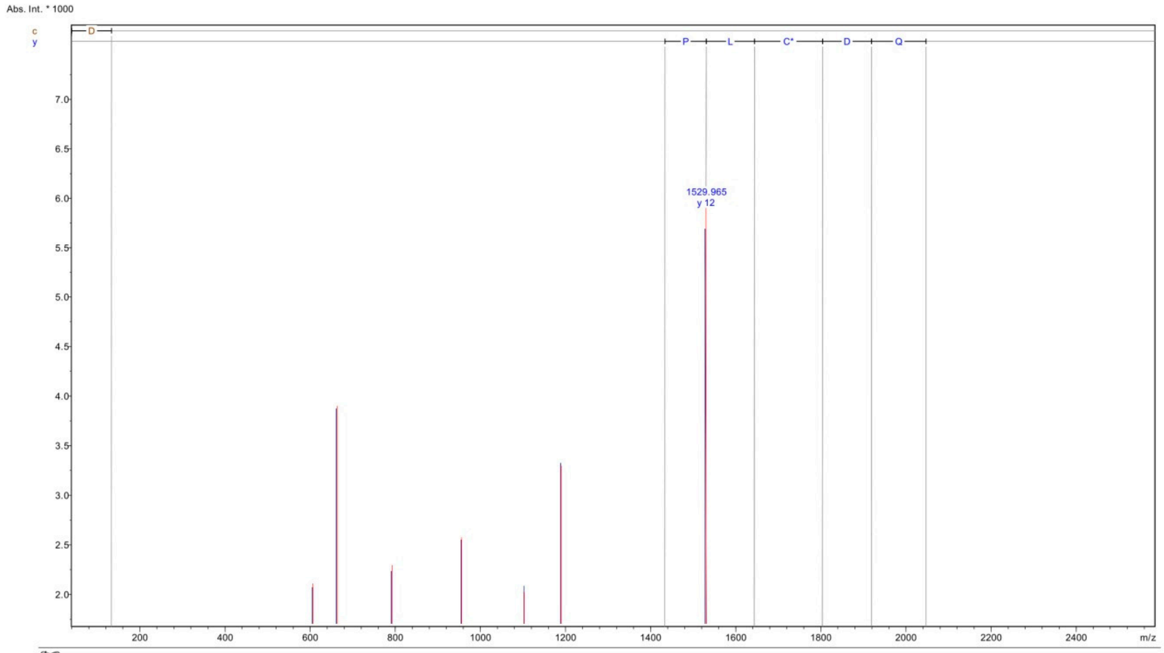

BioTools Version 3.2 Copyright © 1999-2008 Bruker Daltonik GmbH Page -1-

Spectrum Analysis Report

Display Parameter:

Parentmass: 2161.931
Threshold (a.i.): 0.000
Assigned Peaks: 16
Mass Error: 0.056
Tolerance (Da): 0.700
Not assigned Peaks: 47
MH+ (mono): 2161.875
Number of Peaks: 63
MH+ (avg): 2163.331
Above Threshold: 63

Peaklist:

Peak	Mass	Intensity	Peak	Mass	Intensity	Peak	Mass	Intensity	Peak	Mass	Intensity	Peak	Mass	Intensity
1	109.605	626.436	2	132.536	137.798	3	135.542	162.113	4	158.539	221.837	5	174.501	135.132
6	194.497	198.137	7	243.407	320.094	8	273.397	147.820	9	275.369	168.177	10	295.390	228.001
11	297.356	392.571	12	312.420	309.383	13	323.400	198.923	14	340.391	376.965	15	354.334	376.134
16	358.345	471.687	17	370.350	151.504	18	375.353	151.838	19	409.388	244.396	20	427.353	770.550
21	455.335	186.507	22	469.354	994.361	23	483.342	227.173	24	518.281	733.369	25	540.350	455.766
26	544.396	1103.196	27	556.375	255.489	28	574.355	268.653	29	606.357	2110.621	30	628.358	248.575
31	631.331	544.615	32	646.323	330.303	33	663.366	3900.165	34	692.399	276.235	35	709.410	258.500
36	720.385	221.843	37	737.390	315.773	38	774.366	328.726	39	792.382	2295.266	40	866.429	492.133
41	923.499	345.449	42	955.478	2576.076	43	971.449	293.864	44	1032.610	481.495	45	1060.534	652.296
46	1102.586	2022.853	47	1171.570	247.966	48	1189.634	3295.237	49	1220.682	250.039	50	1375.803	733.720
51	1432.915	646.540	52	1511.970	374.945	53	1529.965	5902.080	54	1643.089	534.352	55	1692.100	424.319
56	1803.202	776.980	57	1918.457	325.597	58	2028.536	261.291	59	2033.605	398.088	60	2046.688	623.408
61	2056.964	222.040	62	2070.386	445.106	63	2101.251	258.569						

Calculated Masses:
DQDCLPGWSFYEGHCYK 4: Carbamidomethyl (C) 15: Carbamidomethyl (C)

N-Term.	Ion	a	a-17	a-18	b	b-17	b-18	b+18	c	i	x	y	z	C-Term.	Ion
1	D	88.039	71.013	70.029	116.034	99.008	98.024	134.045	**133.061**	88.039	173.092	147.113	130.086	17	K
2	Q	216.098	199.071	198.087	**244.093**	227.066	226.082	262.103	261.119	101.071	336.155	310.176	293.150	16	Y
3	D	331.125	314.098	**313.114**	359.120	342.093	341.109	377.130	376.146	88.039	496.186	470.207	453.180	15	C*
4	C*	491.155	474.129	473.145	519.150	502.124	501.140	537.161	536.177	**133.043**	633.245	607.266	590.239	14	H
5	L	604.240	587.213	586.229	632.234	615.208	614.224	650.245	649.261	86.096	690.266	664.287	647.261	13	G
6	P	701.292	684.266	683.282	729.287	712.261	711.277	747.298	746.314	70.065	819.309	793.330	776.303	12	E
7	G	758.314	741.287	740.303	786.309	769.282	768.298	804.319	803.335	30.034	982.372	956.393	939.367	11	Y
8	W	944.393	927.367	926.383	972.388	**955.361**	954.377	990.399	989.415	**159.092**	1129.441	1103.461	1086.435	10	F
9	S	1031.425	1014.399	1013.415	1059.420	1042.393	1041.409	1077.431	1076.447	60.044	1216.473	1190.494	1173.467	9	S
10	F	1178.494	1161.467	1160.483	1206.488	**1189.462**	1188.478	1224.499	1223.515	120.081	1402.552	1376.573	1359.546	8	W
11	Y	1341.557	1324.530	1323.546	1369.552	1352.525	1351.541	1387.562	1386.578	**136.076**	1459.574	**1433.594**	1416.568	7	G
12	E	1470.599	1453.573	1452.589	1498.594	1481.568	1480.584	1516.605	1515.621	102.055	1556.626	**1530.647**	1513.621	6	P
13	G	1527.621	1510.594	1509.610	1555.616	1538.589	1537.605	1573.626	1572.642	30.034	1669.710	**1643.731**	1626.705	5	L
14	H	1664.680	1647.653	1646.669	**1692.675**	1675.648	1674.664	1710.685	1709.701	**110.071**	1829.741	**1803.762**	1786.735	4	C*
15	C*	1824.710	1807.684	1806.700	1852.705	1835.679	1834.695	1870.716	1869.732	**133.043**	1944.768	**1918.789**	1901.762	3	D
16	Y	1987.774	1970.747	1969.763	2015.769	1998.742	1997.758	**2033.779**	2032.795	**136.076**	2072.827	**2046.847**	2029.821	2	Q
17	K	2115.869	2098.842	2097.858	2143.864	2126.837	2125.853	2161.874	2160.890	101.107	2187.853	2161.874	2144.848	1	D

BioTools Version 3.2 Copyright © 1999-2008 Bruker Daltonik GmbH Page -2-

Area 8. Annotated MS/MS spectrum of ion 1936.046 *m*/*z*.

Spectrum Analysis Report

Sequence Name:		Parentmass:	1936.046	Mass Error:	0.017	MH+ (mono):	1936.029
MH+ (avg):	1937.184	Threshold (a.i.):	0.000	Tolerance (Da):	0.700	Number of Peaks:	79
Above Threshold:	79	Assigned Peaks:	24	Not assigned Peaks:	55		

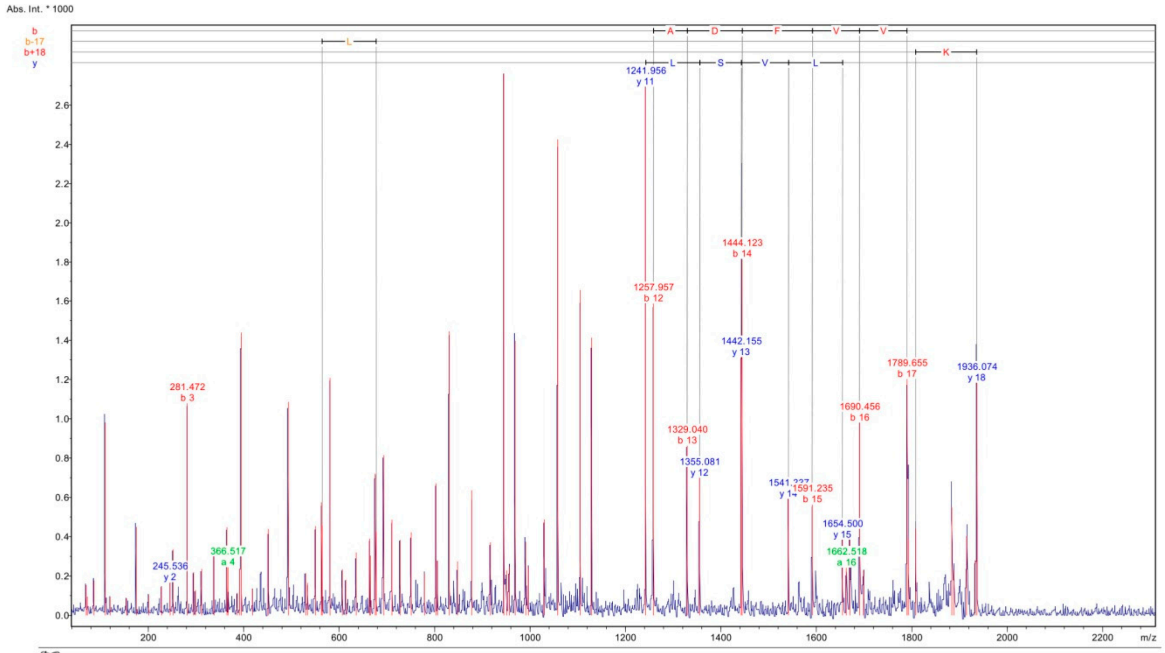

Spectrum Analysis Report

Display Parameter:

Parentmass:	1936.046	Mass Error:	0.017	MH+ (mono):	1936.029	MH+ (avg):	1937.184
Threshold (a.i.):	0.000	Tolerance (Da):	0.700	Number of Peaks:	79	Above Threshold:	79
Assigned Peaks:	24	Not assigned Peaks:	55				

Peaklist:

Peak	Mass	Intensity	Peak	Mass	Intensity	Peak	Mass	Intensity	Peak	Mass	Intensity	Peak	Mass	Intensity
1	69.677	154.820	2	71.696	95.671	3	85.696	178.250	4	109.621	981.962	5	119.626	94.650
6	154.564	87.186	7	174.557	450.011	8	200.504	104.328	9	227.550	148.959	10	245.536	167.856
11	251.501	336.381	12	281.472	1079.343	13	294.503	220.273	14	298.508	128.641	15	311.509	236.902
16	337.491	346.021	17	364.498	449.146	18	366.517	244.381	19	386.449	114.669	20	391.436	238.572
21	394.476	1439.806	22	418.461	134.276	23	451.474	439.732	24	493.500	1085.979	25	529.477	214.957
26	533.453	166.081	27	550.468	454.705	28	562.496	575.208	29	564.520	458.787	30	580.527	1209.039
31	606.470	235.204	32	613.460	181.354	33	635.528	319.868	34	663.529	390.557	35	665.576	303.680
36	675.578	721.667	37	677.590	428.300	38	693.568	816.350	39	710.521	487.196	40	727.544	384.830
41	750.530	423.310	42	778.512	212.332	43	802.634	671.223	44	805.857	278.654	45	830.626	1446.311
46	847.608	275.154	47	877.606	637.773	48	916.645	372.187	49	944.675	2861.350	50	950.678	228.988
51	956.815	248.936	52	968.699	1398.112	53	990.717	375.153	54	996.671	245.445	55	1029.821	488.316
56	1057.775	2426.812	57	1104.814	1655.506	58	1128.839	1413.678	59	1241.956	2694.034	60	1257.957	1586.885
61	1329.040	863.602	62	1355.081	699.442	63	1442.155	1313.748	64	1444.123	1816.136	65	1541.337	592.159
66	1591.235	564.368	67	1654.500	384.251	68	1662.518	242.214	69	1670.253	462.734	70	1690.456	979.883
71	1698.367	228.307	72	1789.655	1202.330	73	1792.824	389.558	74	1807.777	440.482	75	1883.748	549.109
76	1887.964	230.638	77	1914.725	403.868	78	1932.518	280.131	79	1936.074	1183.048			

Calculated Masses:
GSHLVSLHNIAEADFVVK

N-Term.	Ion	a	a-17	a-18	b	b-17	b-18	b+18	c	i	x	y	z	C-Term.	Ion
1	G	30.034	13.007	12.023	58.029	41.002	40.018	76.039	75.055	30.034	173.092	147.113	130.086	18	K
2	S	117.066	100.039	99.055	145.061	128.034	127.050	163.071	162.087	60.044	272.160	**246.181**	229.155	17	V
3	H	254.125	237.098	236.114	**282.120**	265.093	264.109	300.130	**299.146**	**110.071**	371.229	345.250	328.223	16	V
4	L	**367.209**	350.182	349.198	395.204	378.177	377.193	413.214	412.230	**86.096**	518.297	492.318	475.291	15	F
5	V	466.277	449.251	448.267	494.272	477.246	476.262	512.283	511.299	**72.081**	633.324	607.345	590.318	14	D
6	S	553.309	536.283	535.299	581.304	**564.278**	563.294	599.315	598.331	60.044	704.361	678.382	661.356	13	A
7	L	666.393	649.367	648.383	694.388	**677.362**	676.378	712.399	711.415	**86.096**	833.404	807.425	790.398	12	E
8	H	803.452	786.426	785.442	831.447	814.421	813.437	849.458	848.474	**110.071**	904.441	878.462	861.435	11	A
9	N	917.495	900.469	899.485	945.490	928.464	927.480	963.501	962.517	87.055	1017.525	991.546	974.519	10	I
10	I	1030.579	1013.553	1012.569	1058.574	1041.548	1040.564	1076.585	1075.601	**86.096**	1131.568	1105.589	1088.562	9	N
11	A	1101.616	1084.590	1083.606	1129.611	1112.585	1111.601	1147.622	1146.638	44.049	1268.627	**1242.648**	1225.621	8	H
12	E	1230.659	1213.632	1212.648	**1258.654**	**1241.627**	1240.643	1276.664	1275.680	102.055	1381.711	**1355.732**	1338.705	7	L
13	A	1301.696	1284.670	1283.685	**1329.691**	1312.664	1311.680	1347.702	1346.718	44.049	1468.743	**1442.764**	1425.737	6	S
14	D	1416.723	1399.696	1398.712	**1444.718**	1427.691	1426.707	1462.728	1461.744	88.039	1567.811	**1541.832**	1524.806	5	V
15	F	1563.791	1546.765	1545.781	**1591.786**	1574.760	1573.776	1609.797	1608.813	**120.081**	1680.896	**1654.916**	1637.890	4	L
16	V	**1662.860**	1645.833	1644.849	**1690.855**	1673.828	1672.844	1708.865	1707.881	**72.081**	1817.954	1791.975	1774.949	3	H
17	V	1761.928	1744.902	1743.918	**1789.923**	1772.897	1771.913	**1807.934**	1806.950	**72.081**	1904.986	1879.007	1861.981	2	S
18	K	1890.023	1872.997	1872.013	1918.018	1900.992	1900.008	**1936.029**	1935.045	101.107	1962.008	**1936.029**	1919.002	1	G

Area 8. Annotated MS/MS spectrum of ion 1041.686 *m*/*z*.

Spectrum Analysis Report

Sequence Name:		Parentmass:	1041.686	Mass Error:	0.065	MH+ (mono):	1041.621
MH+ (avg):	1042.257	Threshold (a.i.):	0.000	Tolerance (Da):	0.700	Number of Peaks:	76
Above Threshold:	76	Assigned Peaks:	34	Not assigned Peaks:	42		

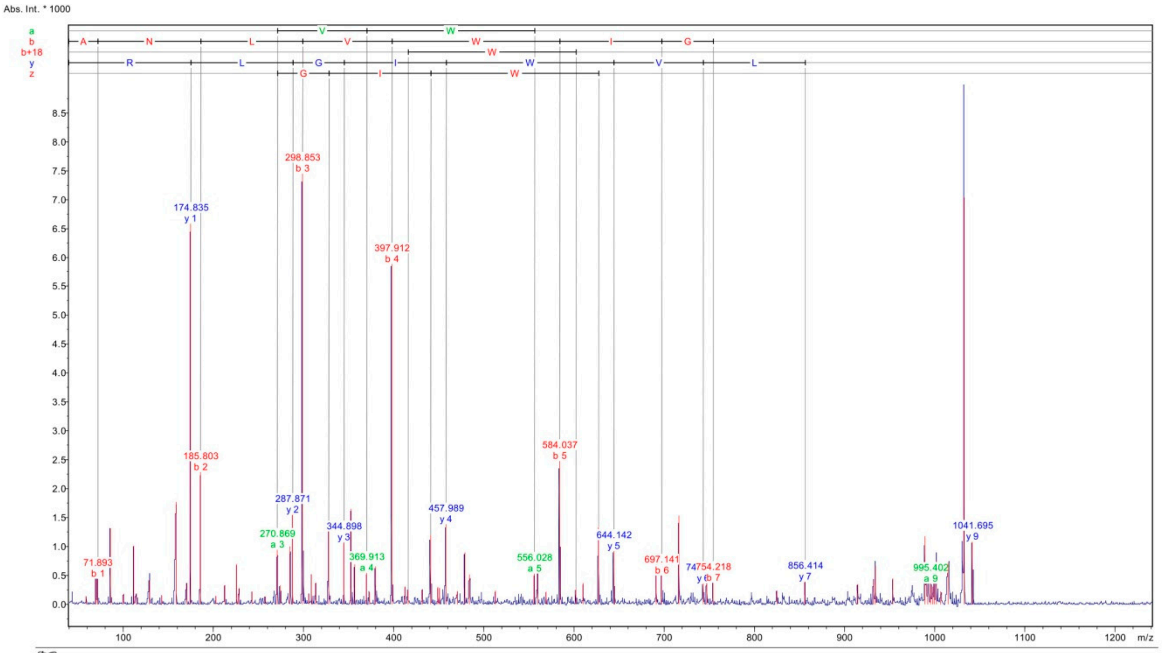

Spectrum Analysis Report

Display Parameter:

Parentmass:	1041.686	Mass Error:	0.065	MH+ (mono):	1041.621	MH+ (avg):	1042.257
Threshold (a.i.):	0.000	Tolerance (Da):	0.700	Number of Peaks:	76	Above Threshold:	76
Assigned Peaks:	34	Not assigned Peaks:	42				

Peaklist:

Peak	Mass	Intensity	Peak	Mass	Intensity	Peak	Mass	Intensity	Peak	Mass	Intensity	Peak	Mass	Intensity
1	58.851	137.124	2	69.861	772.296	3	71.893	440.719	4	85.881	1314.254	5	100.829	181.268
6	111.846	1013.434	7	114.838	187.135	8	128.850	420.626	9	142.840	151.688	10	158.818	1771.383
11	170.830	373.724	12	174.835	6585.556	13	185.803	2284.423	14	202.809	145.292	15	212.827	333.168
16	225.844	700.579	17	228.836	286.695	18	242.816	220.258	19	257.856	135.627	20	270.869	944.086
21	274.099	327.064	22	284.867	1001.860	23	287.871	1548.303	24	298.853	7453.691	25	306.078	181.800
26	308.778	522.573	27	313.841	374.249	28	327.869	1353.794	29	344.898	1072.731	30	352.820	1651.589
31	356.873	694.235	32	369.913	544.382	33	373.154	210.924	34	379.870	657.014	35	397.912	5886.700
36	412.882	310.080	37	415.974	243.411	38	431.939	262.680	39	440.956	1193.209	40	448.940	297.970
41	450.948	287.952	42	457.989	1385.009	43	470.898	229.754	44	478.936	896.981	45	483.993	517.410
46	512.975	239.338	47	556.028	532.459	48	560.066	632.899	49	568.959	222.656	50	584.037	2475.207
51	602.110	261.289	52	610.065	369.013	53	627.091	1335.037	54	644.142	921.254	55	691.127	776.181
56	697.141	499.976	57	716.264	1532.698	58	743.293	361.975	59	747.243	430.272	60	754.218	370.382
61	825.240	239.468	62	856.414	390.818	63	914.701	350.344	64	932.336	410.942	65	934.262	662.807
66	953.572	449.540	67	989.090	1177.005	68	992.282	417.785	69	995.402	355.985	70	997.639	306.464
71	999.677	505.286	72	1001.870	754.990	73	1006.633	223.915	74	1015.057	720.047	75	1032.799	7038.152
76	1041.695	1083.408												

Calculated Masses:
ANLVWIGLR

N-Term.	Ion	a	a-17	a-18	b	b-17	b-18	b+18	c	i	x	y	z	C-Term.	Ion
1	A	44.049	27.023	26.039	**72.044**	55.018	54.034	90.055	89.071	44.049	201.098	**175.119**	158.092	9	R
2	N	158.092	141.066	140.082	**186.087**	169.061	168.077	204.098	**203.114**	87.055	**314.182**	**288.203**	**271.176**	8	L
3	L	**271.176**	254.150	253.166	**299.171**	282.145	281.161	317.182	316.198	**86.096**	371.204	**345.224**	**328.198**	7	G
4	V	**370.245**	**353.218**	**352.234**	**398.240**	381.213	**380.229**	**416.250**	415.266	**72.081**	**484.288**	**458.309**	**441.282**	6	I
5	W	**556.324**	539.298	538.314	**584.319**	567.293	566.309	**602.330**	601.346	**159.092**	670.367	**644.388**	**627.361**	5	W
6	I	669.408	652.382	651.398	**697.403**	680.377	679.393	715.414	714.430	**86.096**	769.436	**743.456**	726.430	4	V
7	G	726.430	709.403	708.419	**754.425**	737.398	736.414	772.435	771.451	30.034	882.520	**856.540**	839.514	3	L
8	L	839.514	822.487	821.503	867.509	850.482	849.498	885.519	884.535	**86.096**	996.563	970.583	**953.557**	2	N
9	R	**995.615**	978.588	977.604	1023.610	**1006.583**	1005.599	**1041.620**	1040.636	**129.113**	1067.600	**1041.620**	1024.594	1	A

Area 9. Annotated MS/MS spectrum of ion 1533.641*m*/*z*.

Spectrum Analysis Report

Sequence Name:		Parentmass:	1533.641	Mass Error:	0.091	MH+ (mono):	1533.550
MH+ (avg):	1534.750	Threshold (a.i.):	0.000	Tolerance (Da):	0.700	Number of Peaks:	70
Above Threshold:	70	Assigned Peaks:	40	Not assigned Peaks:	30		

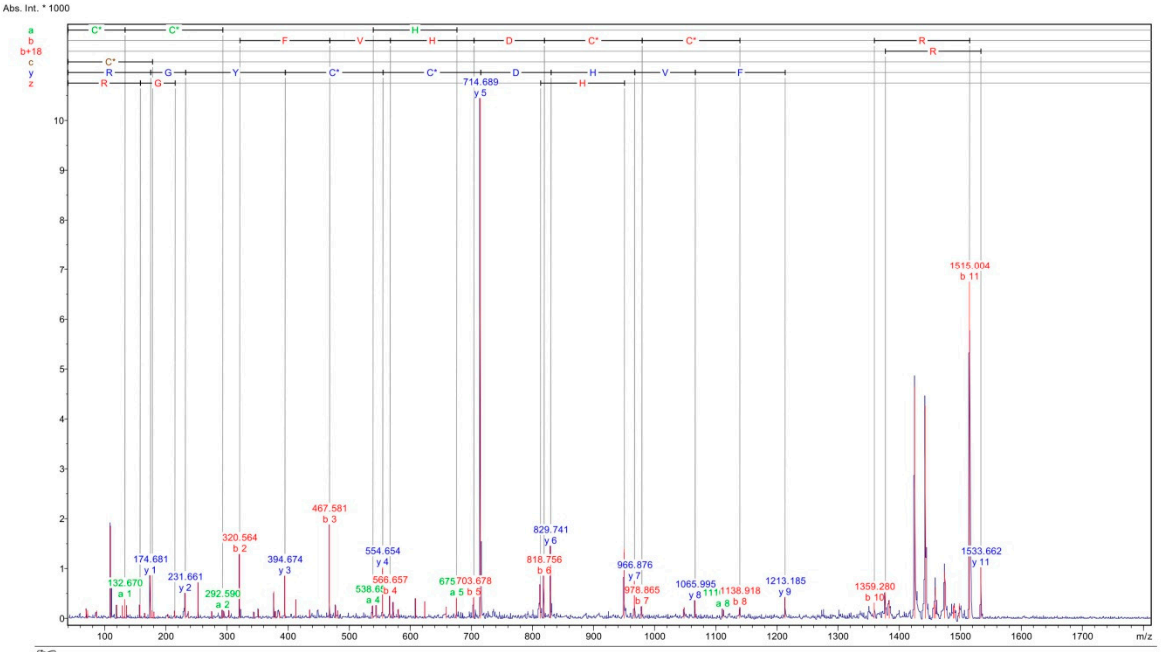

Copyright © 1999-2008 Bruker Daltonik GmbH

Spectrum Analysis Report

Display Parameter:

Parentmass:	1533.641	Mass Error:	0.091	MH+ (mono):	1533.550	MH+ (avg):	1534.750
Threshold (a.i.):	0.000	Tolerance (Da):	0.700	Number of Peaks:	70	Above Threshold:	70
Assigned Peaks:	40	Not assigned Peaks:	30				

Peaklist:

Peak	Mass	Intensity	Peak	Mass	Intensity	Peak	Mass	Intensity	Peak	Mass	Intensity	Peak	Mass	Intensity
1	69.759	184.457	2	71.774	164.499	3	86.754	159.733	4	109.711	1858.705	5	119.744	276.796
6	128.736	252.970	7	132.670	388.055	8	135.700	245.413	9	157.693	274.324	10	165.643	109.525
11	174.681	864.403	12	177.634	157.492	13	180.709	122.772	14	203.612	80.306	15	214.661	153.981
16	231.661	513.149	17	236.643	146.664	18	252.626	719.782	19	275.587	141.123	20	292.590	172.229
21	295.631	142.503	22	303.579	158.836	23	320.564	1295.980	24	344.613	126.182	25	351.674	186.977
26	376.598	546.027	27	379.650	147.150	28	386.017	149.093	29	394.674	858.297	30	412.596	382.739
31	435.544	182.326	32	467.581	1883.469	33	477.993	259.981	34	481.574	145.291	35	538.656	262.590
36	544.640	442.969	37	554.654	1029.054	38	566.657	450.838	39	572.603	327.638	40	580.528	191.364
41	608.649	407.815	42	623.664	343.345	43	658.690	229.026	44	675.716	420.223	45	703.678	429.796
46	714.689	10450.668	47	812.732	682.540	48	818.756	855.050	49	829.741	1455.148	50	949.835	1402.541
51	966.876	750.879	52	978.865	245.201	53	1047.907	229.734	54	1065.995	364.157	55	1110.971	195.414
56	1138.918	233.732	57	1213.185	430.765	58	1359.280	317.139	59	1377.288	526.091	60	1383.776	375.366
61	1425.305	4653.307	62	1442.458	4263.258	63	1457.065	280.733	64	1459.323	625.393	65	1474.514	952.622
66	1488.745	262.311	67	1490.638	257.867	68	1498.523	300.063	69	1515.004	6752.401	70	1533.662	1023.673

Calculated Masses:

CCFVHDCCYGR 1: Carbamidomethyl (C) 2: Carbamidomethyl (C) 7: Carbamidomethyl (C) 8: Carbamidomethyl (C)

N-Term.	Ion	a	a-17	a-18	b	b-17	b-18	b+18	c	i	x	y	z	C-Term.	Ion
1	C*	**133.043**	116.016	115.032	161.038	144.011	143.027	179.048	**178.064**	**133.043**	201.098	**175.119**	**158.092**	11	R
2	C*	**293.074**	**276.047**	**275.063**	**321.069**	**304.042**	**303.058**	339.079	338.095	**133.043**	258.120	**232.140**	**215.114**	10	G
3	F	440.142	423.116	422.132	**468.137**	451.110	450.126	486.148	485.164	**120.081**	421.183	**395.204**	378.177	9	Y
4	V	**539.210**	522.184	521.200	**567.205**	550.179	549.195	585.216	584.232	**72.081**	**581.214**	**555.234**	**538.208**	8	C*
5	H	**676.269**	**659.243**	**658.259**	**704.264**	687.238	686.254	722.275	721.291	**110.071**	741.244	**715.265**	698.238	7	C*
6	D	791.296	774.270	773.286	**819.291**	802.265	801.281	837.302	836.318	88.039	856.271	**830.292**	**813.265**	6	D
7	C*	951.327	934.300	933.316	**979.322**	962.295	961.311	997.332	996.348	**133.043**	993.330	**967.351**	**950.324**	5	H
8	C*	**1111.358**	1094.331	1093.347	**1139.353**	1122.326	1121.342	1157.363	1156.379	**133.043**	1092.399	**1066.419**	1049.393	4	V
9	Y	1274.421	1257.394	1256.410	1302.416	1285.389	1284.405	1320.426	1319.442	**136.076**	1239.467	**1213.488**	1196.461	3	F
10	G	1331.442	1314.416	1313.432	**1359.437**	1342.411	1341.427	**1377.448**	1376.464	30.034	1399.498	1373.518	1356.492	2	C*
11	R	1487.544	1470.517	1469.533	**1515.538**	**1498.512**	1497.528	**1533.549**	1532.565	**129.113**	1559.528	**1533.549**	1516.522	1	C*

Copyright © 1999-2008 Bruker Daltonik GmbH

Area 9. Annotated MS spectrum.

Spectrum Analysis Report

Sequence Name: Acidic phospholipase A2 ammodytin I1 OS=Vipera ammodytes ammodytes PE=2 SV=1 PA2A1_VIPAA
Parentmass:
Mass Error: MH+ (mono): 1.008 MH+ (avg): 1.008 Threshold (a.i.): 0.000
Tolerance (Da): 0.500 Number of Peaks: 13 Above Threshold: Assigned Peaks:
Not assigned Peaks:

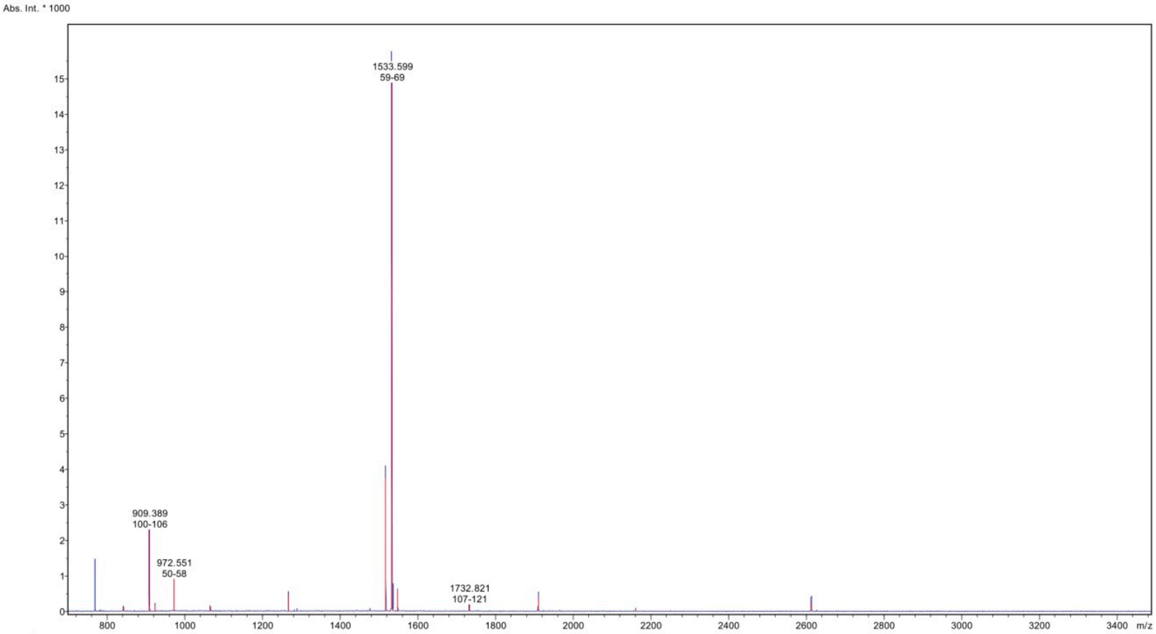

Spectrum Analysis Report

Sequence data:

Acidic phospholipase A2 ammodytin I1 OS=Vipera ammodytes ammodytes PE=2 SV=1 PA2A1_VIPAA

Intensity Coverage: 75.4 % (18321 cnts) Sequence Coverage MS: 30.4%
Sequence Coverage MS/MS: 0.0% pI (isoelectric point): 4.9

10	20	30	40	50	60	70	80	90	100	110	120	130	140
MRILWIVAVC	LIGAEGHLSQ	FGDMINKKTG	IFGIMSYIYY	GCYCGWGGKG	KPLDATDRCC	FVHDCCYGRV	NGCDPKMGTY	SYSFQNGDIV	CGGDDPCLRA	VCECDRVAAI	CFGENMNTYD	KKYMLYSLFD	CKEESEQC

Display Parameter:

Sequence Name: Acidic phospholipase A2 ammodytin I1 OS=Vipera ammodytes ammodytes PE=2 SV=1 PA2A1_VIPAA MH+ (mono): 1.008
MH+ (avg): 1.008 Threshold (a.i.): 0.000 Tolerance (Da): 0.500 Number of Peaks: 13

Peaklist:

Peak	Mass	Intensity	Peak	Mass	Intensity	Peak	Mass	Intensity	Peak	Mass	Intensity	Peak	Mass	Intensity
1	842.539	140.884	2	909.389	2308.851	3	923.402	173.497	4	972.551	916.339	5	1066.457	139.605
6	1266.668	496.586	7	1516.560	3745.875	8	1533.599	14892.758	9	1547.616	583.525	10	1732.821	202.740
11	1909.862	393.958	12	2159.941	63.187	13	2613.122	255.339						

Area 10. Annotated MS spectrum.

Spectrum Analysis Report

Sequence Name:	Neutral phospholipase A2 ammodytin I2 OS=Vipera ammodytes ammodytes PE=1 SV=2 PA2N2_VIPAA					Parentmass:	
Mass Error:		MH+ (mono):	1.008	MH+ (avg):	1.008	Threshold (a.i.):	0.000
Tolerance (Da):	0.500	Number of Peaks:	15	Above Threshold:		Assigned Peaks:	
Not assigned Peaks:							

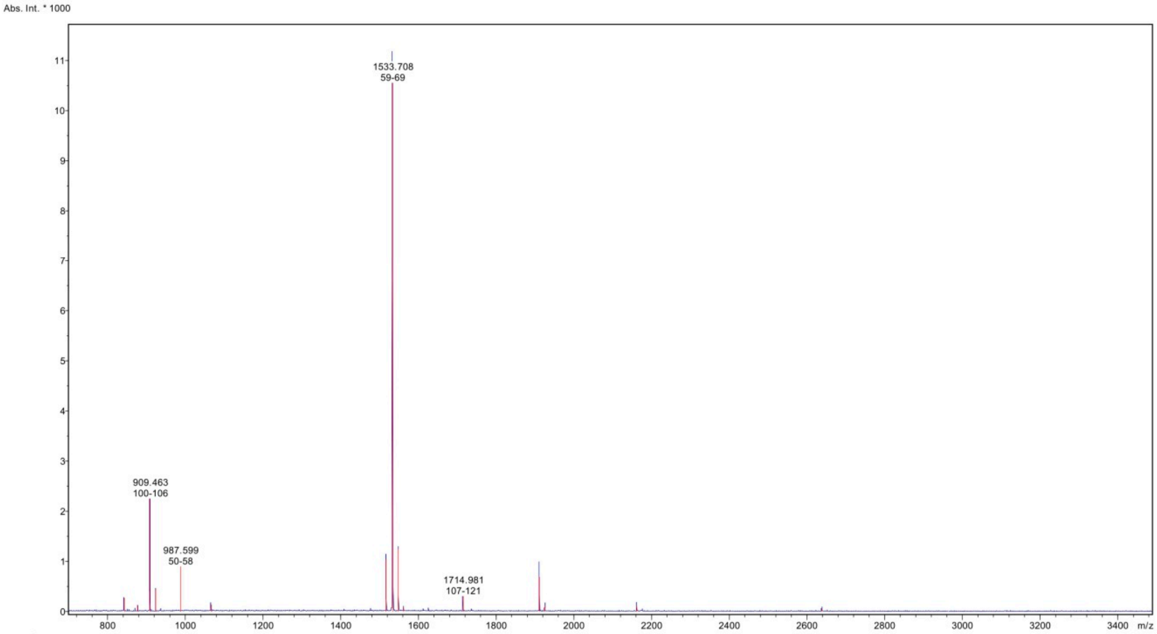

Spectrum Analysis Report

Sequence data:

Neutral phospholipase A2 ammodytin I2 OS=Vipera ammodytes ammodytes PE=1 SV=2 PA2N2_VIPAA

Intensity Coverage:	76.5 % (14018 cnts)	Sequence Coverage MS:	30.7%
Sequence Coverage MS/MS:	0.0%	pI (isoelectric point):	6.1

10	20	30	40	50	60	70	80	90	100	110	120	130	140
MRTLWIVAVC	LIGVEGNLYQ	FGNMIFKMTK	KSALLSYSNY	GCYCGWGGKG	KPQDATDRCC	FVHDCCYGRV	NGCDPKLSIY	SYSFENGDIV	CGGDDPCLRA	VCECDRVAAI	CFGENLNTYD	KKYKNYPSSH	CTETEQC

Display Parameter:

Sequence Name:	Neutral phospholipase A2 ammodytin I2 OS=Vipera ammodytes ammodytes PE=1 SV=2 PA2N2_VIPAA					MH+ (mono):	1.008
MH+ (avg):	1.008	Threshold (a.i.):	0.000	Tolerance (Da):	0.500	Number of Peaks:	15

Peaklist:

Peak	Mass	Intensity	Peak	Mass	Intensity	Peak	Mass	Intensity	Peak	Mass	Intensity	Peak	Mass	Intensity
1	842.605	275.797	2	877.480	118.239	3	909.463	2253.037	4	923.479	438.802	5	987.599	901.986
6	1066.548	137.022	7	1516.673	1039.958	8	1533.708	10554.174	9	1547.726	1249.471	10	1561.741	84.402
11	1714.981	309.068	12	1910.983	693.719	13	1924.992	111.181	14	2161.090	108.325	15	2637.441	54.449

Area 11. Annotated MS/MS spectrum of ion 989.439 *m*/*z*.

Spectrum Analysis Report

Sequence Name:		Parentmass:	989.439	Mass Error:	0.046	MH+ (mono):	989.393
MH+ (avg):	990.100	Threshold (a.i.):	0.000	Tolerance (Da):	0.700	Number of Peaks:	40
Above Threshold:	40	Assigned Peaks:	24	Not assigned Peaks:	16		

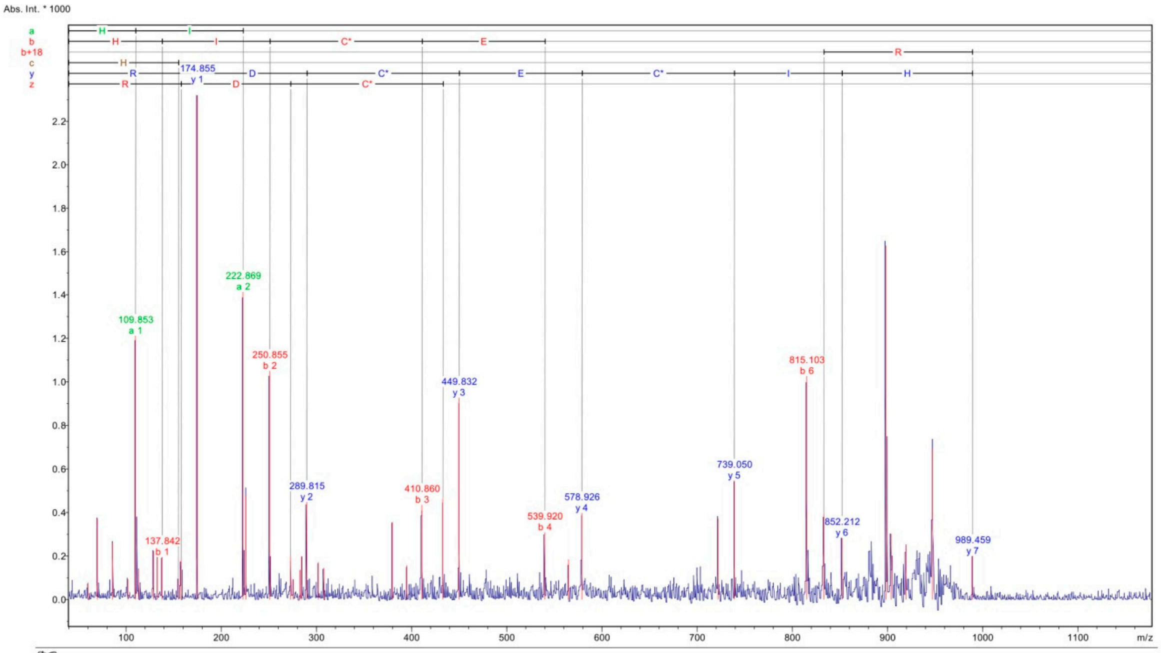

BioTools Version 3.2 Copyright © 1999-2008 Bruker Daltonik GmbH Page -1-

Spectrum Analysis Report

Display Parameter:

Parentmass:	989.439	Mass Error:	0.046	MH+ (mono):	989.393	MH+ (avg):	990.100
Threshold (a.i.):	0.000	Tolerance (Da):	0.700	Number of Peaks:	40	Above Threshold:	40
Assigned Peaks:	24	Not assigned Peaks:	16				

Peaklist:

Peak	Mass	Intensity	Peak	Mass	Intensity	Peak	Mass	Intensity	Peak	Mass	Intensity	Peak	Mass	Intensity
1	59.899	77.496	2	69.869	370.617	3	85.903	260.132	4	101.839	94.743	5	109.853	1211.232
6	112.854	141.094	7	128.879	223.549	8	132.813	244.789	9	137.842	195.090	10	154.805	93.903
11	157.852	185.799	12	174.855	2369.797	13	222.869	1413.132	14	225.896	477.811	15	250.855	1050.164
16	272.809	198.924	17	275.785	93.526	18	282.863	137.328	19	284.859	198.474	20	289.815	448.011
21	301.866	171.788	22	307.820	145.634	23	379.859	356.316	24	394.897	157.520	25	410.860	434.687
26	432.836	464.084	27	449.832	926.776	28	539.920	308.317	29	564.891	184.573	30	578.926	397.446
31	722.010	371.777	32	739.050	546.438	33	815.103	1026.358	34	833.154	390.114	35	852.212	283.284
36	898.294	1628.348	37	903.826	302.778	38	919.449	247.021	39	947.486	692.258	40	989.459	199.990

Calculated Masses:
HICECDR 3: Carbamidomethyl (C) 5: Carbamidomethyl (C)

N-Term.	Ion	a	a-17	a-18	b	b-17	b-18	b+18	c	i	x	y	z	C-Term.	Ion
1	H	**110.071**	93.045	92.061	**138.066**	121.040	120.056	156.077	**155.093**	**110.071**	201.098	**175.119**	**158.092**	7	R
2	I	**223.155**	206.129	205.145	**251.150**	234.124	233.140	269.161	268.177	**86.096**	316.125	**290.146**	**273.119**	6	D
3	C*	383.186	366.159	365.175	**411.181**	394.154	393.170	429.191	428.207	**133.043**	476.156	**450.177**	**433.150**	5	C*
4	E	512.229	495.202	494.218	**540.223**	523.197	522.213	558.234	557.250	**102.055**	605.198	**579.219**	562.193	4	E
5	C*	672.259	655.233	654.249	700.254	683.228	682.244	718.265	717.281	**133.043**	765.229	**739.250**	**722.223**	3	C*
6	D	787.286	770.260	769.276	**815.281**	798.255	797.271	**833.292**	832.308	88.039	878.313	**852.334**	835.307	2	I
7	R	943.387	926.361	925.377	971.382	954.356	953.372	**989.393**	988.409	**129.113**	1015.372	**989.393**	972.366	1	H

BioTools Version 3.2 Copyright © 1999-2008 Bruker Daltonik GmbH Page -2-

Area 11. Annotated MS/MS spectrum of ion 2315.846 *m*/*z*.

Spectrum Analysis Report

Sequence Name:		Parentmass:	2315.846	Mass Error:	-0.030	MH+ (mono):	2315.876
MH+ (avg):	2317.614	Threshold (a.i.):	0.000	Tolerance (Da):	0.700	Number of Peaks:	133
Above Threshold:	133	Assigned Peaks:	76	Not assigned Peaks:	57		

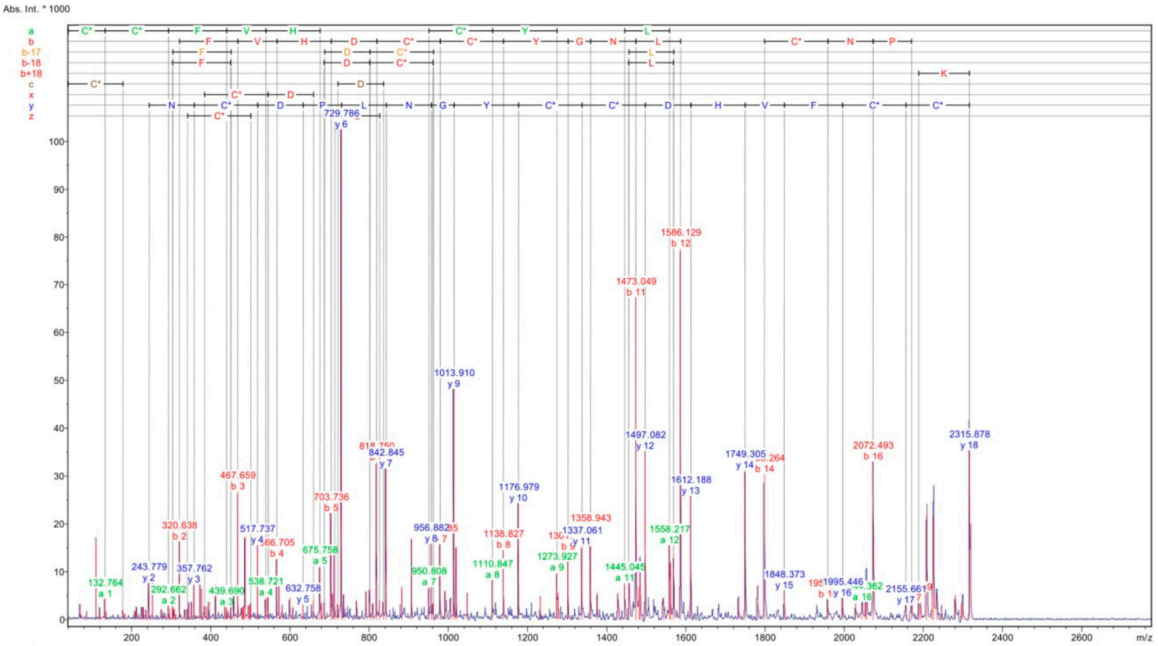

Spectrum Analysis Report

Display Parameter:

Parentmass:	2315.846	Mass Error:	-0.030	MH+ (mono):	2315.876	MH+ (avg):	2317.614
Threshold (a.i.):	0.000	Tolerance (Da):	0.700	Number of Peaks:	133	Above Threshold:	133
Assigned Peaks:	76	Not assigned Peaks:	57				

Peaklist:

Peak	Mass	Intensity	Peak	Mass	Intensity	Peak	Mass	Intensity	Peak	Mass	Intensity	Peak	Mass	Intensity
1	69.835	3323.631	2	85.870	1806.194	3	109.791	16575.486	4	119.790	2527.654	5	132.764	4310.402
6	135.772	1531.165	7	177.717	2136.735	8	180.755	1349.242	9	208.767	1743.057	10	212.729	2532.317
11	225.771	2599.753	12	229.692	2651.214	13	236.757	1976.276	14	243.779	7669.571	15	252.705	9784.153
16	275.677	2200.537	17	284.704	1429.853	18	292.662	2925.245	19	295.719	2277.048	20	303.664	2721.956
21	305.687	2485.889	22	307.719	2051.593	23	320.638	16327.553	24	334.686	2049.199	25	340.741	2178.592
26	344.667	3513.083	27	351.713	3751.988	28	357.762	7354.471	29	372.666	7241.597	30	376.673	6497.578
31	384.681	2814.755	32	389.693	2817.929	33	395.684	3775.439	34	412.659	7254.088	35	435.635	4238.147
36	439.690	2624.163	37	441.675	2039.566	38	450.659	2645.488	39	458.691	5515.320	40	467.659	26772.775
41	478.313	2377.915	42	481.668	2746.102	43	483.697	2773.955	44	486.674	17511.269	45	494.674	3035.849
46	498.752	3224.113	47	500.711	3374.961	48	517.737	15591.602	49	538.721	4632.675	50	544.671	5015.718
51	566.705	12727.221	52	572.677	7916.564	53	580.832	3042.718	54	599.720	4597.290	55	607.737	2630.831
56	632.758	3170.547	57	658.731	4238.861	58	675.758	11199.948	59	679.126	3297.711	60	686.722	4428.455
61	703.736	22258.667	62	707.713	5546.365	63	712.778	15376.515	64	720.745	3554.634	65	729.786	102545.693
66	735.715	5339.472	67	767.751	3978.271	68	792.729	5991.596	69	801.745	6671.822	70	809.752	3418.658
71	818.750	32878.624	72	825.835	4008.935	73	835.760	3713.484	74	842.845	31538.196	75	882.780	6710.969
76	906.764	16889.329	77	950.808	6770.747	78	956.882	15839.828	79	961.805	3518.784	80	978.785	15737.410
81	991.835	6044.433	82	1005.835	4447.149	83	1013.910	48188.290	84	1019.834	15224.634	85	1047.833	5593.151
86	1110.847	8285.072	87	1138.827	14578.848	88	1176.979	24345.546	89	1231.924	4850.080	90	1273.927	9828.960
91	1276.963	5753.704	92	1301.901	14169.934	93	1337.061	15288.406	94	1358.943	17856.434	95	1376.013	5769.962
96	1428.114	5691.570	97	1445.045	7632.407	98	1455.905	12410.109	99	1473.049	67258.749	100	1481.595	6657.965
101	1484.494	11923.532	102	1497.082	35225.640	103	1543.083	4711.431	104	1558.217	15544.975	105	1561.003	11921.577
106	1568.871	13729.939	107	1586.129	77628.380	108	1612.188	25838.653	109	1732.369	4685.087	110	1749.305	31212.226
111	1781.430	7314.006	112	1798.264	30407.899	113	1848.373	6077.801	114	1958.300	4263.803	115	1995.446	4555.725
116	2028.531	3186.483	117	2044.362	3650.401	118	2054.149	6851.685	119	2056.209	6083.308	120	2072.493	33017.458
121	2155.661	3035.221	122	2169.739	3483.556	123	2187.855	6503.294	124	2191.141	4148.637	125	2207.687	21964.765
126	2221.438	5035.118	127	2224.885	21891.081	128	2233.420	5577.542	129	2245.793	2219.869	130	2280.059	5232.673
131	2296.197	3067.901	132	2298.909	3735.444	133	2315.878	35364.975						

Calculated Masses:

CCFVHDCCYGNLPDCNPK 1: Carbamidomethyl (C) 2: Carbamidomethyl (C) 7: Carbamidomethyl (C) 8: Carbamidomethyl (C) 15: Carbamidomethyl (C)

N-Term.	Ion	a	a-17	a-18	b	b-17	b-18	b+18	c	i	x	y	z	C-Term.	Ion
1	C*	**133.043**	116.016	115.032	161.038	144.011	143.027	179.048	**178.064**	**133.043**	173.092	147.113	130.086	18	K
2	C*	**293.074**	**276.047**	**275.063**	**321.069**	**304.042**	**303.058**	339.079	338.095	**133.043**	270.145	**244.166**	227.139	17	P
3	F	**440.142**	423.116	422.132	**468.137**	**451.110**	**450.126**	**486.148**	485.164	**120.081**	**384.188**	**358.208**	**341.182**	16	N
4	V	**539.210**	522.184	521.200	**567.205**	550.179	549.195	585.216	584.232	72.081	**544.218**	**518.239**	**501.213**	15	C*
5	H	**676.269**	**659.243**	**658.259**	**704.264**	**687.238**	**686.254**	722.275	**721.291**	**110.071**	**659.245**	**633.266**	616.240	14	D
6	D	791.296	774.270	773.286	**819.291**	**802.265**	**801.281**	837.302	**836.318**	88.039	756.298	**730.319**	**713.292**	13	P
7	C*	**951.327**	934.300	933.316	**979.322**	**962.295**	**961.311**	997.332	996.348	**133.043**	869.382	**843.403**	**826.376**	12	L
8	C*	**1111.358**	1094.331	1093.347	**1139.353**	1122.326	1121.342	1157.363	1156.379	**133.043**	983.425	**957.446**	940.419	11	N
9	Y	**1274.421**	1257.394	1256.410	**1302.416**	1285.389	1284.405	1320.426	1319.442	**136.076**	1040.447	**1014.467**	997.441	10	G
10	G	1331.442	1314.416	1313.432	**1359.437**	1342.411	1341.427	1377.448	**1376.464**	30.034	1203.510	**1177.531**	1160.504	9	Y
11	N	**1445.485**	**1428.459**	**1427.475**	**1473.480**	**1456.454**	**1455.470**	1491.491	1490.507	87.055	1363.541	**1337.561**	1320.535	8	C*
12	L	**1558.569**	1541.543	1540.559	**1586.564**	**1569.538**	**1568.554**	1604.575	1603.591	**86.096**	1523.571	**1497.592**	1480.565	7	C*
13	P	1655.622	1638.596	1637.612	1683.617	1666.591	1665.607	1701.628	1700.644	**70.065**	1638.598	**1612.619**	1595.592	6	D
14	D	1770.649	1753.623	1752.639	**1798.644**	**1781.617**	1780.633	1816.655	1815.671	88.039	1775.657	**1749.678**	**1732.651**	5	H
15	C*	1930.680	1913.653	1912.669	**1958.675**	1941.648	1940.664	1976.685	1975.701	**133.043**	1874.725	**1848.746**	1831.720	4	V
16	N	**2044.723**	2027.696	2026.712	**2072.718**	**2055.691**	**2054.707**	2090.728	2089.744	87.055	2021.794	**1995.815**	1978.788	3	F
17	P	2141.775	2124.749	2123.765	**2169.770**	2152.744	2151.760	**2187.781**	2186.797	**70.065**	2181.825	**2155.845**	2138.819	2	C*
18	K	2269.870	2252.844	2251.860	2297.865	2280.839	**2279.855**	**2315.876**	2314.892	101.107	2341.855	**2315.876**	**2298.849**	1	C*

Area 11. Annotated MS/MS spectrum of ion 1105.502 *m*/*z*.

Spectrum Analysis Report

Sequence Name:		Parentmass:	1105.502	Mass Error:	-0.000	MH+ (mono):	1105.503
MH+ (avg):	1106.274	Threshold (a.i.):	0.000	Tolerance (Da):	0.700	Number of Peaks:	33
Above Threshold:	33	Assigned Peaks:	14	Not assigned Peaks:	19		

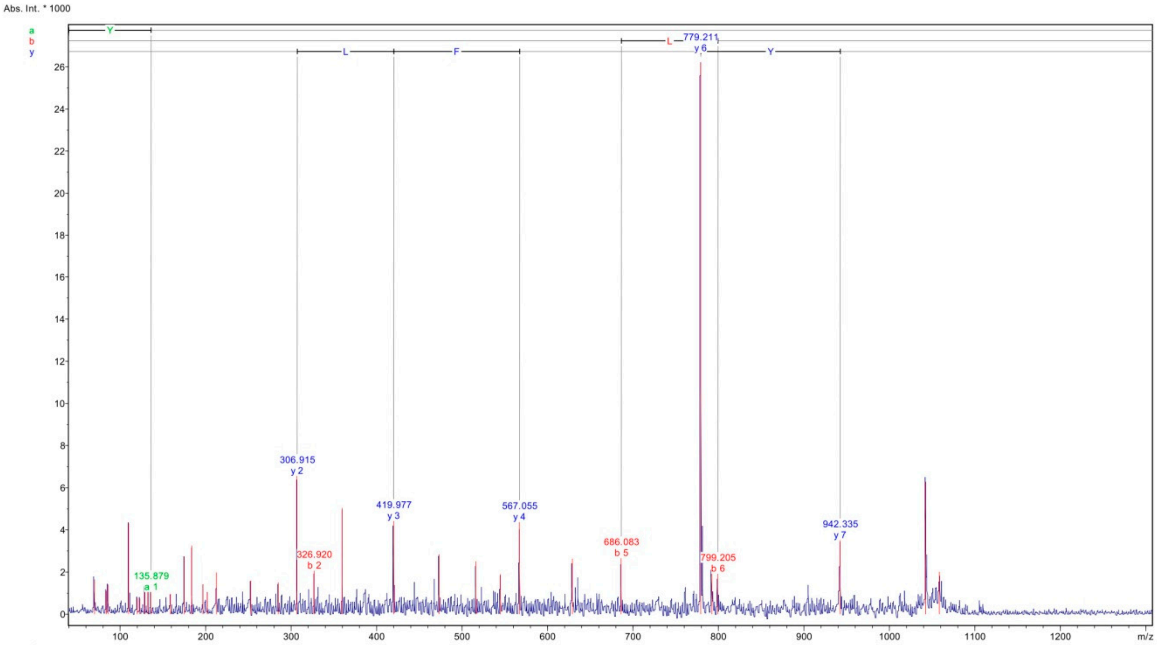

 Copyright © 1999-2008 Bruker Daltonik GmbH

Spectrum Analysis Report

Display Parameter:

Parentmass:	1105.502	Mass Error:	-0.000	MH+ (mono):	1105.503	MH+ (avg):	1106.274
Threshold (a.i.):	0.000	Tolerance (Da):	0.700	Number of Peaks:	33	Above Threshold:	33
Assigned Peaks:	14	Not assigned Peaks:	19				

Peaklist:

Peak	Mass	Intensity	Peak	Mass	Intensity	Peak	Mass	Intensity	Peak	Mass	Intensity	Peak	Mass	Intensity
1	69.919	1652.840	2	83.910	1114.795	3	85.932	1406.645	4	109.880	4332.888	5	119.884	779.261
6	122.919	745.787	7	128.922	1318.721	8	132.873	1182.715	9	135.879	1052.849	10	158.889	967.887
11	174.916	2729.286	12	183.875	3255.213	13	196.916	1427.052	14	201.900	1054.820	15	212.877	1986.217
16	252.877	1604.988	17	284.948	1512.695	18	306.915	6554.001	19	326.920	2064.297	20	359.920	5037.013
21	419.977	4422.943	22	472.994	2847.974	23	516.006	2514.686	24	544.925	1904.235	25	567.055	4371.380
26	629.067	2639.973	27	686.083	2662.096	28	779.211	26648.386	29	792.151	2127.911	30	799.205	1922.823
31	942.335	3508.690	32	1042.608	6290.131	33	1058.448	2020.588						

Calculated Masses:
YYPDFLCK 7: Carbamidomethyl (C)

N-Term.	Ion	a	a-17	a-18	b	b-17	b-18	b+18	c	i	x	y	z	C-Term.	Ion
1	Y	**136.076**	119.049	118.065	164.071	147.044	146.060	182.081	181.097	**136.076**	173.092	147.113	130.086	8	K
2	Y	299.139	282.112	281.128	**327.134**	310.107	309.123	345.144	344.160	**136.076**	333.123	**307.143**	290.117	7	C*
3	P	396.192	379.165	378.181	424.187	407.160	406.176	442.197	441.213	**70.065**	446.207	**420.228**	403.201	6	L
4	D	511.219	494.192	493.208	539.214	522.187	521.203	557.224	556.240	88.039	593.275	**567.296**	550.269	5	F
5	F	658.287	641.261	640.277	**686.282**	669.256	668.271	704.293	703.309	**120.081**	708.302	682.323	665.296	4	D
6	L	771.371	754.345	753.361	**799.366**	782.340	781.356	817.377	816.393	**86.096**	805.355	**779.376**	762.349	3	P
7	C*	931.402	914.375	913.391	959.397	**942.370**	941.386	977.407	976.423	**133.043**	968.418	**942.439**	925.412	2	Y
8	K	1059.497	**1042.470**	1041.486	1087.492	1070.465	1069.481	1105.502	1104.518	101.107	1131.482	1105.502	1088.476	1	Y

 Copyright © 1999-2008 Bruker Daltonik GmbH

Area 11. Annotated MS spectrum 1.

Spectrum Analysis Report

Sequence Name:	Basic phospholipase A2 vurtoxin OS=Vipera renardi PE=1 SV=1 PA2B_VIPRE					Parentmass:	
Mass Error:		MH+ (mono):	1.008	MH+ (avg):	1.008	Threshold (a.i.):	0.000
Tolerance (Da):	0.500	Number of Peaks:	5	Above Threshold:		Assigned Peaks:	
Not assigned Peaks:							

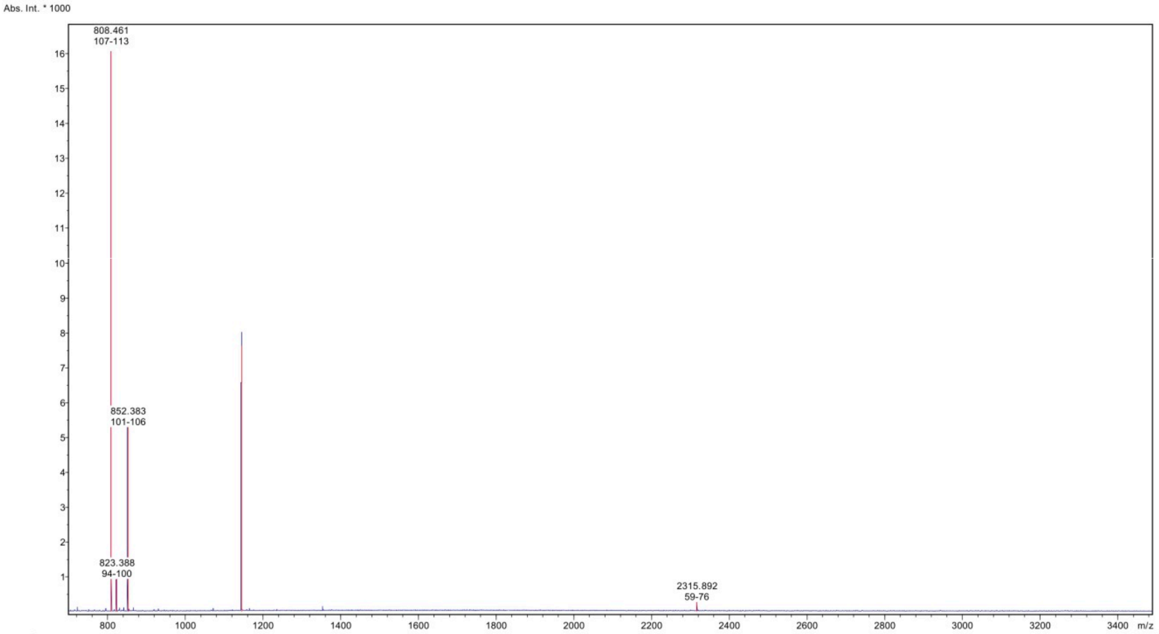

 Copyright © 1999-2008 Bruker Daltonik GmbH

Spectrum Analysis Report

Sequence data:

Basic phospholipase A2 vurtoxin OS=Vipera renardi PE=1 SV=1 PA2B_VIPRE

Intensity Coverage:	74.9 % (22721 cnts)	Sequence Coverage MS:	27.5%
Sequence Coverage MS/MS:	0.0%	pI (isoelectric point):	9.7

10	20	30	40	50	60	70	80	90	100	110	120	130	140
MRTLWIVAVC	LIGVEGSLLE	FGMMILEETG	KNPLTSYSFY	GCYCGVGGKG	TPKDATDRCC	FVHDCCYGNL	PDCNPKIDRY	KYHRKNGAIV	CGKGTSCENR	ICECDRAAAI	CFRKNLKTYN	HIYKYYPDFL	CKKESEKC

Display Parameter:

Sequence Name:	Basic phospholipase A2 vurtoxin OS=Vipera renardi PE=1 SV=1 PA2B_VIPRE					MH+ (mono):	1.008
MH+ (avg):	1.008	Threshold (a.i.):	0.000	Tolerance (Da):	0.500	Number of Peaks:	5

Peaklist:

Peak	Mass	Intensity	Peak	Mass	Intensity	Peak	Mass	Intensity	Peak	Mass	Intensity	Peak	Mass	Intensity
1	808.461	16204.710	2	823.388	942.130	3	852.383	5297.561	4	1144.657	7632.483	5	2315.892	276.539

 Copyright © 1999-2008 Bruker Daltonik GmbH

Annotated MS/MS spectrum of peptide 1072.570 *m*/*z*.

Spectrum Analysis Report

Sequence Name:		Parentmass:	1072.570	Mass Error:	-0.045	MH+ (mono):	1072.616
MH+ (avg):	1073.268	Threshold (a.i.):	0.000	Tolerance (Da):	1.000	Number of Peaks:	72
Above Threshold:	72	Assigned Peaks:	25	Not assigned Peaks:	47		

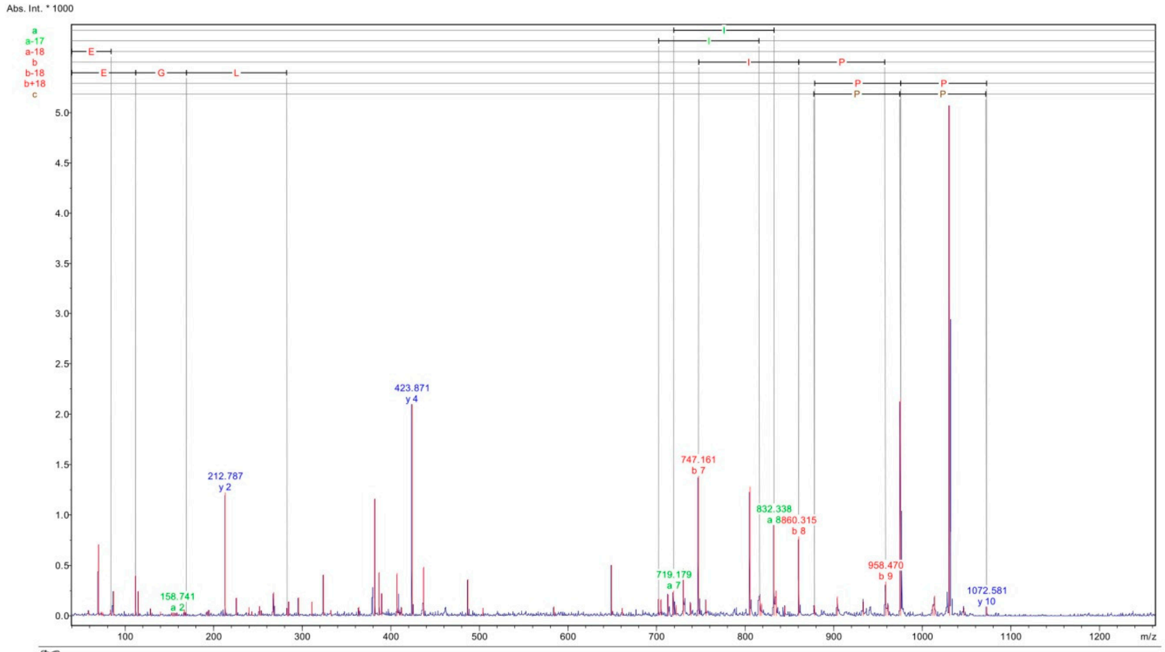

BioTools Version 3.2 Copyright © 1999-2008 Bruker Daltonik GmbH Page -1-

Spectrum Analysis Report

Display Parameter:

Parentmass:	1072.570	Mass Error:	-0.045	MH+ (mono):	1072.616	MH+ (avg):	1073.268
Threshold (a.i.):	0.000	Tolerance (Da):	1.000	Number of Peaks:	72	Above Threshold:	72
Assigned Peaks:	25	Not assigned Peaks:	47				

Peaklist:

Peak	Mass	Intensity	Peak	Mass	Intensity	Peak	Mass	Intensity	Peak	Mass	Intensity	Peak	Mass	Intensity
1	58.804	54.030	2	69.839	706.327	3	72.860	36.175	4	74.464	36.691	5	83.811	57.510
6	86.841	244.965	7	111.814	400.346	8	114.805	244.389	9	128.812	70.708	10	139.787	38.978
11	152.753	31.544	12	154.738	39.733	13	156.788	27.874	14	158.741	30.524	15	166.817	63.399
16	168.807	36.753	17	192.772	41.137	18	194.798	57.016	19	212.787	1224.182	20	225.804	177.360
21	239.782	84.221	22	242.971	41.094	23	251.784	96.501	24	253.788	50.710	25	267.788	235.639
26	282.844	77.055	27	284.803	140.101	28	295.811	182.333	29	310.831	141.388	30	323.800	407.748
31	332.356	57.669	32	363.875	90.283	33	381.859	1160.679	34	386.577	433.045	35	389.830	220.190
36	406.855	426.061	37	409.834	61.784	38	412.115	81.432	39	423.871	2098.973	40	436.890	486.695
41	486.911	363.486	42	503.899	79.299	43	584.014	92.371	44	649.080	504.030	45	661.140	78.668
46	702.173	174.050	47	705.057	166.669	48	713.174	211.742	49	719.179	249.012	50	721.982	107.020
51	730.159	466.316	52	738.556	129.187	53	747.161	1389.334	54	755.542	161.062	55	805.274	1281.351
56	816.301	192.431	57	818.061	125.222	58	832.338	899.077	59	834.916	240.836	60	844.849	99.838
61	860.315	788.382	62	878.328	101.207	63	903.880	192.609	64	933.449	141.219	65	958.470	340.228
66	961.521	108.111	67	975.477	2152.227	68	1011.579	115.466	69	1013.666	201.053	70	1030.623	5318.954
71	1046.841	77.855	72	1072.581	90.239									

Calculated Masses:
EGLPPRPIPP

N-Term.	Ion	a	a-17	a-18	b	b-17	b-18	b+18	c	i	x	y	z	C-Term.	Ion
1	E	102.055	85.028	**84.044**	130.050	113.023	**112.039**	148.060	147.076	102.055	142.050	116.071	99.044	10	P
2	G	**159.076**	142.050	141.066	187.071	170.045	**169.061**	205.082	204.098	30.034	**239.103**	**213.123**	196.097	9	P
3	L	272.160	255.134	**254.150**	300.155	**283.129**	**282.145**	318.166	317.182	**86.096**	352.187	326.207	309.181	8	I
4	P	369.213	352.187	351.203	397.208	380.182	379.198	415.219	414.235	**70.065**	449.239	**423.260**	**406.234**	7	P
5	P	466.266	449.239	448.255	494.261	477.234	476.250	512.271	511.287	**70.065**	605.341	579.361	562.335	6	R
6	R	622.367	605.341	604.357	650.362	633.335	632.351	668.373	667.389	**129.113**	**702.393**	676.414	659.388	5	P
7	P	**719.420**	**702.393**	**701.409**	**747.415**	**730.388**	**729.404**	765.425	764.441	**70.065**	799.446	773.467	**756.440**	4	P
8	I	**832.504**	**815.477**	814.493	**860.499**	843.472	842.488	**878.509**	**877.525**	**86.096**	912.530	886.551	869.524	3	L
9	P	929.557	912.530	911.546	**957.552**	940.525	939.541	**975.562**	**974.578**	**70.065**	969.552	943.572	926.546	2	G
10	P	1026.609	1009.583	1008.599	1054.604	1037.578	1036.594	**1072.615**	**1071.631**	**70.065**	1098.594	**1072.615**	1055.588	1	E

BioTools Version 3.2 Copyright © 1999-2008 Bruker Daltonik GmbH Page -2-

Annotated MS/MS spectrum of peptide 1144.620 *m*/*z*.

Spectrum Analysis Report

Sequence Name:		Parentmass:	1144.620	Mass Error:	0.008	MH+ (mono):	1144.611
MH+ (avg):	1145.291	Threshold (a.i.):	0.000	Tolerance (Da):	0.700	Number of Peaks:	80
Above Threshold:	80	Assigned Peaks:	27	Not assigned Peaks:	53		

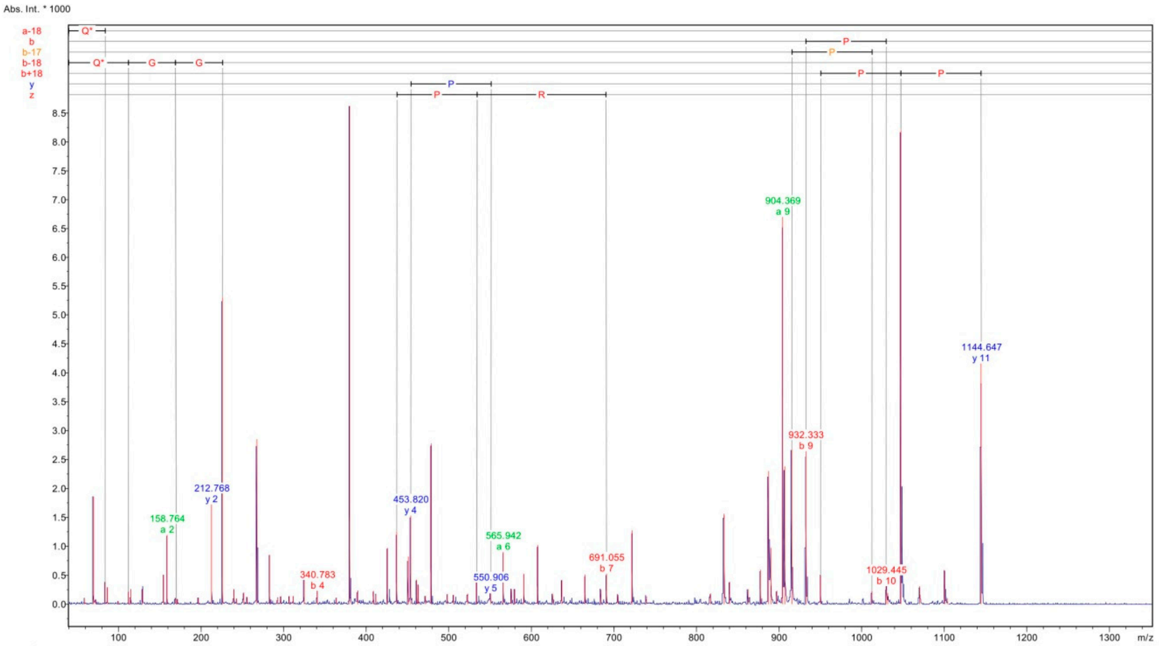

Spectrum Analysis Report

Display Parameter:

Parentmass:	1144.620	Mass Error:	0.008	MH+ (mono):	1144.611	MH+ (avg):	1145.291
Threshold (a.i.):	0.000	Tolerance (Da):	0.700	Number of Peaks:	80	Above Threshold:	80
Assigned Peaks:	27	Not assigned Peaks:	53				

Peaklist:

Peak	Mass	Intensity	Peak	Mass	Intensity	Peak	Mass	Intensity	Peak	Mass	Intensity	Peak	Mass	Intensity
1	58.791	103.476	2	69.819	1860.238	3	72.822	80.027	4	83.814	381.228	5	86.826	291.511
6	99.817	51.066	7	111.799	209.413	8	114.788	260.626	9	128.796	258.306	10	154.759	506.468
11	158.764	1197.544	12	169.747	105.671	13	171.767	88.021	14	196.780	113.379	15	212.768	1724.577
16	225.796	5302.895	17	239.767	263.537	18	243.028	100.978	19	251.772	203.776	20	255.796	119.337
21	267.766	2850.166	22	282.798	855.307	23	292.907	109.182	24	296.844	138.984	25	306.823	137.075
26	311.801	140.826	27	324.824	431.749	28	340.783	228.515	29	363.855	114.328	30	379.843	8676.980
31	389.694	222.945	32	408.803	229.689	33	411.823	164.105	34	425.847	973.333	35	436.812	1254.270
36	450.939	821.932	37	453.820	1531.076	38	460.908	426.047	39	463.024	324.146	40	471.818	144.241
41	478.916	2779.339	42	498.421	175.671	43	505.906	158.235	44	522.949	182.777	45	533.891	444.683
46	550.906	185.526	47	565.942	903.974	48	576.008	263.444	49	579.968	260.246	50	590.905	526.665
51	607.946	1022.627	52	625.970	183.899	53	637.058	417.223	54	665.045	511.490	55	684.305	252.554
56	691.055	526.389	57	705.090	169.210	58	722.107	1279.763	59	739.311	138.043	60	817.205	187.382
61	833.234	1565.577	62	840.072	387.379	63	862.298	261.169	64	877.262	596.878	65	887.318	2302.499
66	889.968	975.334	67	897.289	234.537	68	904.369	6705.468	69	906.895	2382.668	70	915.296	2672.872
71	932.333	2644.201	72	950.359	496.575	73	1012.407	220.651	74	1029.445	309.359	75	1031.628	195.551
76	1047.507	8209.386	77	1070.114	309.290	78	1100.624	578.195	79	1103.675	103.328	80	1144.647	4160.337

Calculated Masses:

QGGPPRPQIPP 1: Deamidated (NQ)

N-Term.	Ion	a	a-17	a-18	b	b-17	b-18	b+18	c	i	x	y	z	C-Term.	Ion
1	Q*	102.055	85.028	**84.044**	130.050	113.023	**112.039**	148.060	147.076	102.055	142.050	116.071	99.044	11	P
2	G	**159.076**	142.050	141.066	187.071	**170.045**	**169.061**	205.082	204.098	30.034	**239.103**	**213.123**	**196.097**	10	P
3	G	216.098	199.071	198.087	244.093	227.066	**226.082**	262.103	261.119	30.034	352.187	326.207	309.181	9	I
4	P	313.151	296.124	295.140	**341.146**	324.119	323.135	359.156	358.172	**70.065**	480.245	**454.266**	**437.239**	8	Q
5	P	410.203	393.177	392.193	438.198	421.172	420.188	456.209	455.225	**70.065**	577.298	**551.319**	**534.292**	7	P
6	R	**566.305**	549.278	548.294	594.299	577.273	**576.289**	612.310	611.326	**129.113**	733.399	707.420	**690.393**	6	R
7	P	663.357	646.331	645.347	**691.352**	674.326	673.342	709.363	708.379	**70.065**	830.452	804.473	787.446	5	P
8	Q	791.416	774.389	773.405	819.411	802.384	801.400	837.421	836.437	101.071	927.505	901.525	884.499	4	P
9	I	**904.500**	**887.473**	886.489	**932.495**	**915.468**	914.484	**950.505**	949.521	86.096	984.526	958.547	941.520	3	G
10	P	1001.553	984.526	983.542	**1029.548**	**1012.521**	1011.537	**1047.558**	1046.574	**70.065**	1041.548	1015.568	998.542	2	G
11	P	1098.605	1081.579	1080.595	1126.600	1109.574	1108.590	**1144.611**	1143.627	**70.065**	1170.590	**1144.611**	1127.584	1	Q*

Annotated MS/MS spectrum of peptide 1166.597 *m*/*z*.

Spectrum Analysis Report

Sequence Name:
MH+ (avg): 1167.296
Above Threshold: 141
Parentmass: 1166.597
Threshold (a.i.): 0.000
Assigned Peaks: 30
Mass Error: 0.001
Tolerance (Da): 1.000
Not assigned Peaks: 111
MH+ (mono): 1166.596
Number of Peaks: 141

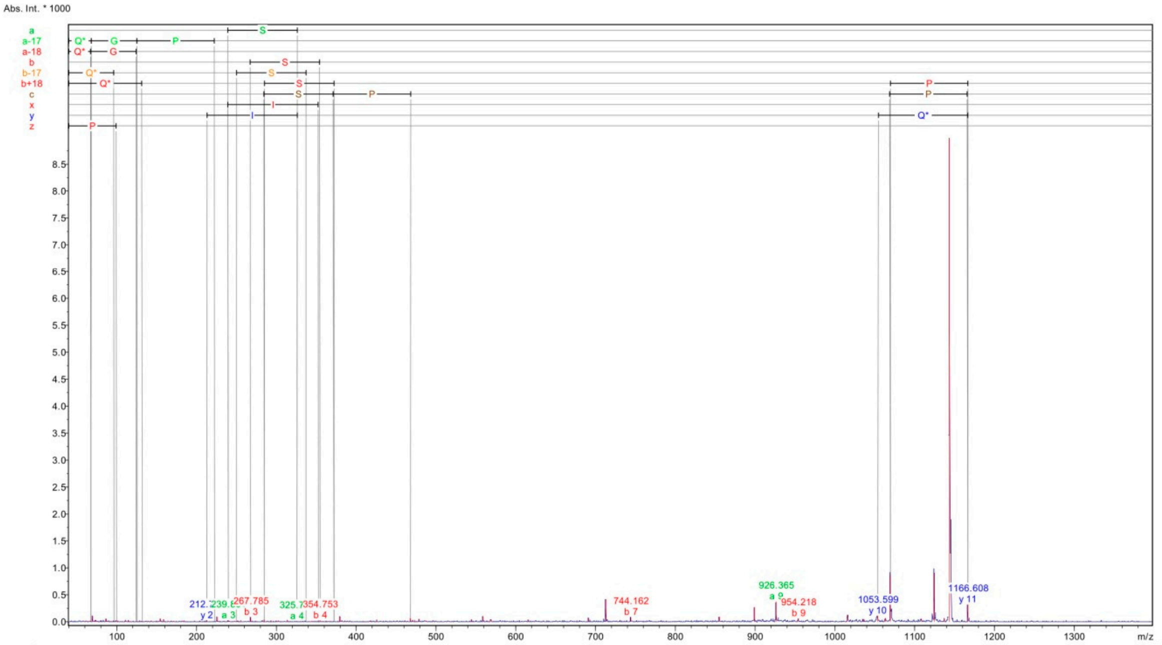

Copyright © 1999-2008 Bruker Daltonik GmbH

Spectrum Analysis Report

Display Parameter:

Parentmass: 1166.597
Threshold (a.i.): 0.000
Assigned Peaks: 30
Mass Error: 0.001
Tolerance (Da): 1.000
Not assigned Peaks: 111
MH+ (mono): 1166.596
Number of Peaks: 141
MH+ (avg): 1167.296
Above Threshold: 141

Peaklist:

Peak	Mass	Intensity	Peak	Mass	Intensity	Peak	Mass	Intensity	Peak	Mass	Intensity	Peak	Mass	Intensity
1	59.306	9.477	2	60.819	12.672	3	63.192	13.250	4	65.591	10.865	5	67.492	14.744
6	69.794	107.891	7	72.735	14.595	8	74.072	31.349	9	80.464	23.295	10	86.752	55.381
11	89.890	19.272	12	93.690	12.222	13	96.577	21.788	14	99.775	23.860	15	101.639	16.087
16	104.836	6.643	17	107.141	8.810	18	110.296	14.562	19	111.579	22.022	20	114.775	26.071
21	119.736	9.138	22	121.276	10.065	23	123.918	8.984	24	125.231	7.220	25	128.773	23.393
26	131.859	10.166	27	137.308	11.730	28	138.874	12.022	29	140.924	10.460	30	143.727	14.871
31	146.646	4.862	32	147.852	7.474	33	150.586	18.429	34	154.751	58.099	35	158.793	42.149
36	163.839	14.872	37	165.092	12.050	38	168.616	16.150	39	171.840	9.737	40	174.796	12.252
41	178.788	10.726	42	182.080	16.000	43	184.415	21.672	44	191.968	3.602	45	194.752	5.752
46	197.799	15.471	47	204.293	16.232	48	210.756	14.742	49	212.778	26.646	50	217.278	3.860
51	222.439	7.715	52	225.803	93.920	53	229.752	10.936	54	234.758	12.763	55	239.801	27.279
56	243.095	8.438	57	244.869	9.336	58	247.779	10.010	59	249.514	11.025	60	251.893	17.873
61	255.449	24.211	62	259.659	4.572	63	261.147	7.694	64	264.568	9.143	65	267.785	88.442
66	271.496	8.291	67	274.164	15.231	68	282.755	25.038	69	284.817	35.509	70	289.182	10.270
71	290.786	10.666	72	293.353	20.017	73	296.826	11.260	74	300.105	18.600	75	301.579	10.885
76	302.979	15.553	77	305.759	9.271	78	313.820	30.259	79	317.909	16.344	80	325.789	11.124
81	329.291	6.289	82	333.557	11.213	83	337.134	5.382	84	341.743	9.032	85	343.940	12.481
86	345.709	12.821	87	348.057	14.205	88	350.480	11.895	89	352.783	15.326	90	354.753	28.913
91	357.907	6.745	92	359.711	8.221	93	362.807	15.377	94	365.359	6.978	95	367.499	12.685
96	369.749	7.658	97	372.150	10.255	98	377.259	13.995	99	379.863	100.028	100	382.451	21.046
101	386.128	17.533	102	393.005	14.002	103	408.667	9.814	104	413.921	9.633	105	416.950	9.496
106	418.841	19.720	107	425.878	38.938	108	431.922	12.020	109	441.857	14.626	110	461.885	17.502
111	467.923	68.734	112	470.873	29.134	113	473.070	26.235	114	479.000	52.465	115	486.868	17.069
116	488.073	17.149	117	496.675	22.120	118	515.991	15.538	119	544.879	43.826	120	558.947	102.705
121	564.157	19.419	122	568.011	45.699	123	569.942	19.333	124	616.065	35.577	125	691.245	71.120
126	713.091	418.546	127	744.162	87.912	128	855.207	98.162	129	899.272	273.492	130	926.365	378.152
131	954.218	66.567	132	1015.370	129.188	133	1035.437	54.592	134	1053.599	114.444	135	1063.265	68.583
136	1069.531	860.630	137	1108.719	51.857	138	1124.650	909.722	139	1137.283	62.850	140	1143.502	9274.172
141	1166.608	324.549												

Calculated Masses:

QGPSPRHPIPP N-Term: Gln->pyro-Glu (N-term Q) 1: Deamidated (NQ)

N-Term.	Ion	a	a-17	a-18	b	b-17	b-18	b+18	c	i	x	y	z	C-Term.	Ion
1	Q*	85.028	**68.002**	**67.018**	113.023	**95.997**	95.013	**131.034**	130.050	85.028	142.050	116.071	**99.044**	11	P
2	G	142.050	**125.023**	**124.039**	170.045	153.018	152.034	188.055	187.071	30.034	**239.103**	**213.123**	196.097	10	P
3	P	**239.103**	**222.076**	221.092	**267.098**	**250.071**	**249.087**	**285.108**	**284.124**	**70.065**	**352.187**	**326.207**	309.181	9	I
4	S	**326.135**	309.108	308.124	**354.130**	**337.103**	336.119	**372.140**	**371.156**	**60.044**	449.239	423.260	406.234	8	P
5	P	423.187	406.161	405.177	451.182	434.156	433.172	469.193	**468.209**	**70.065**	586.298	560.319	543.293	7	H
6	R	579.289	562.262	561.278	607.283	590.257	589.273	625.294	624.310	**129.113**	742.399	716.420	699.394	6	R
7	H	716.347	699.321	698.337	**744.342**	727.316	726.332	762.353	761.369	**110.071**	839.452	813.473	796.446	5	P
8	P	813.400	796.374	795.390	841.395	824.369	823.385	859.406	858.422	**70.065**	**926.484**	900.505	883.478	4	S
9	I	**926.484**	909.458	908.474	**954.479**	937.453	936.469	972.490	971.506	**86.096**	1023.537	997.558	980.531	3	P
10	P	1023.537	1006.510	1005.526	1051.532	**1034.505**	1033.521	**1069.543**	**1068.559**	**70.065**	1080.559	**1054.579**	1037.553	2	G
11	P	1120.590	1103.563	1102.579	1148.585	1131.558	1130.574	**1166.595**	**1165.611**	**70.065**	1192.575	**1166.595**	1149.569	1	Q*

Copyright © 1999-2008 Bruker Daltonik GmbH

Annotated MS/MS spectrum of peptide 1182.573 *m*/*z*.

Spectrum Analysis Report

Sequence Name:		Parentmass:	1182.573	Mass Error:	-0.963	MH+ (mono):	1183.536
MH+ (avg):	1184.350	Threshold (a.i.):	0.000	Tolerance (Da):	3.000	Number of Peaks:	114
Above Threshold:	114	Assigned Peaks:	40	Not assigned Peaks:	74		

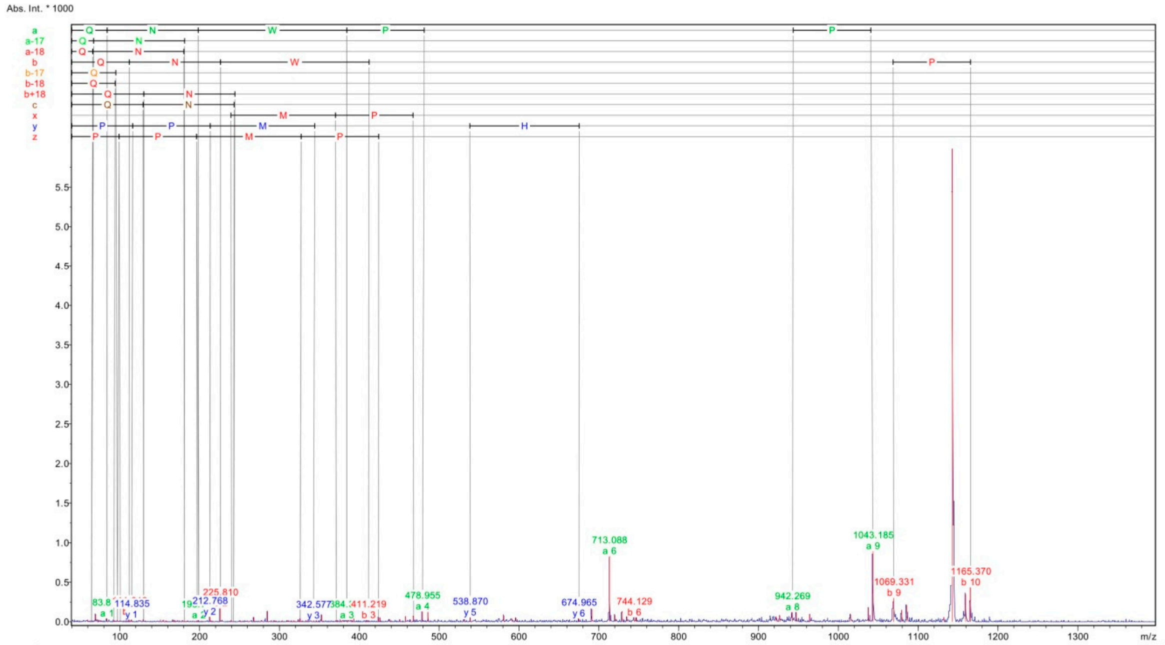

BioTools Version 3.2 Copyright © 1999-2008 Bruker Daltonik GmbH Page -1-

Spectrum Analysis Report

Display Parameter:

Parentmass:	1182.573	Mass Error:	-0.963	MH+ (mono):	1183.536	MH+ (avg):	1184.350
Threshold (a.i.):	0.000	Tolerance (Da):	3.000	Number of Peaks:	114	Above Threshold:	114
Assigned Peaks:	40	Not assigned Peaks:	74				

Peaklist:

Peak	Mass	Intensity	Peak	Mass	Intensity	Peak	Mass	Intensity	Peak	Mass	Intensity	Peak	Mass	Intensity
1	59.738	21.980	2	64.404	16.865	3	69.812	97.501	4	72.703	25.033	5	76.280	18.907
6	83.811	41.387	7	92.307	24.307	8	96.871	22.622	9	100.545	15.285	10	106.858	14.858
11	109.394	13.614	12	111.812	63.484	13	114.835	25.031	14	124.833	16.267	15	129.757	27.579
16	150.834	25.776	17	154.739	21.785	18	157.421	13.107	19	158.703	12.724	20	166.723	24.038
21	169.747	18.001	22	172.653	16.502	23	176.184	7.771	24	178.743	16.026	25	180.986	10.992
26	182.831	9.146	27	184.781	10.458	28	191.772	9.801	29	194.649	7.196	30	196.760	10.018
31	198.755	18.971	32	203.255	9.517	33	212.768	62.957	34	215.064	11.132	35	216.774	9.273
36	220.819	8.198	37	225.810	169.455	38	239.813	18.822	39	242.329	8.540	40	250.796	17.038
41	256.062	11.499	42	260.091	11.694	43	261.330	7.446	44	262.802	8.699	45	267.788	59.656
46	273.543	10.665	47	277.796	19.836	48	284.825	136.202	49	289.781	15.045	50	293.566	16.500
51	295.860	10.991	52	303.815	11.872	53	309.787	18.137	54	311.819	16.756	55	315.431	13.160
56	318.123	14.283	57	319.546	13.888	58	323.850	27.902	59	325.839	40.297	60	329.232	12.764
61	332.837	16.472	62	335.803	25.663	63	339.318	18.929	64	342.577	15.475	65	346.276	20.762
66	349.781	17.153	67	352.824	91.071	68	371.238	15.684	69	373.339	18.414	70	376.311	33.532
71	379.879	115.441	72	384.336	18.710	73	386.670	33.673	74	389.739	48.834	75	393.272	40.403
76	408.888	24.178	77	411.219	18.903	78	423.839	58.021	79	425.818	52.234	80	437.816	31.965
81	450.851	31.421	82	457.859	71.444	83	463.975	26.964	84	467.840	79.866	85	478.955	130.080
86	485.876	126.566	87	531.686	29.667	88	538.870	54.170	89	580.896	79.638	90	591.075	39.147
91	596.019	53.026	92	674.965	40.964	93	691.134	159.833	94	713.088	829.701	95	719.963	84.034
96	729.025	134.443	97	735.094	66.562	98	744.129	54.103	99	747.049	56.575	100	922.263	67.406
101	926.563	80.644	102	942.269	121.769	103	947.260	123.777	104	964.168	98.663	105	1015.269	95.083
106	1037.379	181.966	107	1043.185	891.616	108	1069.331	300.911	109	1079.170	148.409	110	1085.424	215.995
111	1132.158	60.423	112	1142.662	6034.370	113	1159.254	348.867	114	1165.370	433.160			

Calculated Masses:
QNWPHPPMPP N-Term: Gln->pyro-Glu (N-term Q)

N-Term.	Ion	a	a-17	a-18	b	b-17	b-18	b+18	c	i	x	y	z	C-Term.	Ion
1	Q	**84.044**	**67.018**	**66.034**	**112.039**	**95.013**	**94.029**	**130.050**	**129.066**	**84.044**	142.050	**116.071**	**99.044**	10	P
2	N	**198.087**	**181.061**	**180.077**	**226.082**	209.056	208.072	**244.093**	**243.109**	87.055	**239.103**	**213.123**	**196.097**	9	P
3	W	**384.167**	367.140	366.156	**412.162**	**395.135**	**394.151**	430.172	429.188	**159.092**	**370.143**	**344.164**	**327.137**	8	M
4	P	**481.219**	**464.193**	**463.209**	509.214	492.188	491.204	527.225	526.241	**70.065**	**467.196**	441.217	**424.190**	7	P
5	H	618.278	601.252	600.268	646.273	629.247	628.263	664.284	663.300	**110.071**	564.249	**538.269**	521.243	6	P
6	P	**715.331**	698.305	697.321	**743.326**	**726.299**	725.315	761.337	760.353	**70.065**	701.308	**675.328**	658.302	5	H
7	P	812.384	795.357	794.373	840.379	823.352	822.368	858.389	857.405	**70.065**	798.360	772.381	755.355	4	P
8	M	**943.424**	**926.398**	**925.414**	971.419	954.393	953.409	989.430	988.446	**104.053**	984.440	958.460	**941.434**	3	W
9	P	**1040.477**	1023.451	1022.467	**1068.472**	1051.445	1050.461	**1086.483**	**1085.499**	**70.065**	1098.483	1072.503	1055.477	2	N
10	P	1137.530	1120.503	1119.519	**1165.525**	1148.498	1147.514	1183.535	1182.551	**70.065**	1209.515	1183.535	**1166.509**	1	Q

BioTools Version 3.2 Copyright © 1999-2008 Bruker Daltonik GmbH Page -2-
